# Supplementary material for: Temporal shifts in 24 notifiable infectious diseases in China before and during the COVID-19 pandemic
Source: Nat Commun. 2024 May 8;15:3891. doi: 10.1038/s41467-024-48201-8 (PMC11079007; doi:10.1038/s41467-024-48201-8)
Supplement: Supplementary file 1 — Supplementary Information [file 41467_2024_48201_MOESM1_ESM.pdf]

Supplementary Information for

Temporal shifts in 24 notifiable infectious diseases in China before and during the COVID-19 pandemic

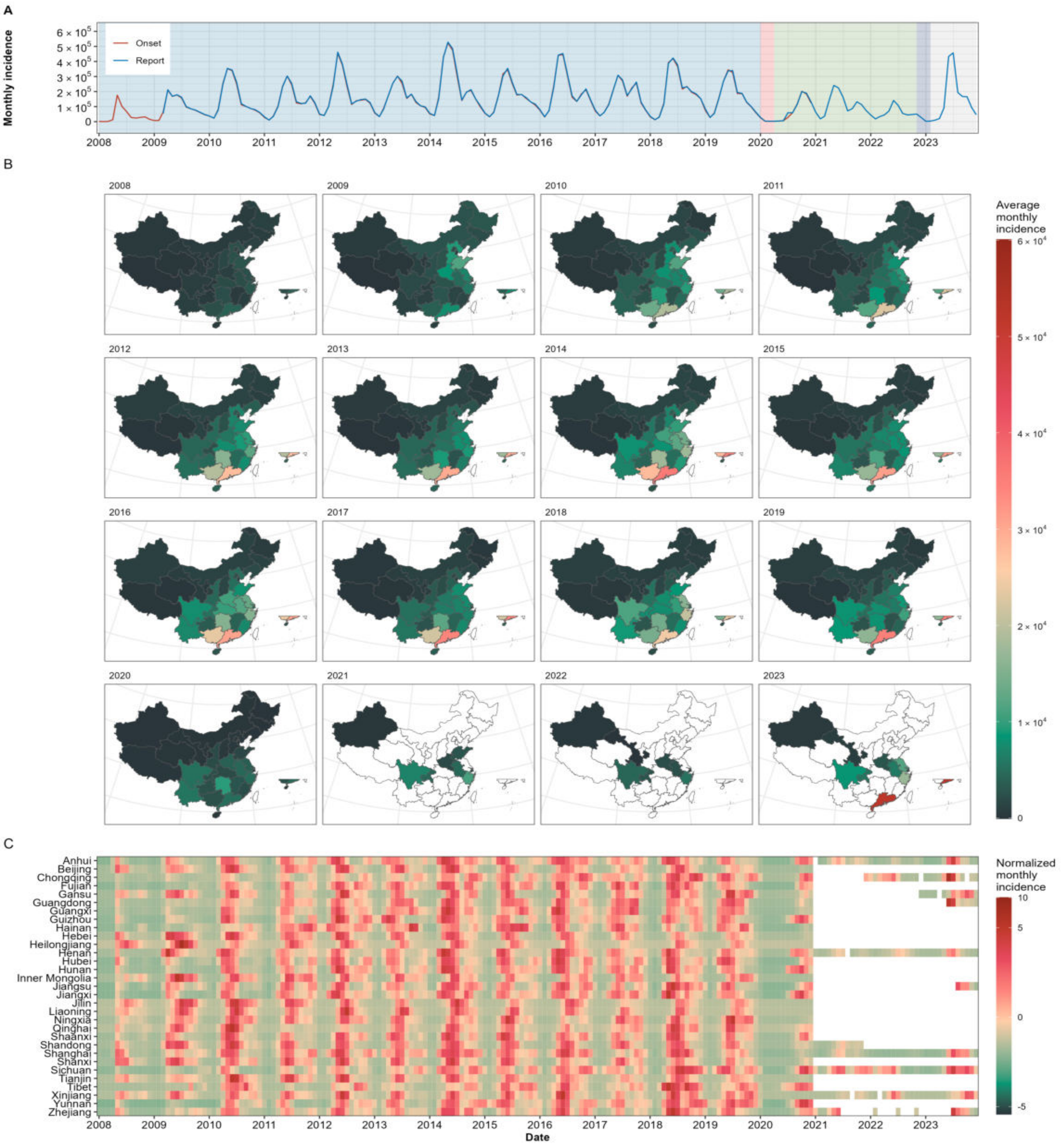

Supplementary Fig. 1. Temporal variation in monthly incidence of hand, foot, and mouth disease (HFMD) from January 2008 to December 2023 in China.

(A) The incidence of hand, foot, and mouth disease (HFMD) in China from January 2008 to December 2023; **(B)** The spatial distribution of cases in China; **(C)** Temporal variation in monthly incidence among different provinces. The heatmap represents the normalized monthly incidence data of each province, and the color intensity corresponds to the normalized monthly incidence. Provincial data in panel **(B)** and **(C)** before January 2020 sourced from the Chinese Public Health Science Data Center, and data after January 2020 sourced from the provincial Notifiable Infectious Diseases Reports. * Normalized monthly incidence > 10.

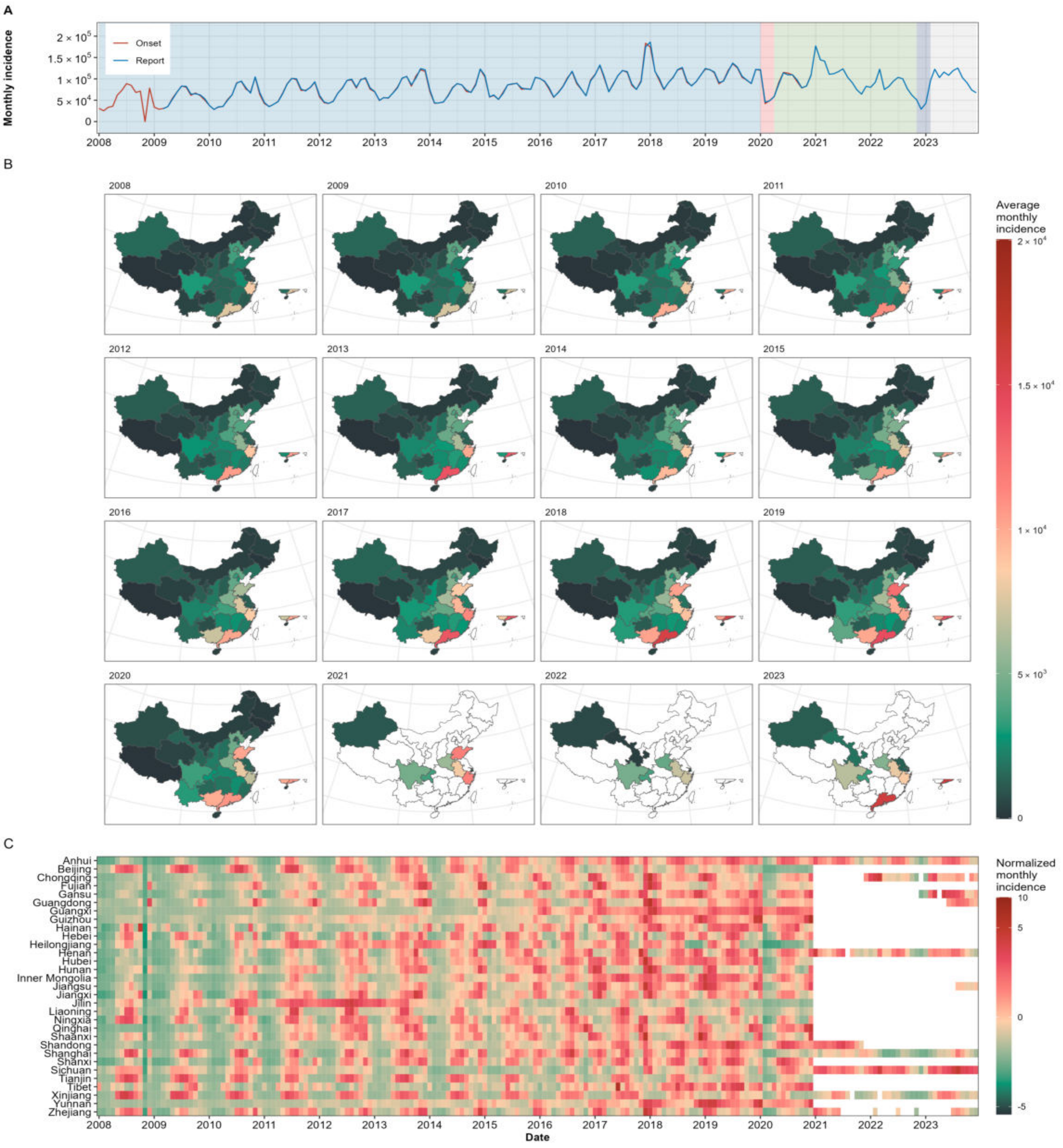

Supplementary Fig. 2. Temporal variation in monthly incidence of infectious diarrhea from January 2008 to December 2023 in China.

(A) The incidence of infectious diarrhea in China from January 2008 to December 2023; **(B)** The spatial distribution of cases in China; **(C)** Temporal variation in monthly incidence among different provinces. The heatmap represents the normalized monthly incidence data of each province, and the color intensity corresponds to the normalized monthly incidence. Provincial data in panel **(B)** and **(C)** before January 2020 sourced from the Chinese Public Health Science Data Center, and data after January 2020 sourced from the provincial Notifiable Infectious Diseases Reports. * Normalized monthly incidence > 10.

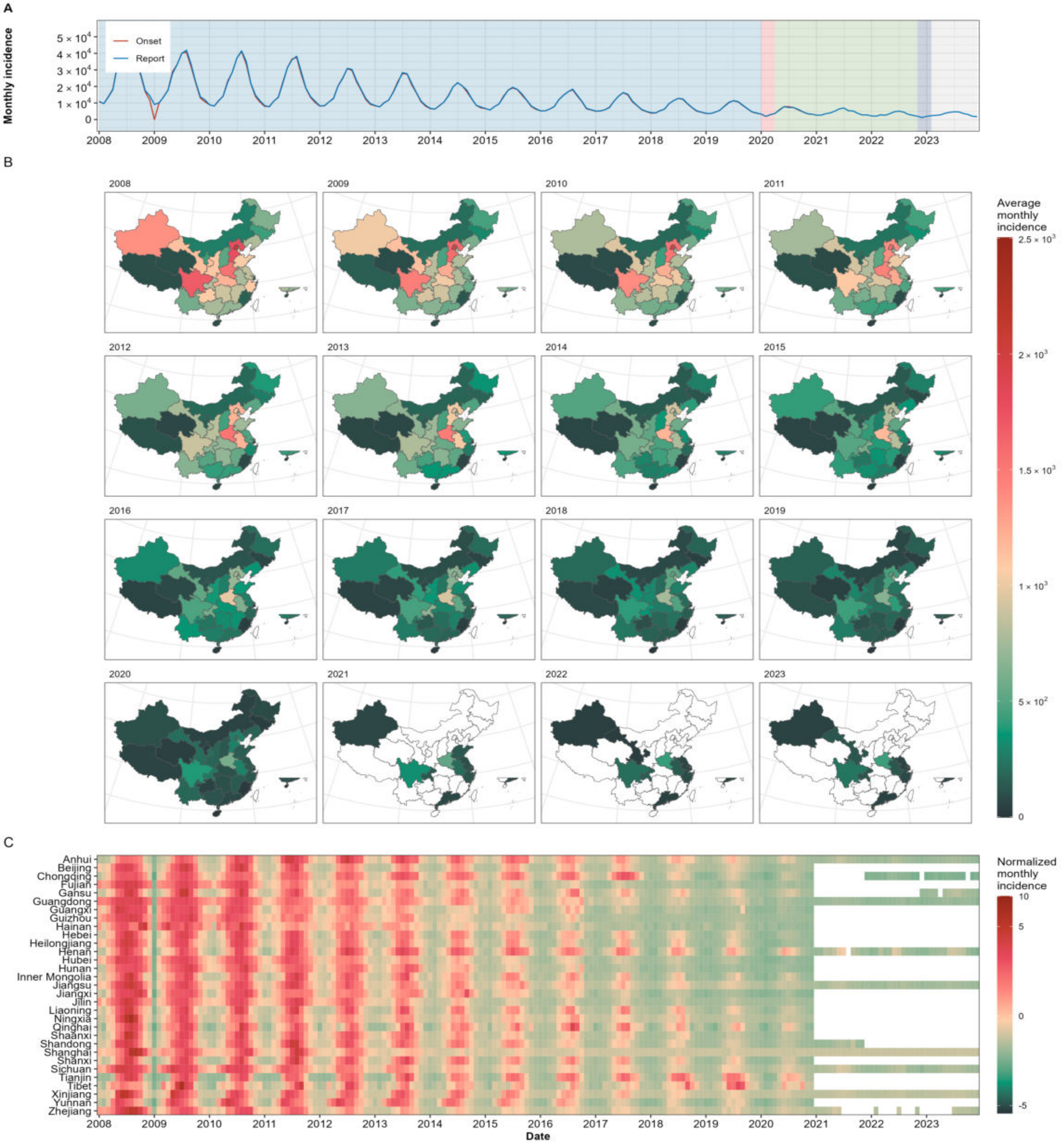

Supplementary Fig. 3. Temporal variation in monthly incidence of dysentery from January 2008 to December 2023 in China.

(A) The incidence of dysentery in China from January 2008 to December 2023; **(B)** The spatial distribution of cases in China; **(C)** Temporal variation in monthly incidence among different provinces. The heatmap represents the normalized monthly incidence data of each province, and the color intensity corresponds to the normalized monthly incidence. Provincial data in panel **(B)** and **(C)** before January 2020 sourced from the Chinese Public Health Science Data Center, and data after January 2020 sourced from the provincial Notifiable Infectious Diseases Reports. * Normalized monthly incidence > 10.

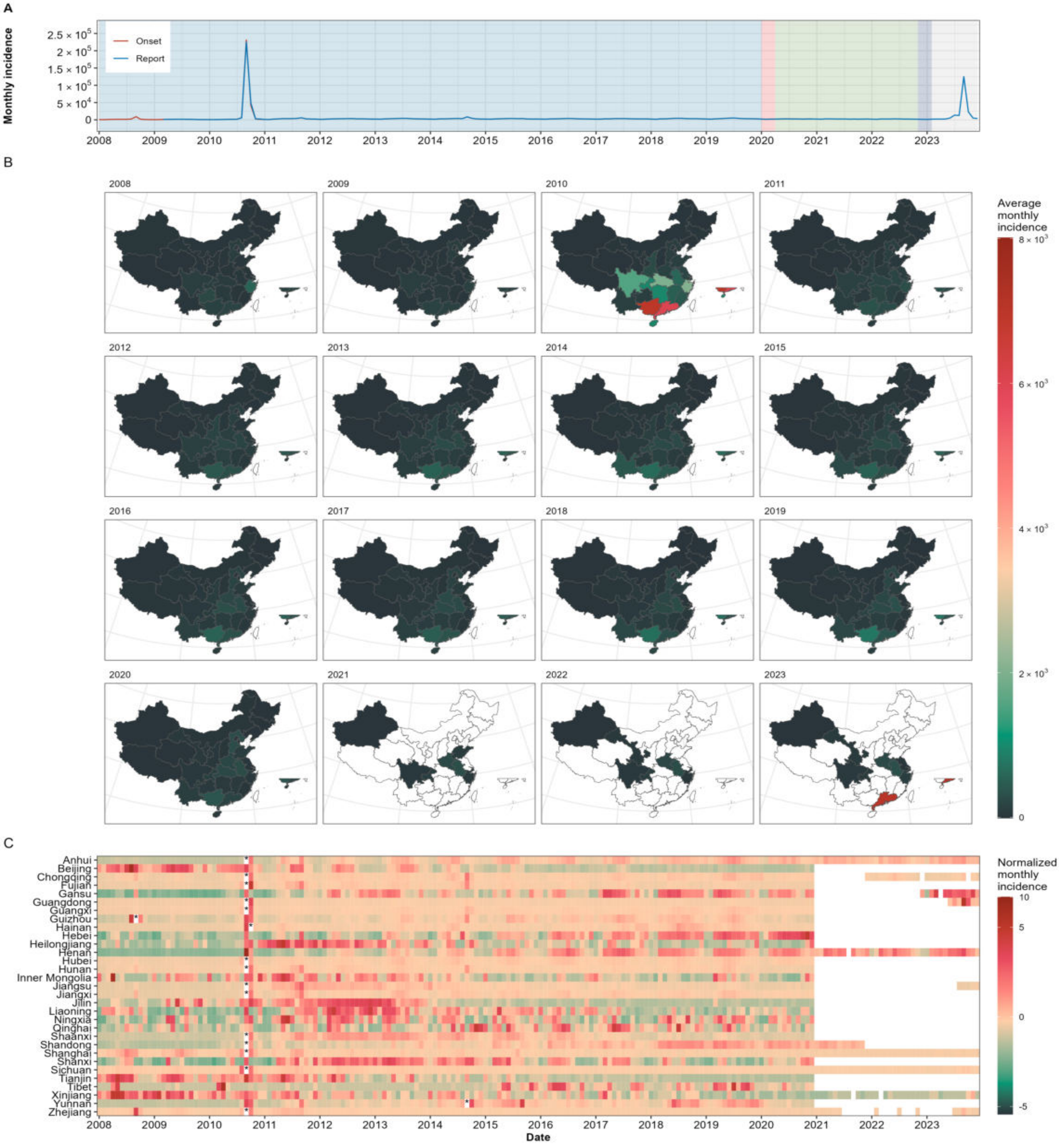

Supplementary Fig. 4. Temporal variation in monthly incidence of acute hemorrhagic conjunctivitis (AHC) from January 2008 to December 2023 in China.

(A) The incidence of acute hemorrhagic conjunctivitis (AHC) in China from January 2008 to December 2023; **(B)** The spatial distribution of cases in China; **(C)** Temporal variation in monthly incidence among different provinces. The heatmap represents the normalized monthly incidence data of each province, and the color intensity corresponds to the normalized monthly incidence. Provincial data in panel **(B)** and **(C)** before January 2020 sourced from the Chinese Public Health Science Data Center, and data after January 2020 sourced from the provincial Notifiable Infectious Diseases Reports. * Normalized monthly incidence > 10.

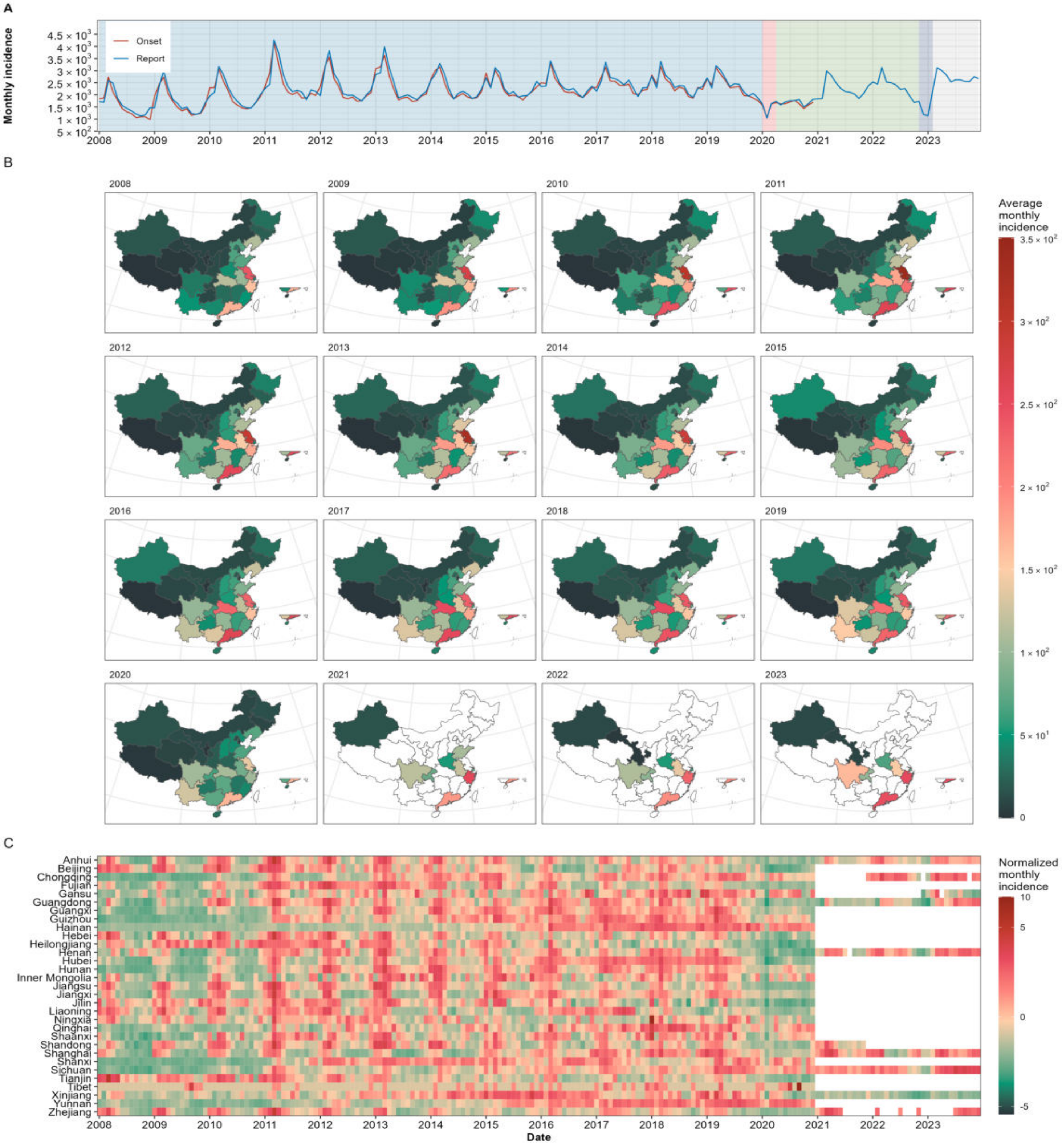

Supplementary Fig. 5. Temporal variation in monthly incidence of hepatitis E from January 2008 to December 2023 in China.

(A) The incidence of hepatitis E in China from January 2008 to December 2023; **(B)** The spatial distribution of cases in China; **(C)** Temporal variation in monthly incidence among different provinces. The heatmap represents the normalized monthly incidence data of each province, and the color intensity corresponds to the normalized monthly incidence. Provincial data in panel **(B)** and **(C)** before January 2020 sourced from the Chinese Public Health Science Data Center, and data after January 2020 sourced from the provincial Notifiable Infectious Diseases Reports. * Normalized monthly incidence > 10.

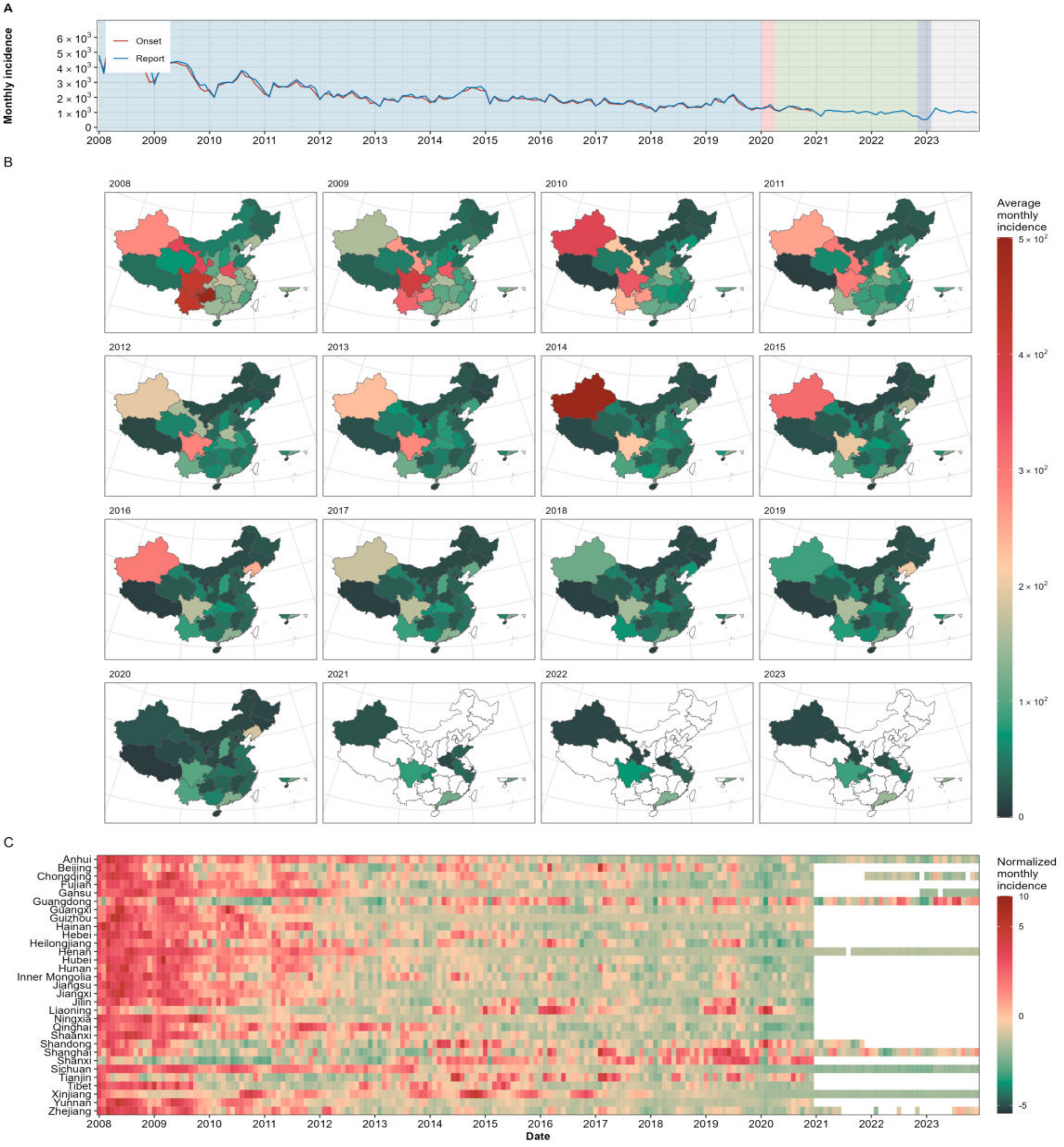

Supplementary Fig. 6. Temporal variation in monthly incidence of hepatitis A from January 2008 to December 2023 in China.

(A) The incidence of hepatitis A in China from January 2008 to December 2023; **(B)** The spatial distribution of cases in China; **(C)** Temporal variation in monthly incidence among different provinces. The heatmap represents the normalized monthly incidence data of each province, and the color intensity corresponds to the normalized monthly incidence. Provincial data in panel **(B)** and **(C)** before January 2020 sourced from the Chinese Public Health Science Data Center, and data after January 2020 sourced from the provincial Notifiable Infectious Diseases Reports. * Normalized monthly incidence > 10.

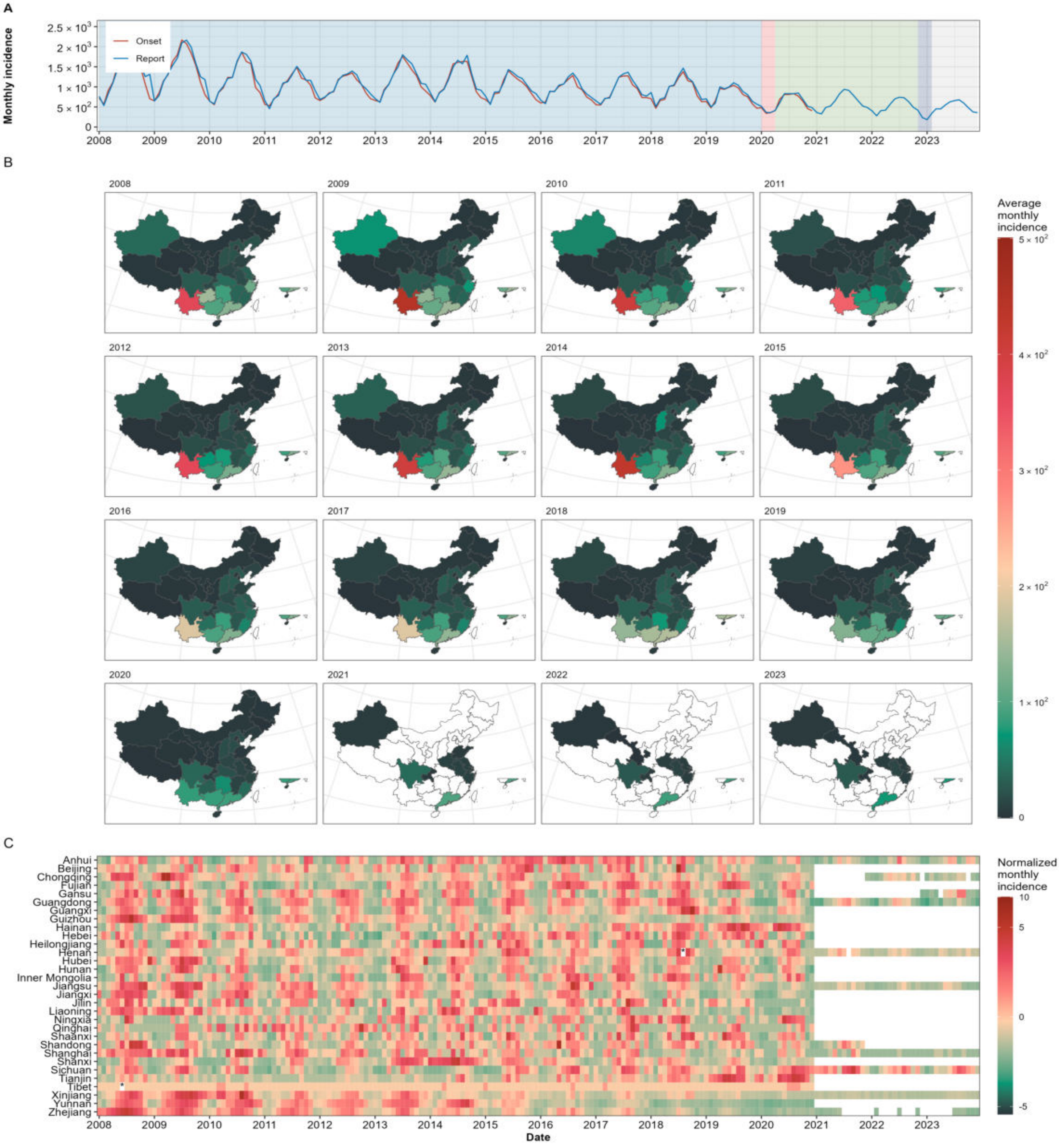

Supplementary Fig. 7. Temporal variation in monthly incidence of enteric fever from January 2008 to December 2023 in China.

(A) The incidence of enteric fever in China from January 2008 to December 2023; **(B)** The spatial distribution of cases in China; **(C)** Temporal variation in monthly incidence among different provinces. The heatmap represents the normalized monthly incidence data of each province, and the color intensity corresponds to the normalized monthly incidence. Provincial data in panel **(B)** and **(C)** before January 2020 sourced from the Chinese Public Health Science Data Center, and data after January 2020 sourced from the provincial Notifiable Infectious Diseases Reports. * Normalized monthly incidence > 10.

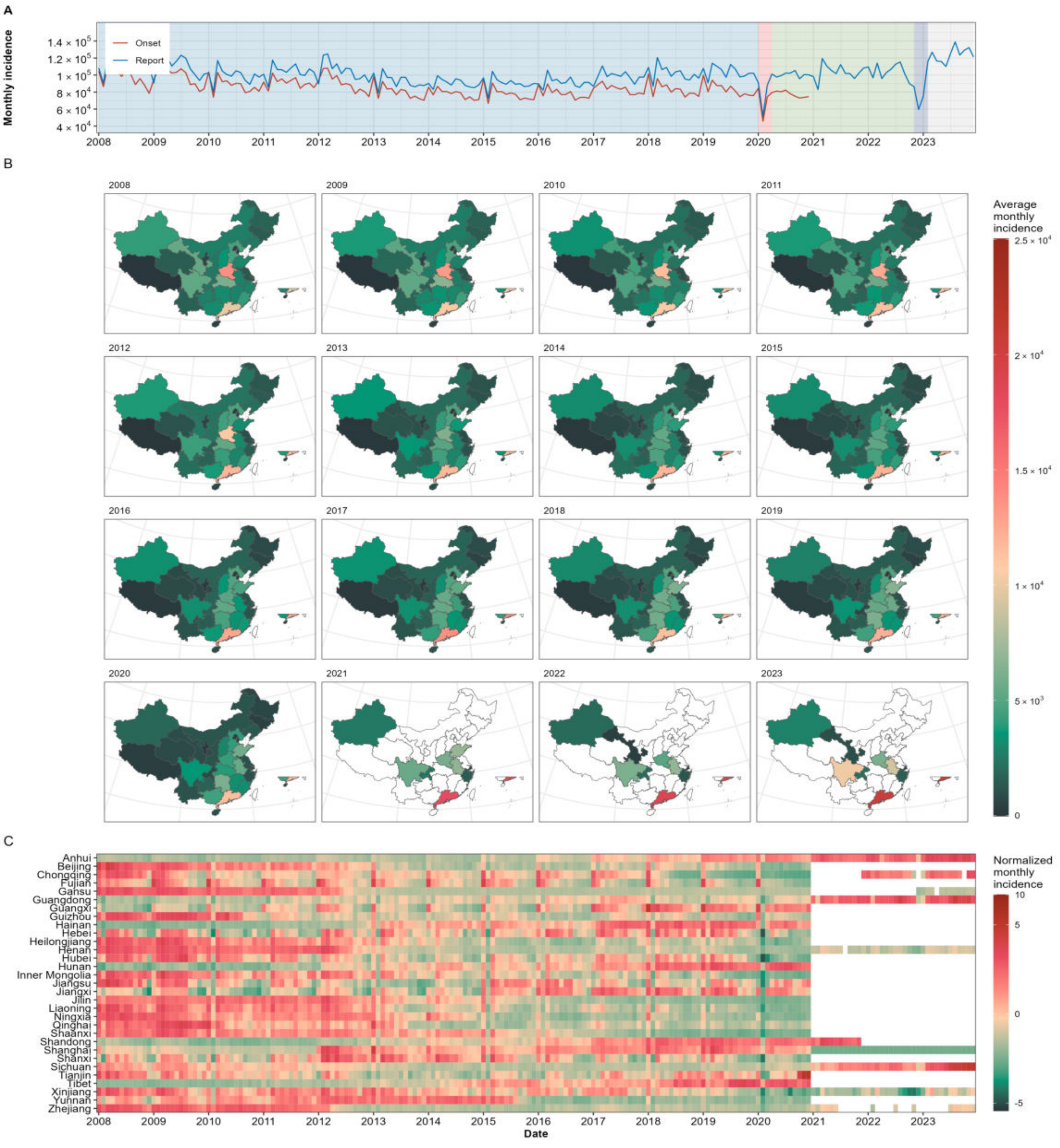

Supplementary Fig. 8. Temporal variation in monthly incidence of hepatitis B from January 2008 to December 2023 in China.

(A) The incidence of hepatitis B in China from January 2008 to December 2023; **(B)** The spatial distribution of cases in China; **(C)** Temporal variation in monthly incidence among different provinces. The heatmap represents the normalized monthly incidence data of each province, and the color intensity corresponds to the normalized monthly incidence. Provincial data in panel **(B)** and **(C)** before January 2020 sourced from the Chinese Public Health Science Data Center, and data after January 2020 sourced from the provincial Notifiable Infectious Diseases Reports. * Normalized monthly incidence > 10.

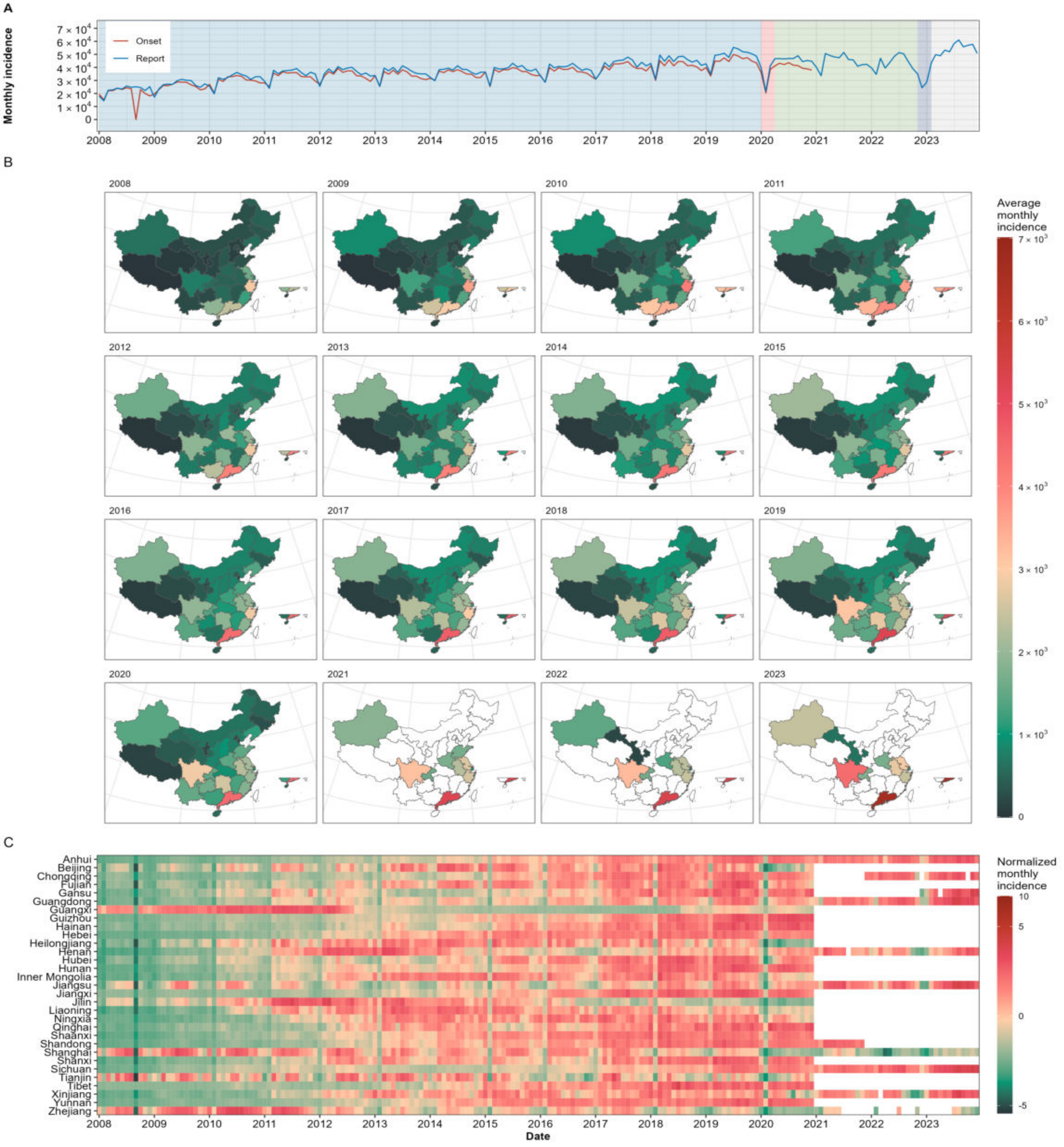

Supplementary Fig. 9. Temporal variation in monthly incidence of syphilis from January 2008 to December 2023 in China.

(A) The incidence of syphilis in China from January 2008 to December 2023; **(B)** The spatial distribution of cases in China; **(C)** Temporal variation in monthly incidence among different provinces. The heatmap represents the normalized monthly incidence data of each province, and the color intensity corresponds to the normalized monthly incidence. Provincial data in panel **(B)** and **(C)** before January 2020 sourced from the Chinese Public Health Science Data Center, and data after January 2020 sourced from the provincial Notifiable Infectious Diseases Reports. * Normalized monthly incidence > 10.

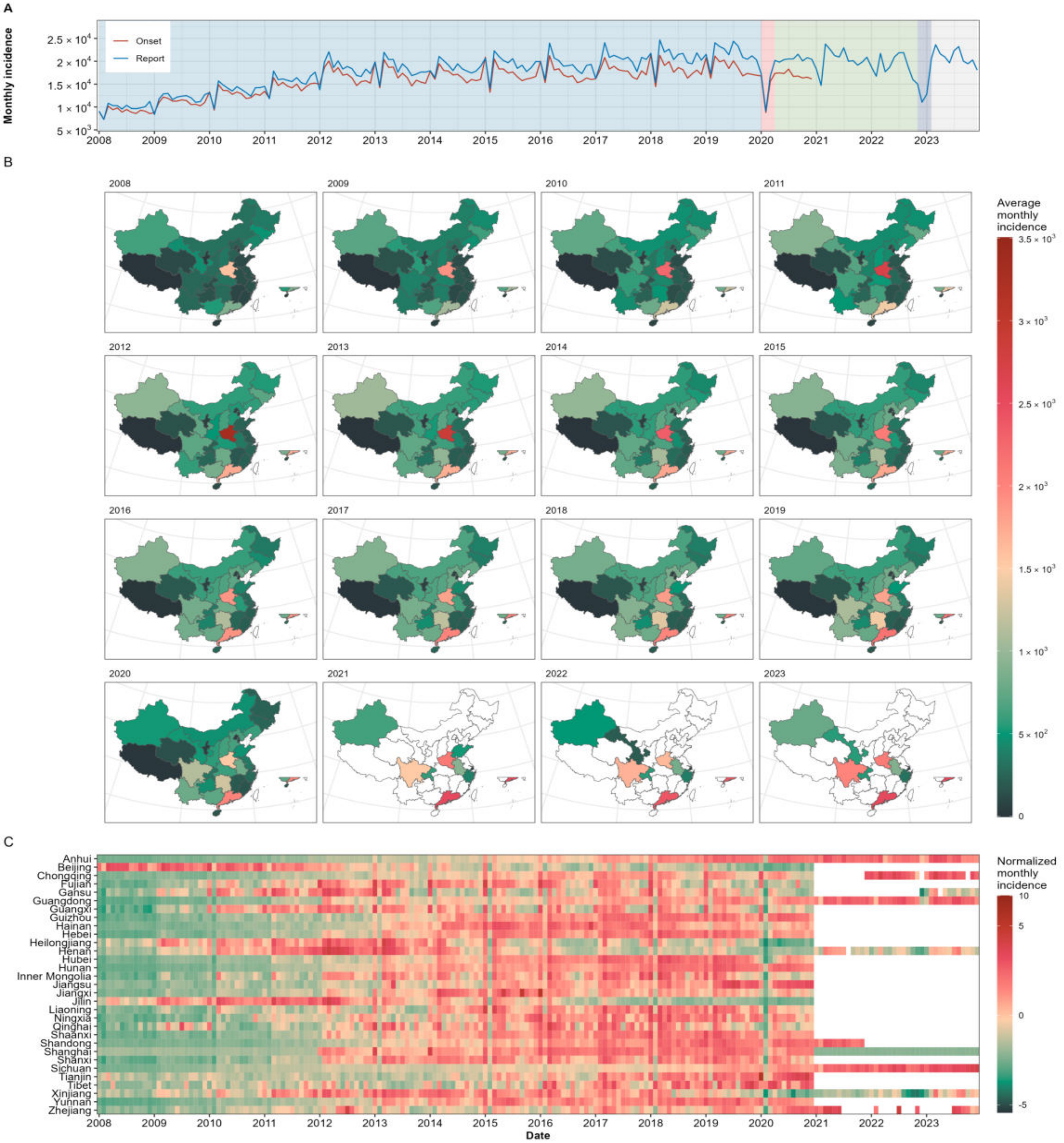

Supplementary Fig. 10. Temporal variation in monthly incidence of hepatitis C from January 2008 to December 2023 in China.

(A) The incidence of hepatitis C in China from January 2008 to December 2023; **(B)** The spatial distribution of cases in China; **(C)** Temporal variation in monthly incidence among different provinces. The heatmap represents the normalized monthly incidence data of each province, and the color intensity corresponds to the normalized monthly incidence. Provincial data in panel **(B)** and **(C)** before January 2020 sourced from the Chinese Public Health Science Data Center, and data after January 2020 sourced from the provincial Notifiable Infectious Diseases Reports. * Normalized monthly incidence > 10.

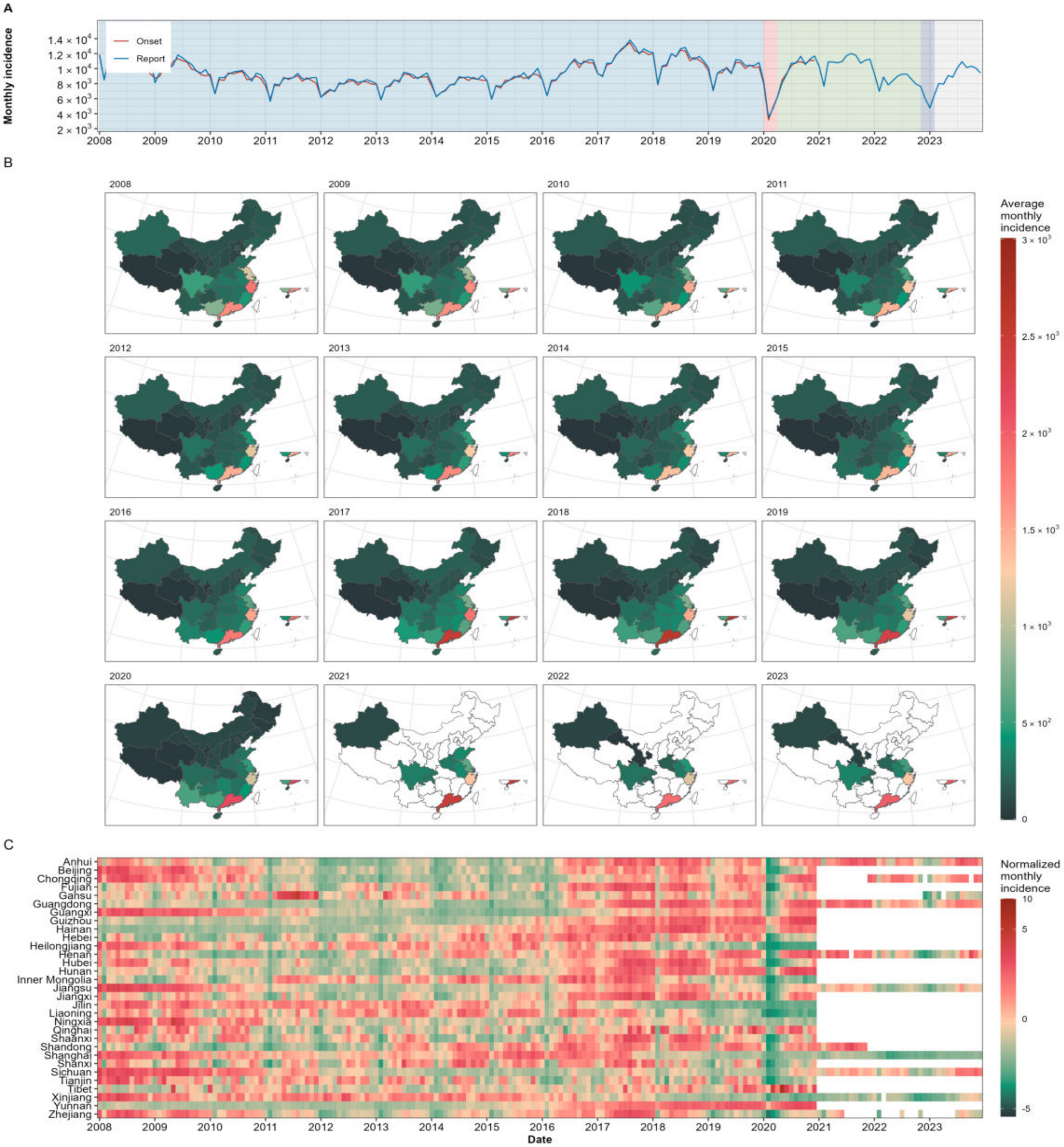

Supplementary Fig. 11. Temporal variation in monthly incidence of gonorrhea from January 2008 to December 2023 in China.

(A) The incidence of gonorrhea in China from January 2008 to December 2023; **(B)** The spatial distribution of cases in China; **(C)** Temporal variation in monthly incidence among different provinces. The heatmap represents the normalized monthly incidence data of each province, and the color intensity corresponds to the normalized monthly incidence. Provincial data in panel **(B)** and **(C)** before January 2020 sourced from the Chinese Public Health Science Data Center, and data after January 2020 sourced from the provincial Notifiable Infectious Diseases Reports. * Normalized monthly incidence > 10.

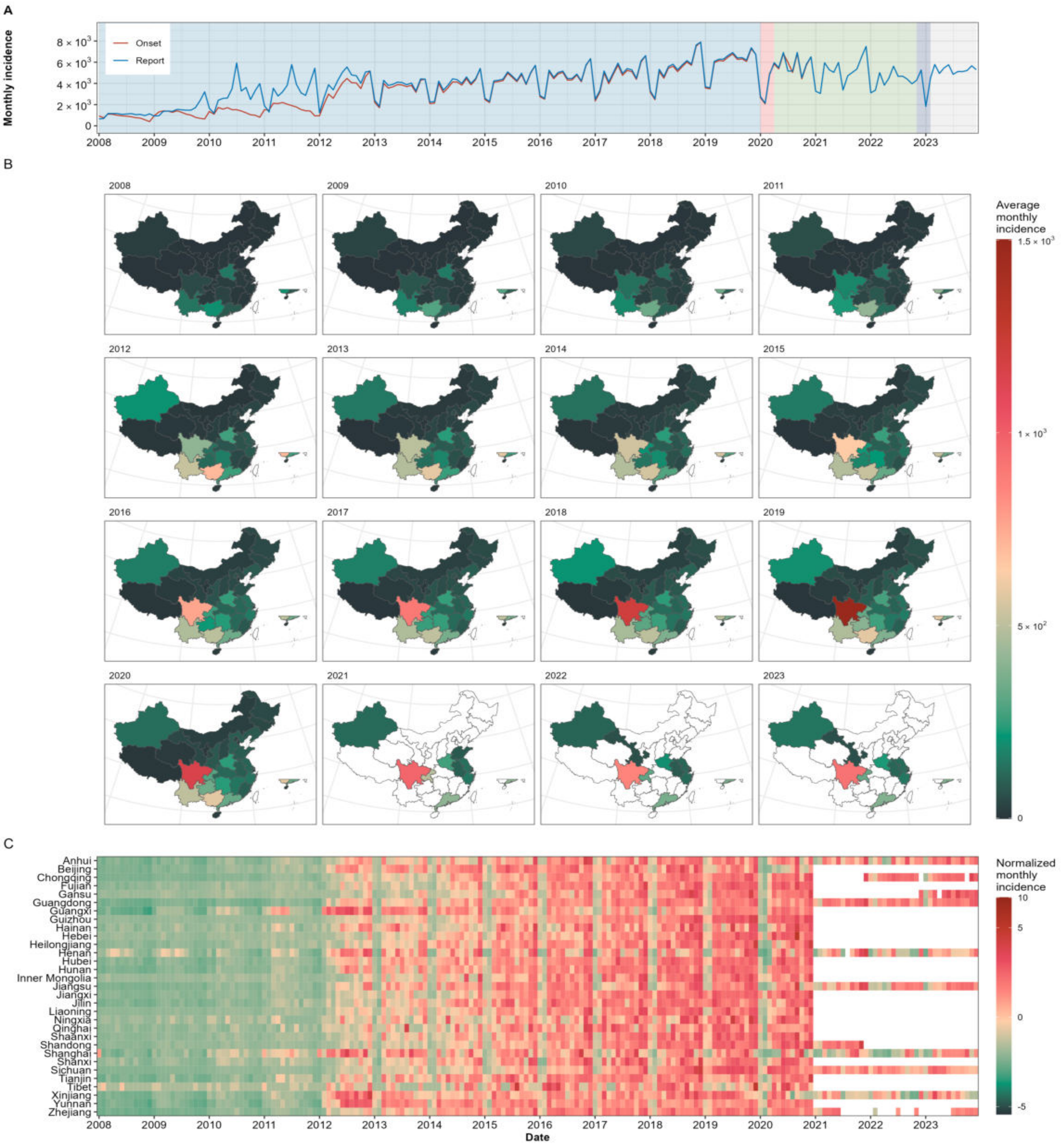

Supplementary Fig. 12. Temporal variation in monthly incidence of acquired immunodeficiency syndrome (AIDS) from January 2008 to December 2023 in China.

(A) The incidence of acquired immunodeficiency syndrome (AIDS) in China from January 2008 to December 2023; **(B)** The spatial distribution of cases in China; **(C)** Temporal variation in monthly incidence among different provinces. The heatmap represents the normalized monthly incidence data of each province, and the color intensity corresponds to the normalized monthly incidence. Provincial data in panel **(B)** and **(C)** before January 2020 sourced from the Chinese Public Health Science Data Center, and data after January 2020 sourced from the provincial Notifiable Infectious Diseases Reports. * Normalized monthly incidence > 10.

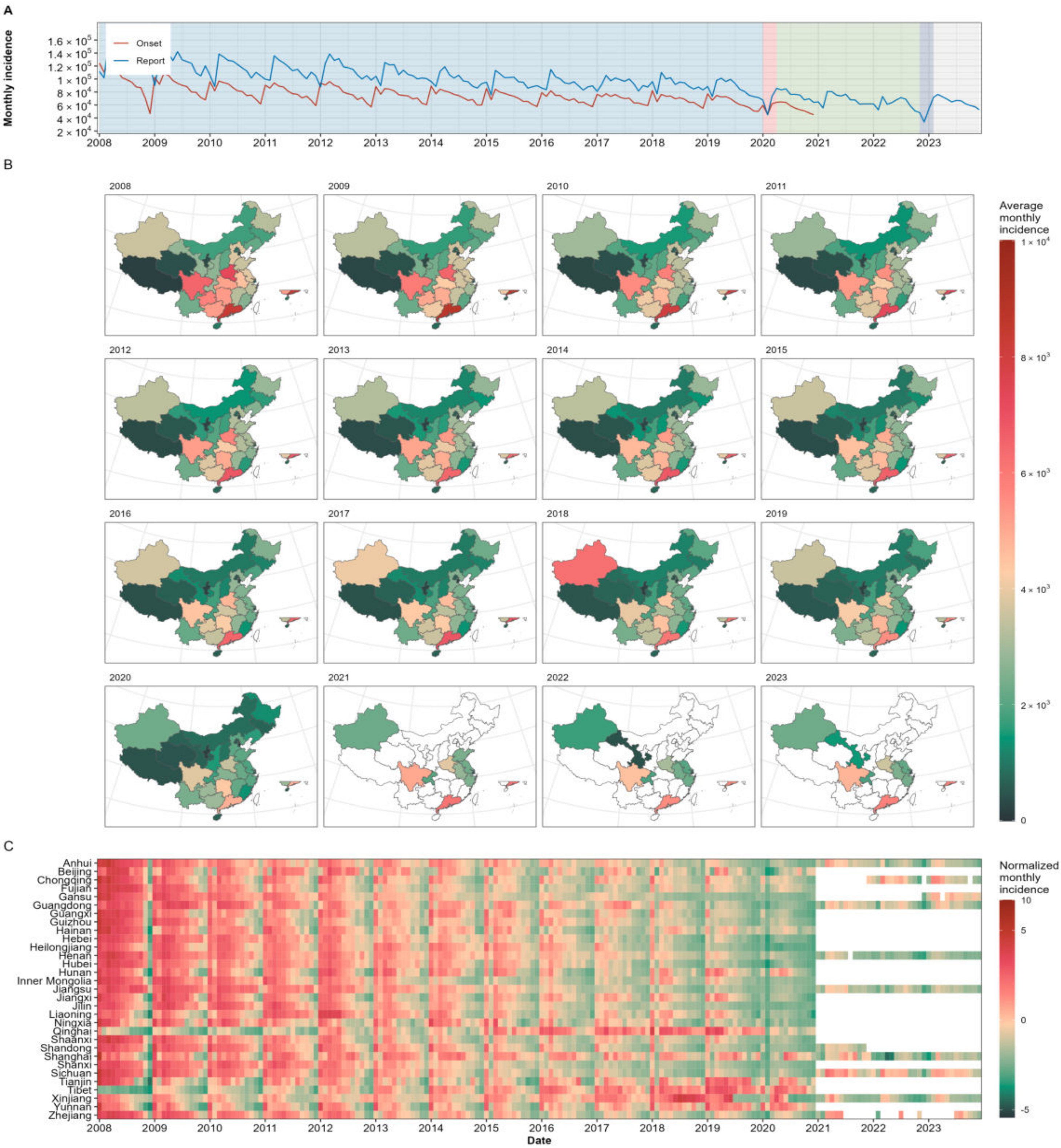

Supplementary Fig. 13. Temporal variation in monthly incidence of tuberculosis from January 2008 to December 2023 in China.

(A) The incidence of tuberculosis in China from January 2008 to December 2023; **(B)** The spatial distribution of cases in China; **(C)** Temporal variation in monthly incidence among different provinces. The heatmap represents the normalized monthly incidence data of each province, and the color intensity corresponds to the normalized monthly incidence. Provincial data in panel **(B)** and **(C)** before January 2020 sourced from the Chinese Public Health Science Data Center, and data after January 2020 sourced from the provincial Notifiable Infectious Diseases Reports. * Normalized monthly incidence > 10.

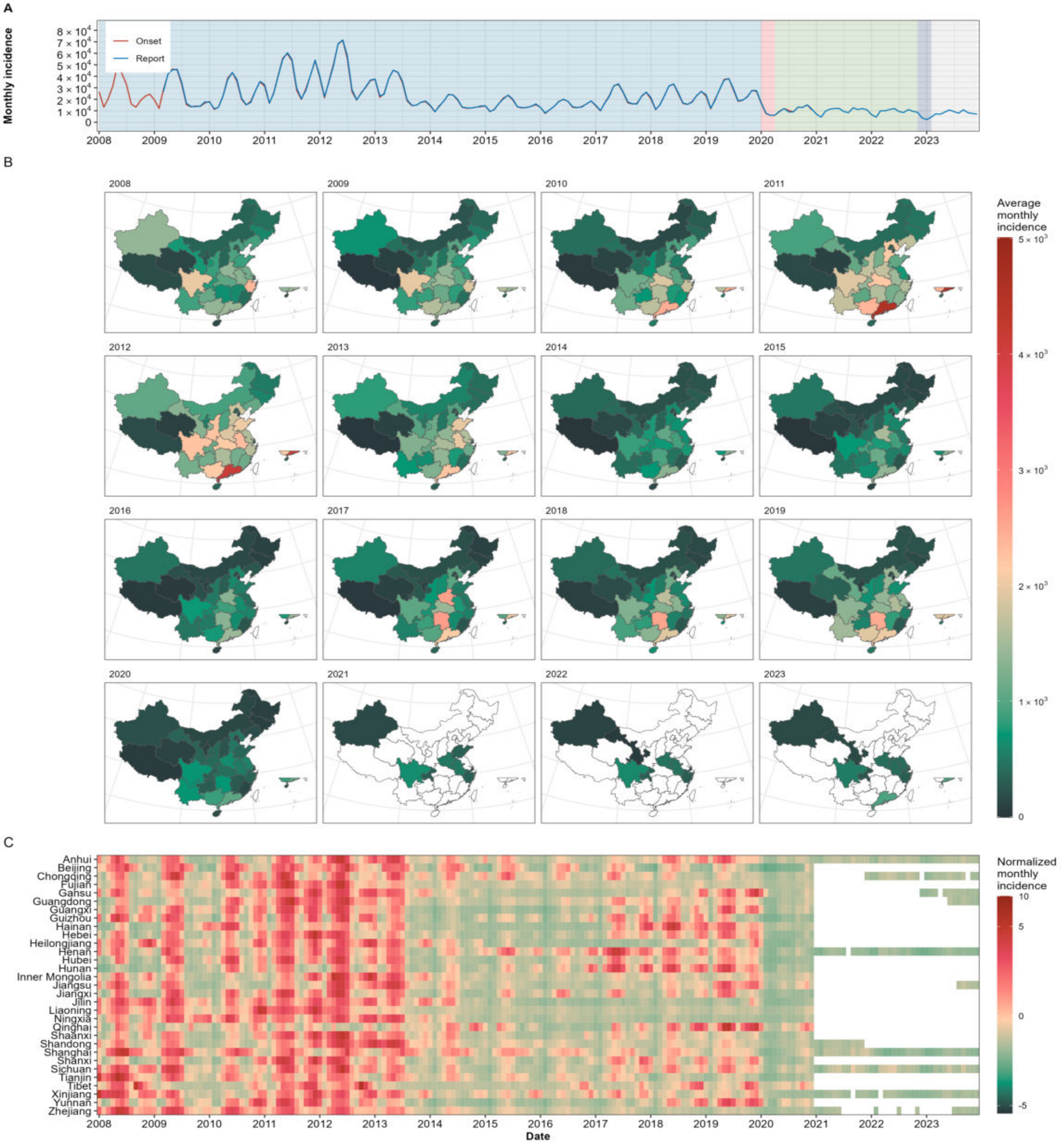

Supplementary Fig. 14. Temporal variation in monthly incidence of mumps from January 2008 to December 2023 in China.

(A) The incidence of mumps in China from January 2008 to December 2023; **(B)** The spatial distribution of cases in China; **(C)** Temporal variation in monthly incidence among different provinces. The heatmap represents the normalized monthly incidence data of each province, and the color intensity corresponds to the normalized monthly incidence. Provincial data in panel **(B)** and **(C)** before January 2020 sourced from the Chinese Public Health Science Data Center, and data after January 2020 sourced from the provincial Notifiable Infectious Diseases Reports. * Normalized monthly incidence > 10.

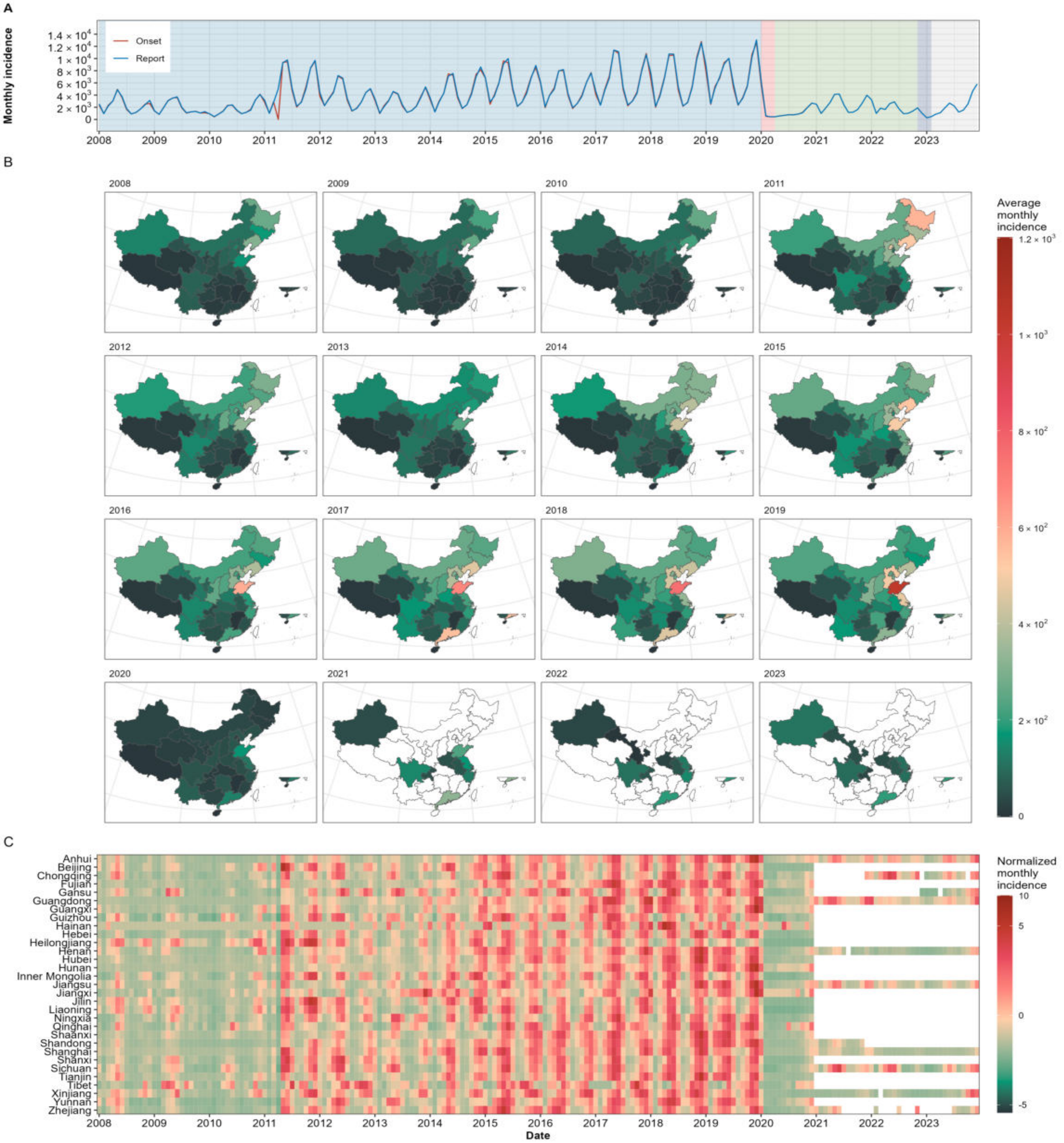

Supplementary Fig. 15. Temporal variation in monthly incidence of scarlet fever from January 2008 to December 2023 in China.

(A) The incidence of scarlet fever in China from January 2008 to December 2023; **(B)** The spatial distribution of cases in China; **(C)** Temporal variation in monthly incidence among different provinces. The heatmap represents the normalized monthly incidence data of each province, and the color intensity corresponds to the normalized monthly incidence. Provincial data in panel **(B)** and **(C)** before January 2020 sourced from the Chinese Public Health Science Data Center, and data after January 2020 sourced from the provincial Notifiable Infectious Diseases Reports. * Normalized monthly incidence > 10.

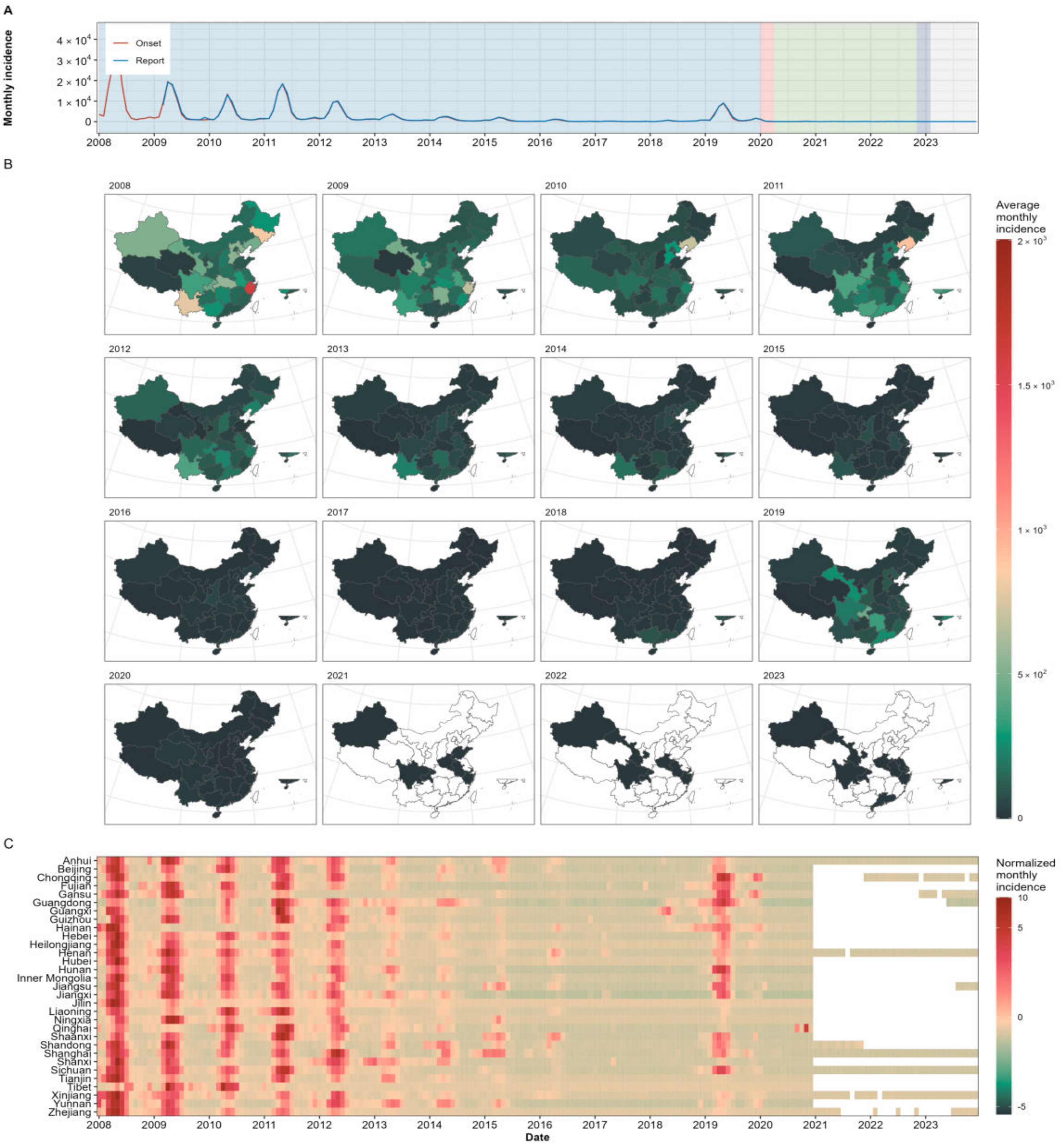

Supplementary Fig. 16. Temporal variation in monthly incidence of rubella from January 2008 to December 2023 in China.

(A) The incidence of rubella in China from January 2008 to December 2023; **(B)** The spatial distribution of cases in China; **(C)** Temporal variation in monthly incidence among different provinces. The heatmap represents the normalized monthly incidence data of each province, and the color intensity corresponds to the normalized monthly incidence. Provincial data in panel **(B)** and **(C)** before January 2020 sourced from the Chinese Public Health Science Data Center, and data after January 2020 sourced from the provincial Notifiable Infectious Diseases Reports. * Normalized monthly incidence > 10.

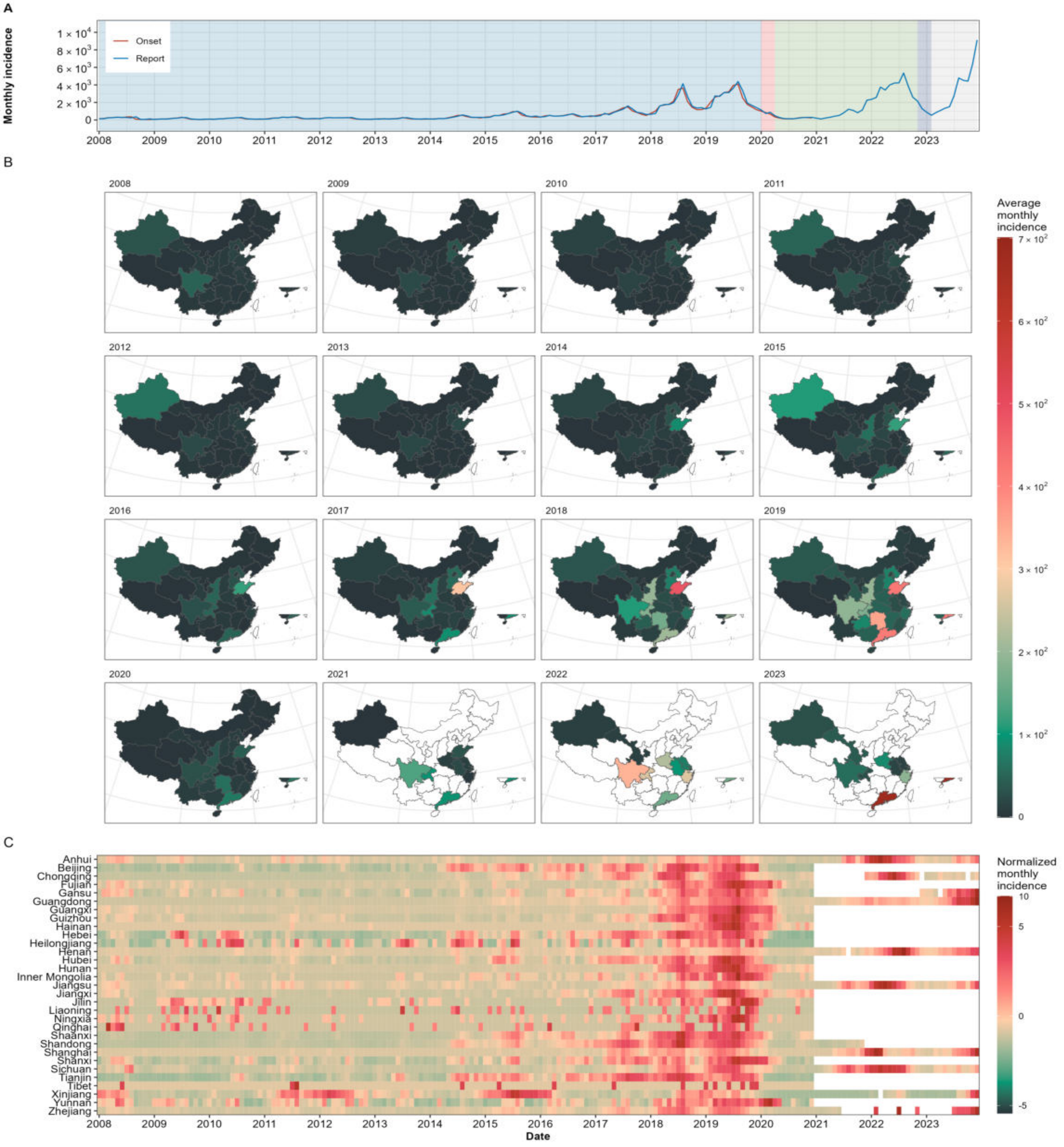

Supplementary Fig. 17. Temporal variation in monthly incidence of pertussis from January 2008 to December 2023 in China.

(A) The incidence of pertussis in China from January 2008 to December 2023; **(B)** The spatial distribution of cases in China; **(C)** Temporal variation in monthly incidence among different provinces. The heatmap represents the normalized monthly incidence data of each province, and the color intensity corresponds to the normalized monthly incidence. Provincial data in panel **(B)** and **(C)** before January 2020 sourced from the Chinese Public Health Science Data Center, and data after January 2020 sourced from the provincial Notifiable Infectious Diseases Reports. * Normalized monthly incidence > 10.

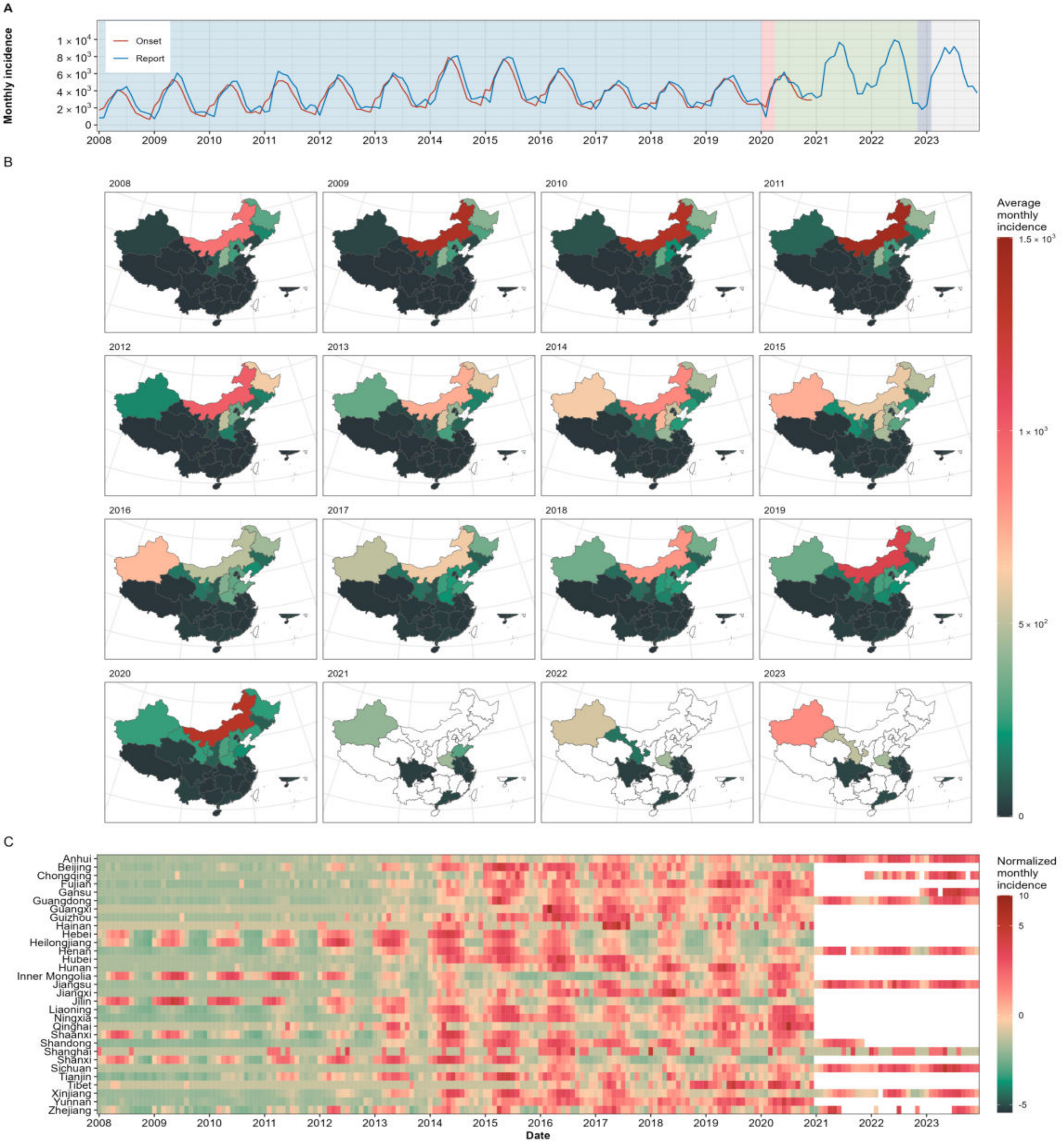

Supplementary Fig. 18. Temporal variation in monthly incidence of brucellosis from January 2008 to December 2023 in China.

(A) The incidence of brucellosis in China from January 2008 to December 2023; **(B)** The spatial distribution of cases in China; **(C)** Temporal variation in monthly incidence among different provinces. The heatmap represents the normalized monthly incidence data of each province, and the color intensity corresponds to the normalized monthly incidence. Provincial data in panel **(B)** and **(C)** before January 2020 sourced from the Chinese Public Health Science Data Center, and data after January 2020 sourced from the provincial Notifiable Infectious Diseases Reports. * Normalized monthly incidence > 10.

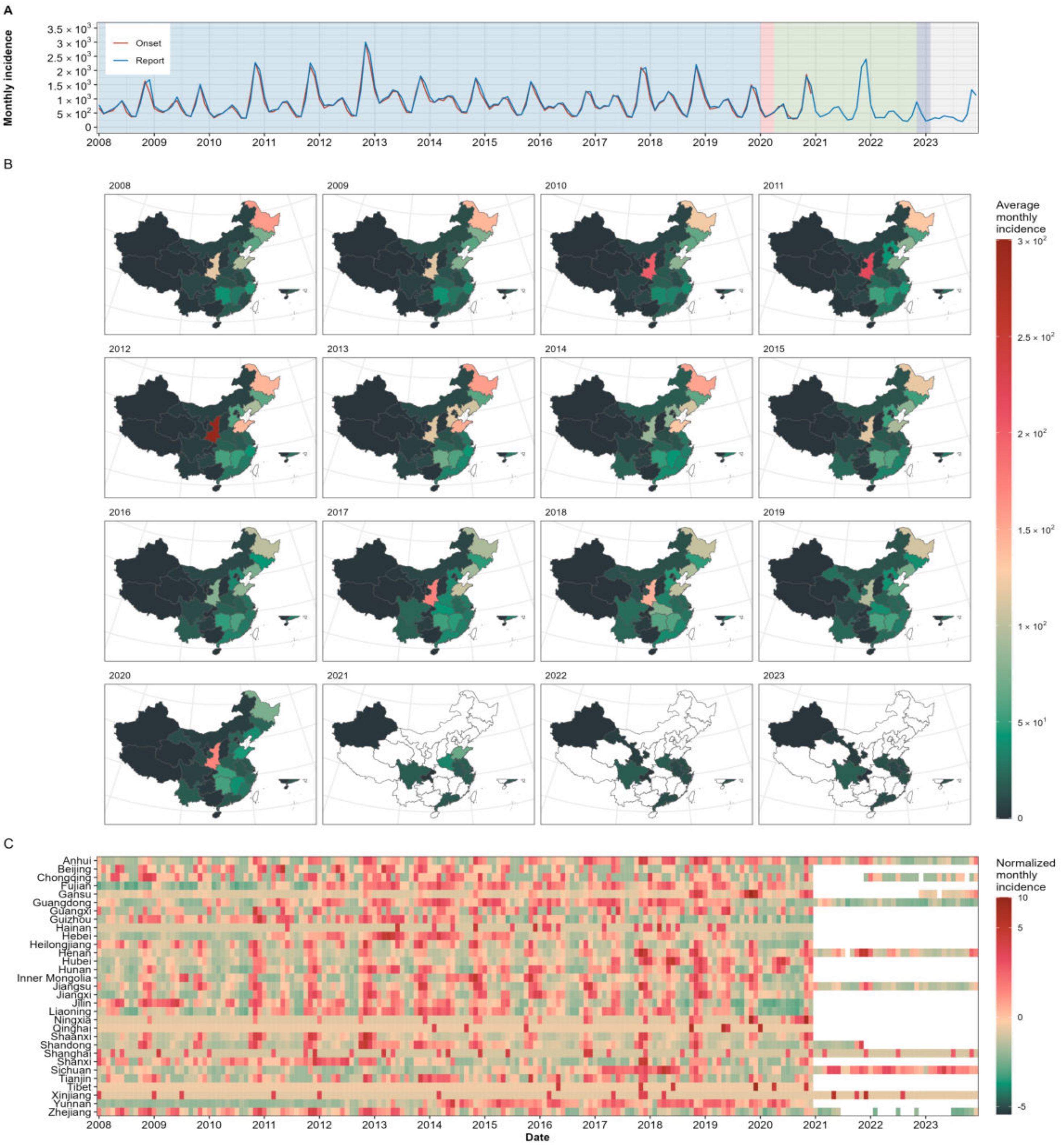

Supplementary Fig. 19. Temporal variation in monthly incidence of hemorrhagic fever with renal syndrome (HFRS) from January 2008 to December 2023 in China.

(A) The incidence of hemorrhagic fever with renal syndrome (HFRS) in China from January 2008 to December 2023; **(B)** The spatial distribution of cases in China; **(C)** Temporal variation in monthly incidence among different provinces. The heatmap represents the normalized monthly incidence data of each province, and the color intensity corresponds to the normalized monthly incidence. Provincial data in panel **(B)** and **(C)** before January 2020 sourced from the Chinese Public Health Science Data Center, and data after January 2020 sourced from the provincial Notifiable Infectious Diseases Reports. * Normalized monthly incidence > 10.

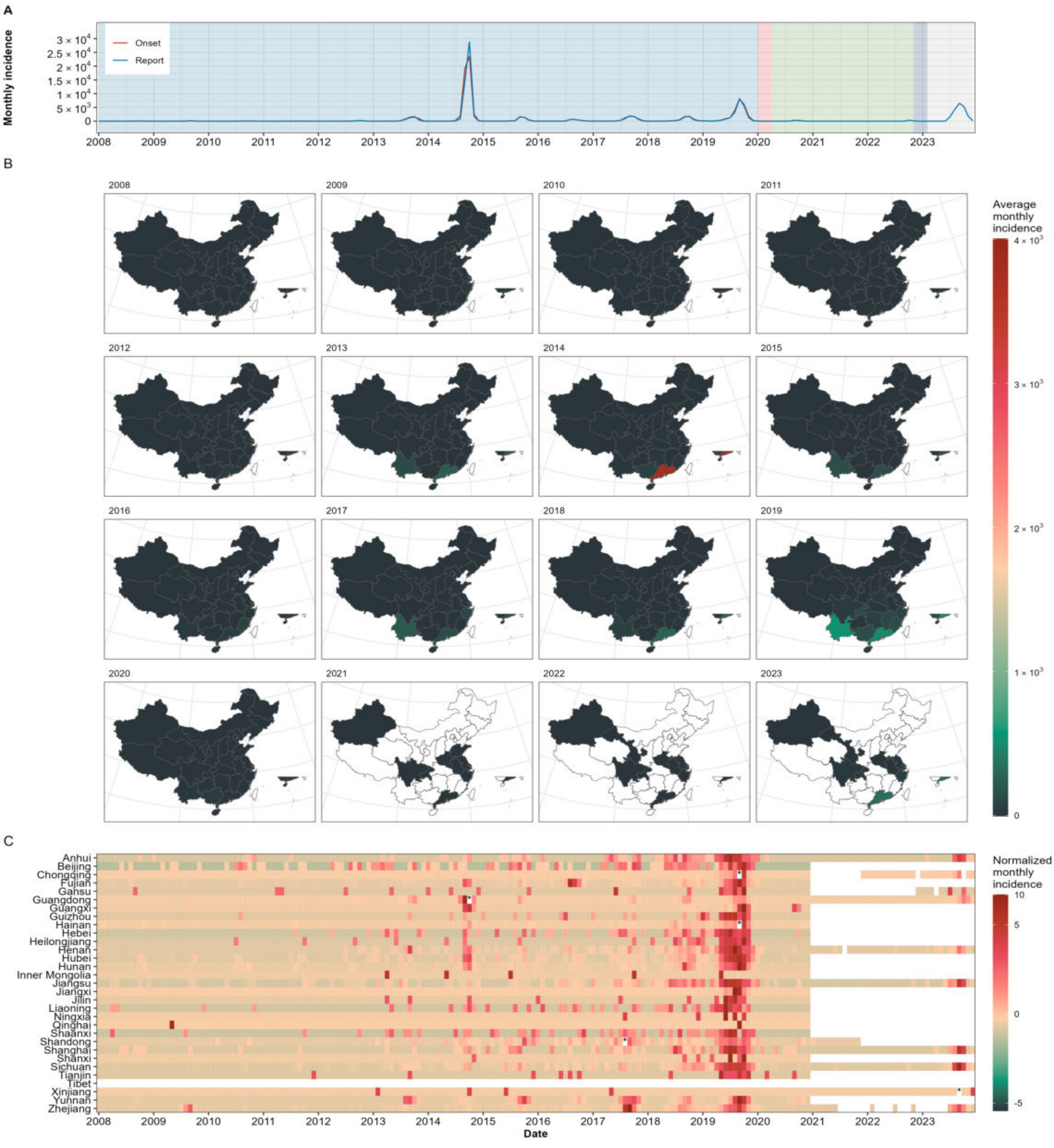

Supplementary Fig. 20. Temporal variation in monthly incidence of dengue fever from January 2008 to December 2023 in China.

(A) The incidence of dengue fever in China from January 2008 to December 2023; **(B)** The spatial distribution of cases in China; **(C)** Temporal variation in monthly incidence among different provinces. The heatmap represents the normalized monthly incidence data of each province, and the color intensity corresponds to the normalized monthly incidence. Provincial data in panel **(B)** and **(C)** before January 2020 sourced from the Chinese Public Health Science Data Center, and data after January 2020 sourced from the provincial Notifiable Infectious Diseases Reports. * Normalized monthly incidence > 10.

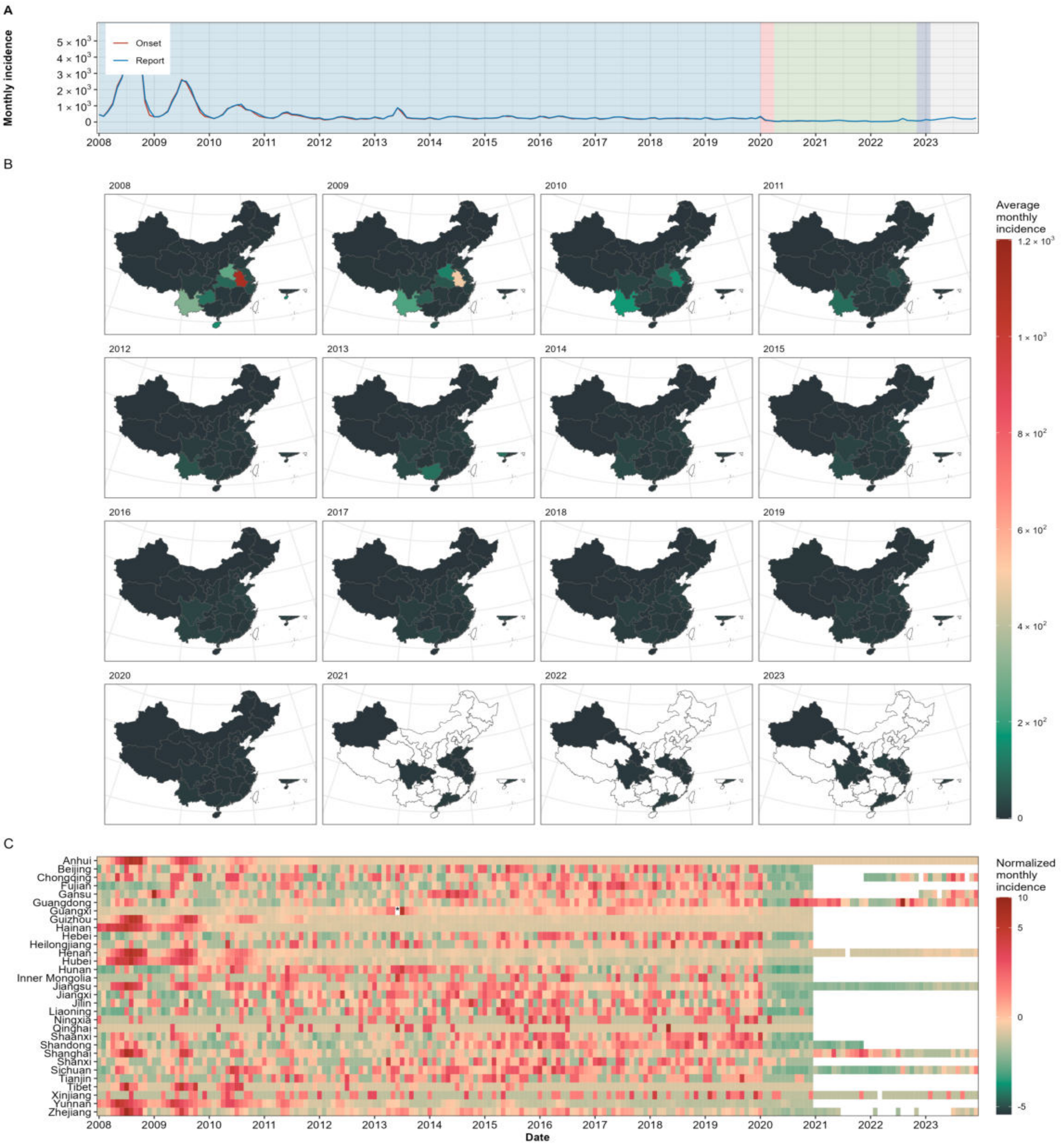

Supplementary Fig. 21. Temporal variation in monthly incidence of malaria from January 2008 to December 2023 in China.

(A) The incidence of malaria in China from January 2008 to December 2023; **(B)** The spatial distribution of cases in China; **(C)** Temporal variation in monthly incidence among different provinces. The heatmap represents the normalized monthly incidence data of each province, and the color intensity corresponds to the normalized monthly incidence. Provincial data in panel **(B)** and **(C)** before January 2020 sourced from the Chinese Public Health Science Data Center, and data after January 2020 sourced from the provincial Notifiable Infectious Diseases Reports. * Normalized monthly incidence > 10.

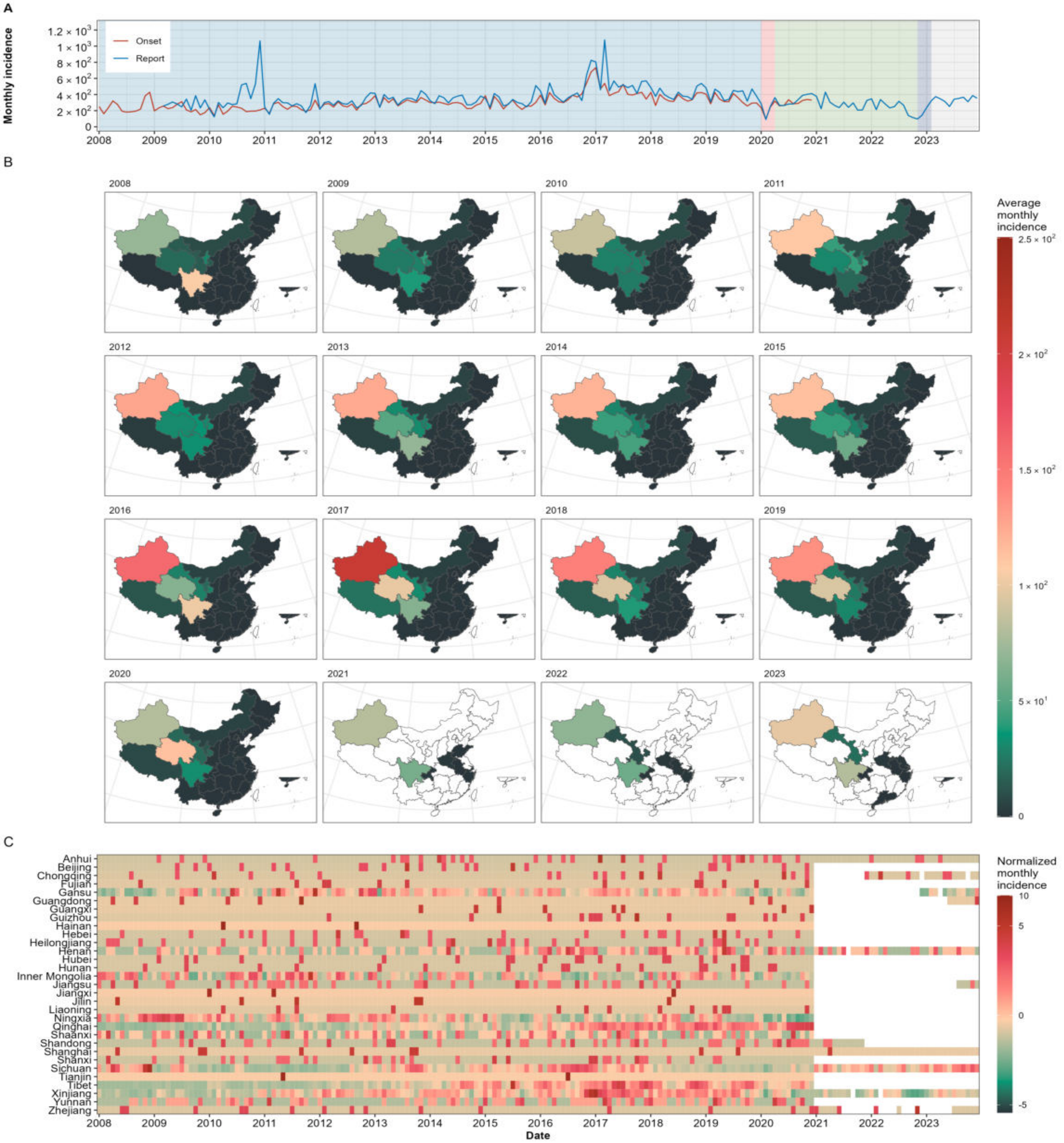

Supplementary Fig. 22. Temporal variation in monthly incidence of echinococcosis from January 2008 to December 2023 in China.

(A) The incidence of echinococcosis in China from January 2008 to December 2023; **(B)** The spatial distribution of cases in China; **(C)** Temporal variation in monthly incidence among different provinces. The heatmap represents the normalized monthly incidence data of each province, and the color intensity corresponds to the normalized monthly incidence. Provincial data in panel **(B)** and **(C)** before January 2020 sourced from the Chinese Public Health Science Data Center, and data after January 2020 sourced from the provincial Notifiable Infectious Diseases Reports. * Normalized monthly incidence > 10.

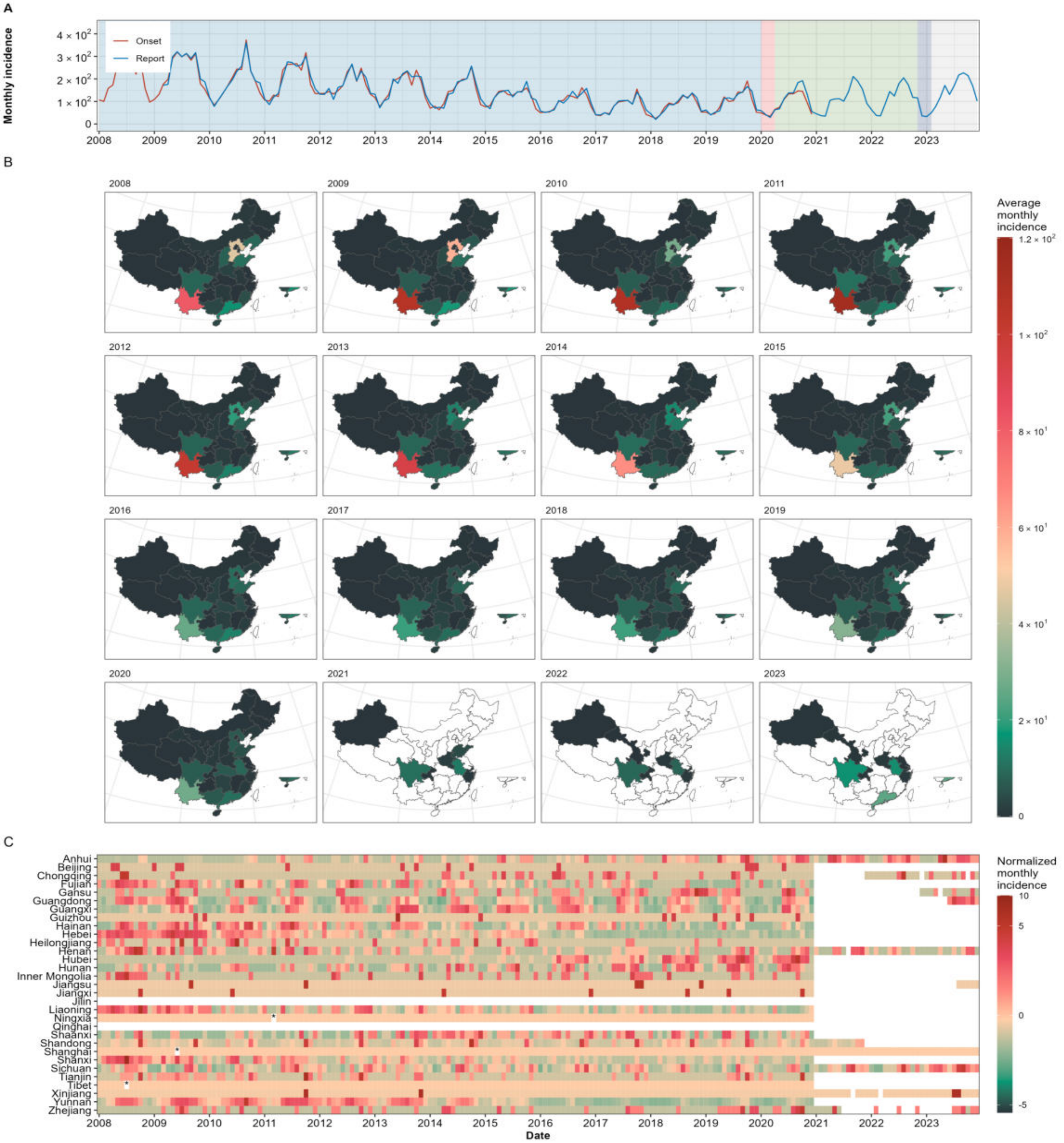

Supplementary Fig. 23. Temporal variation in monthly incidence of typhus from January 2008 to December 2023 in China.

(A) The incidence of typhus in China from January 2008 to December 2023; **(B)** The spatial distribution of cases in China; **(C)** Temporal variation in monthly incidence among different provinces. The heatmap represents the normalized monthly incidence data of each province, and the color intensity corresponds to the normalized monthly incidence. Provincial data in panel **(B)** and **(C)** before January 2020 sourced from the Chinese Public Health Science Data Center, and data after January 2020 sourced from the provincial Notifiable Infectious Diseases Reports. * Normalized monthly incidence > 10.

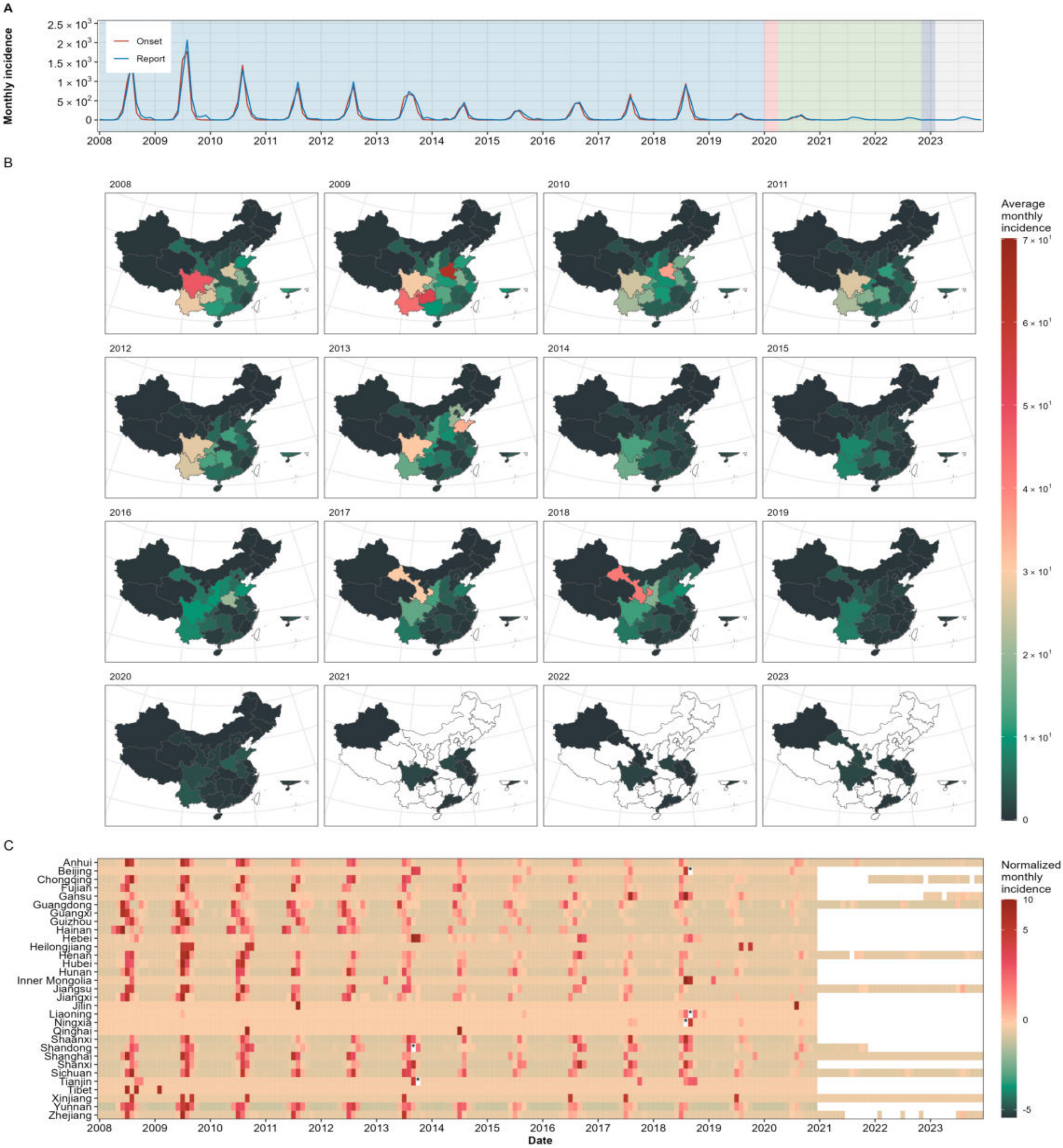

Supplementary Fig. 24. Temporal variation in monthly incidence of Japanese encephalitis (JE) from January 2008 to December 2023 in China.

(A) The incidence of Japanese encephalitis (JE) in China from January 2008 to December 2023; **(B)** The spatial distribution of cases in China; **(C)** Temporal variation in monthly incidence among different provinces. The heatmap represents the normalized monthly incidence data of each province, and the color intensity corresponds to the normalized monthly incidence. Provincial data in panel **(B)** and **(C)** before January 2020 sourced from the Chinese Public Health Science Data Center, and data after January 2020 sourced from the provincial Notifiable Infectious Diseases Reports. * Normalized monthly incidence > 10.

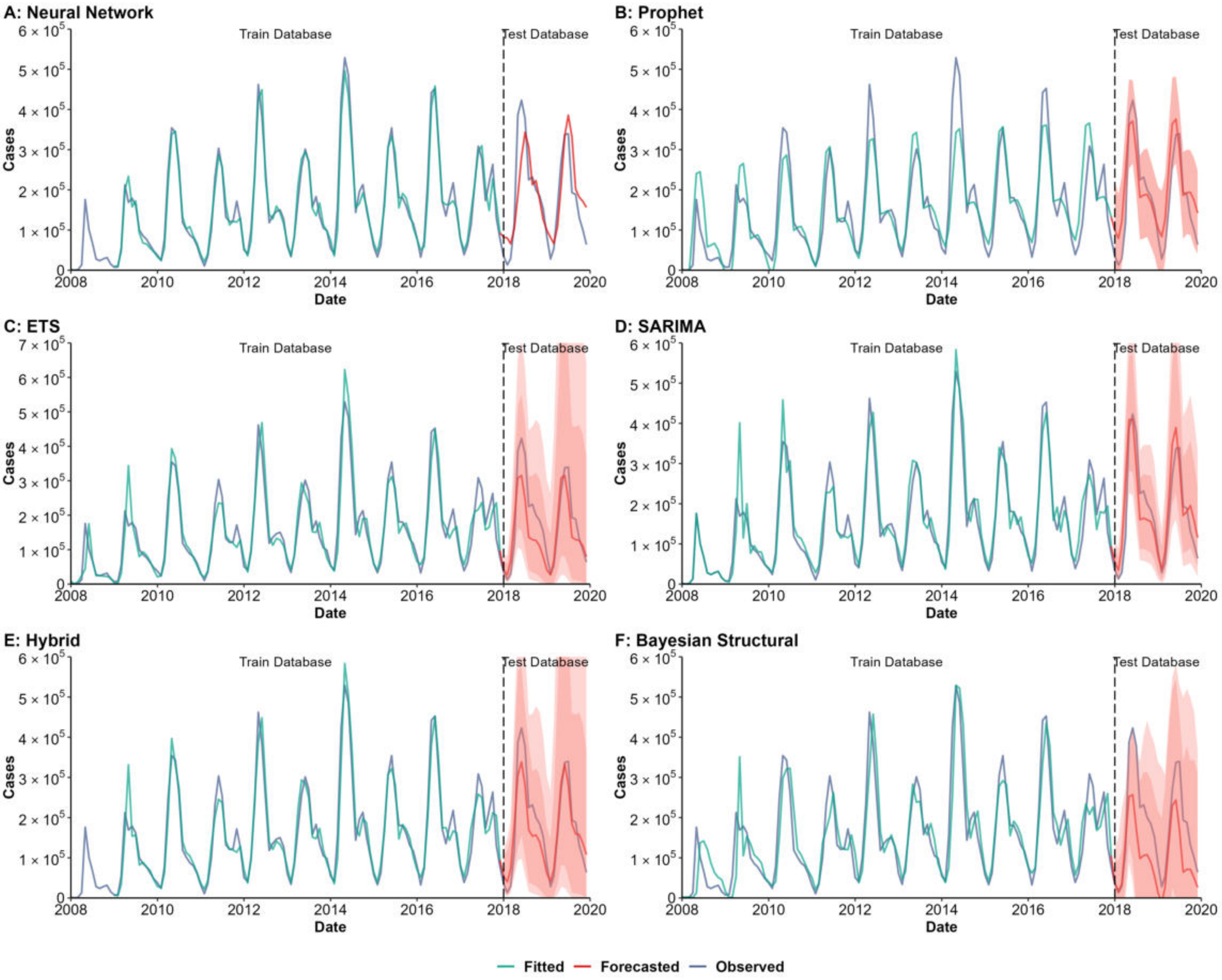

G : SMAPE of Models

Method	Train	Test	All
Neural Network	13.19	39.54	17.98
ETS	22.63	35.13	24.71
SARIMA	18.83	33.32	21.24
Hybrid*	15.83	31.71	18.71
Bayesian Structural	34.59	64.98	39.65
Prophet	37.35	42.58	38.22

*Hybrid: Combined SARIMA, ETS, STL and Neural Network model

H : RMSE of Models

Method	Train	Test	All
Neural Network	23323.90	72265.71	37344.47
ETS	40788.11	63677.84	45411.48
SARIMA	41585.44	53910.58	43880.70
Hybrid*	33188.04	48221.11	36386.27
Bayesian Structural	55537.49	98552.75	64723.40
Prophet	54513.95	62646.47	55951.52

*Hybrid: Combined SARIMA, ETS, STL and Neural Network model

I : MASE of Models

Method	Train	Test	All
Neural Network	0.30	1.10	0.43
ETS	0.47	1.07	0.56
SARIMA	0.44	0.71	0.49
Hybrid*	0.37	0.83	0.46
Bayesian Structural	0.67	1.80	0.85
Prophet	0.65	1.07	0.83

*Hybrid: Combined SARIMA, ETS, STL and Neural Network model

J : R_Squared of Models

Method	Train	Test	All
Neural Network	0.96	0.65	0.90
ETS	0.88	0.81	0.86
SARIMA	0.88	0.81	0.87
Hybrid*	0.92	0.89	0.90
Bayesian Structural	0.78	0.81	0.71
Prophet	0.78	0.79	0.77

*Hybrid: Combined SARIMA, ETS, STL and Neural Network model

Supplementary Fig. 25. Training and comparing variant time series models for hand, foot, and mouth disease (HFMD).

(A) Neural Network model; **(B)** Prophet model; **(C)** Exponential smoothing (ETS) model; **(D)** Seasonal autoregressive integrated moving average (SARIMA) model; **(E)** Hybrid models combining SARIMA, ETS, STL (seasonal and trend decomposition using loess), and neural network model; **(F)** Bayesian structural model; **(G)** Root mean square error (RMSE) of variant models; **(H)** Symmetric mean absolute percentage error (SMAPE) of variant models; **(I)** Mean absolute scaled error (MASE) of variant models; **(J)** R-squared of variant models.

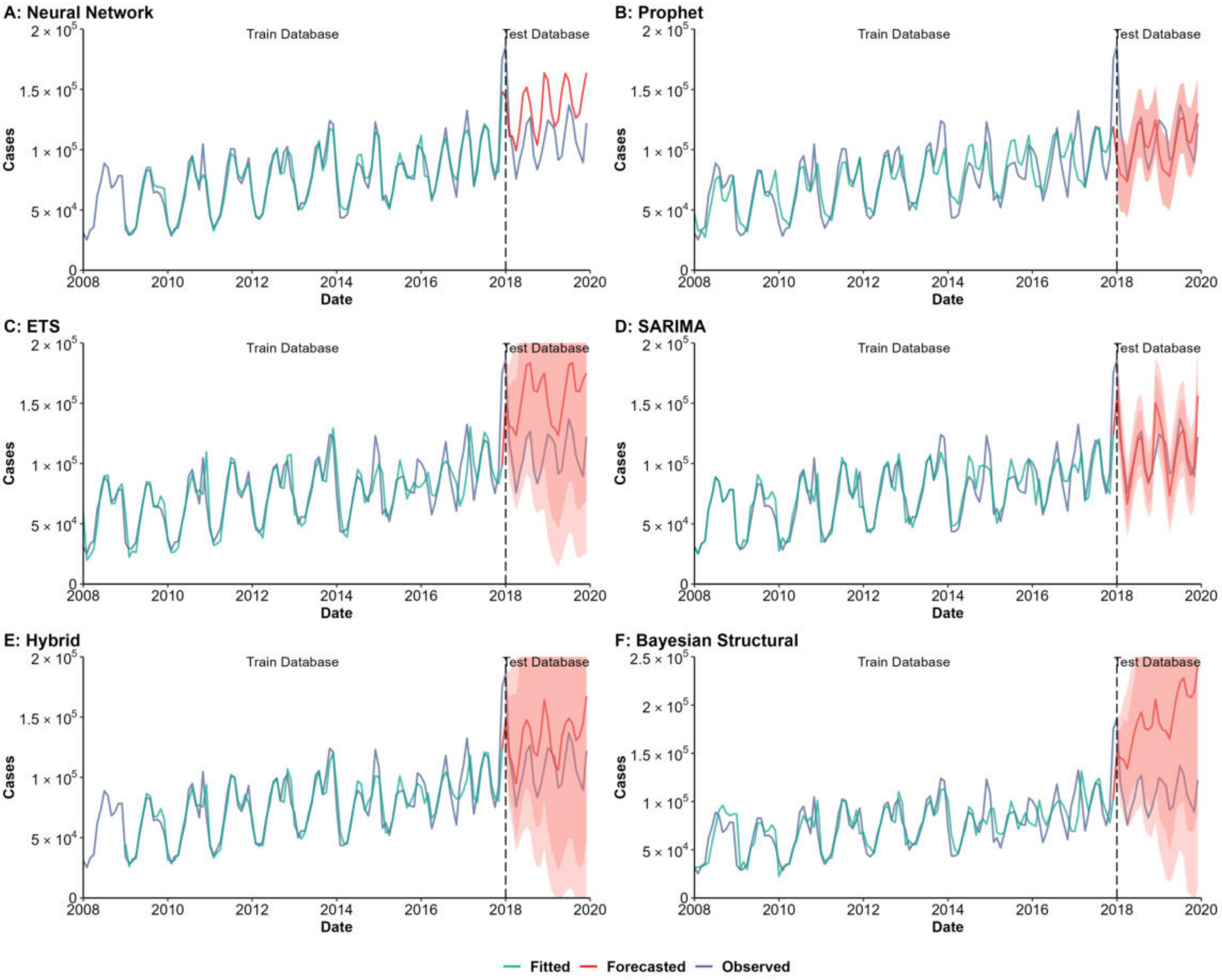

G : SMAPE of Models

Method	Train	Test	All
Neural Network	6.56	23.06	9.56
ETS	13.26	36.59	17.15
SARIMA	10.37	9.05	10.15
Hybrid*	9.41	21.78	11.66
Bayesian Structural	15.32	52.15	21.46
Prophet	15.23	15.68	15.31

*Hybrid: Combined SARIMA, ETS, STL and Neural Network model

H : RMSE of Models

Method	Train	Test	All
Neural Network	6990.88	32511.72	15237.14
ETS	14680.65	52087.60	25135.38
SARIMA	12083.49	15076.35	12631.64
Hybrid*	10773.19	30462.76	16238.32
Bayesian Structural	16986.54	80925.42	36495.74
Prophet	15073.91	26207.03	17430.44

*Hybrid: Combined SARIMA, ETS, STL and Neural Network model

I : MASE of Models

Method	Train	Test	All
Neural Network	0.39	1.76	0.69
ETS	0.66	4.02	1.17
SARIMA	0.59	0.50	0.56
Hybrid*	0.49	1.80	0.83
Bayesian Structural	0.80	6.24	1.79
Prophet	0.76	1.54	1.06

*Hybrid: Combined SARIMA, ETS, STL and Neural Network model

J : R_Squared of Models

Method	Train	Test	All
Neural Network	0.93	0.22	0.79
ETS	0.69	0.06	0.61
SARIMA	0.79	0.63	0.81
Hybrid*	0.83	0.26	0.74
Bayesian Structural	0.59	0.02	0.47
Prophet	0.67	0.05	0.63

*Hybrid: Combined SARIMA, ETS, STL and Neural Network model

Supplementary Fig. 26. Training and comparing variant time series models for infectious diarrhea.

(A) Neural Network model; **(B)** Prophet model; **(C)** Exponential smoothing (ETS) model; **(D)** Seasonal autoregressive integrated moving average (SARIMA) model; **(E)** Hybrid models combining SARIMA, ETS, STL (seasonal and trend decomposition using loess), and neural network model; **(F)** Bayesian structural model; **(G)** Root mean square error (RMSE) of variant models; **(H)** Symmetric mean absolute percentage error (SMAPE) of variant models; **(I)** Mean absolute scaled error (MASE) of variant models; **(J)** R-squared of variant models.

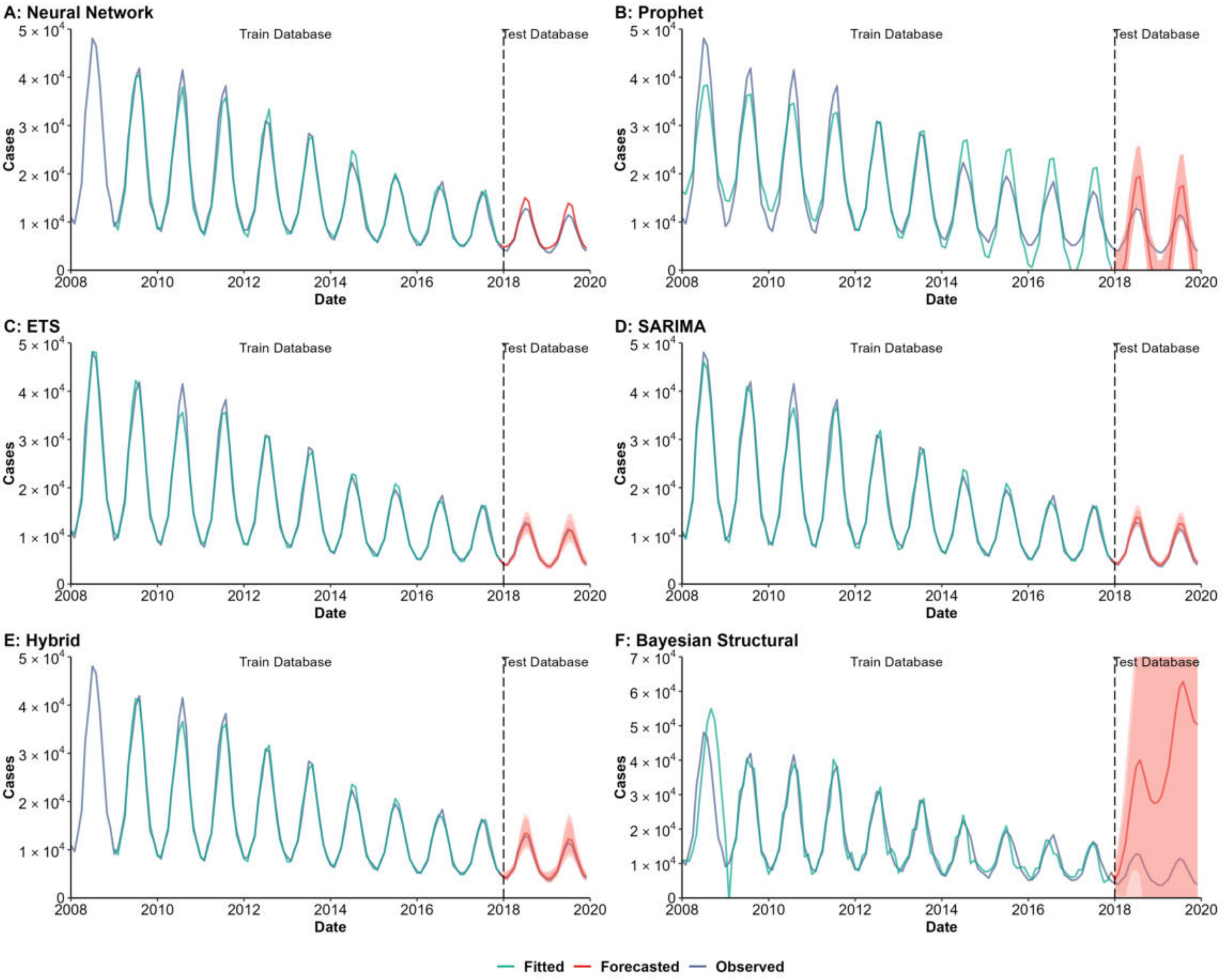

G : SMAPE of Models

Method	Train	Test	All
Neural Network	5.97	12.00	7.07
ETS	4.49	4.43	4.48
SARIMA	4.92	8.70	5.55
Hybrid*	4.52	6.38	4.86
Bayesian Structural	17.83	123.02	35.36
Prophet	25.50	108.09	39.26

*Hybrid: Combined SARIMA, ETS, STL and Neural Network model

H : RMSE of Models

Method	Train	Test	All
Neural Network	1339.95	1163.71	1309.68
ETS	1258.85	417.50	1161.74
SARIMA	1202.91	776.69	1142.97
Hybrid*	1132.16	520.12	1047.81
Bayesian Structural	4875.02	32607.31	14036.06
Prophet	3405.75	5433.71	3819.27

*Hybrid: Combined SARIMA, ETS, STL and Neural Network model

I : MASE of Models

Method	Train	Test	All
Neural Network	0.26	0.55	0.29
ETS	0.20	0.24	0.21
SARIMA	0.22	0.43	0.23
Hybrid*	0.20	0.31	0.22
Bayesian Structural	0.71	6.98	1.66
Prophet	0.68	1.26	0.79

*Hybrid: Combined SARIMA, ETS, STL and Neural Network model

J : R_Squared of Models

Method	Train	Test	All
Neural Network	0.98	0.98	0.98
ETS	0.99	0.98	0.99
SARIMA	0.99	0.99	0.99
Hybrid*	0.99	0.99	0.99
Bayesian Structural	0.81	0.20	0.21
Prophet	0.89	0.97	0.87

*Hybrid: Combined SARIMA, ETS, STL and Neural Network model

Supplementary Fig. 27. Training and comparing variant time series models for dysentery. **(A)** Neural Network model; **(B)** Prophet model; **(C)** Exponential smoothing (ETS) model; **(D)** Seasonal autoregressive integrated moving average (SARIMA) model; **(E)** Hybrid models combining SARIMA, ETS, STL (seasonal and trend decomposition using loess), and neural network model; **(F)** Bayesian structural model; **(G)** Root mean square error (RMSE) of variant models; **(H)** Symmetric mean absolute percentage error (SMAPE) of variant models; **(I)** Mean absolute scaled error (MASE) of variant models; **(J)** R-squared of variant models.

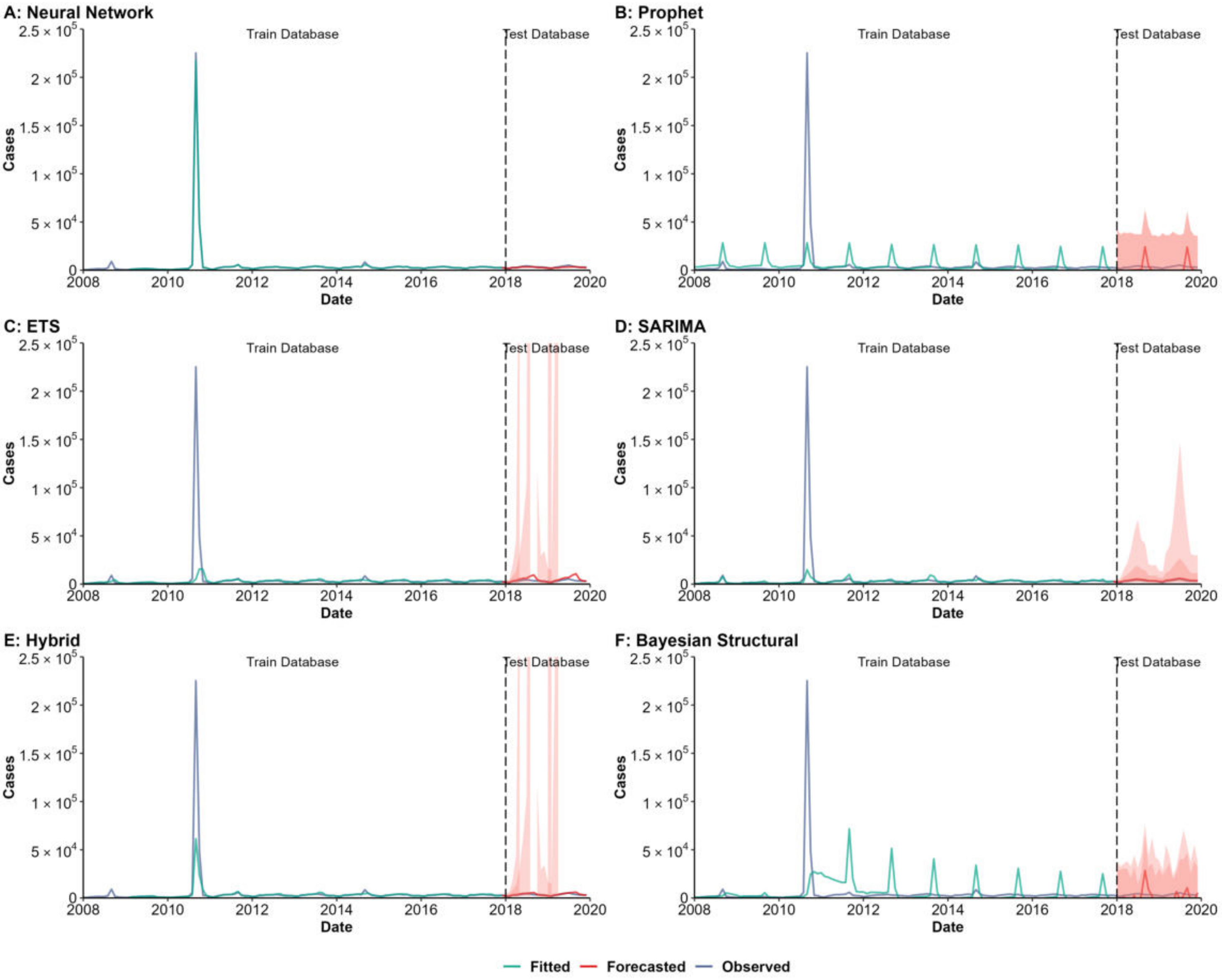

G : SMAPE of Models

Method	Train	Test	All
Neural Network	2.33	15.99	4.83
ETS	23.46	32.23	24.92
SARIMA	20.55	18.79	20.26
Hybrid*	14.04	14.44	14.11
Bayesian Structural	119.92	145.03	124.11
Prophet	87.88	175.25	102.44

*Hybrid: Combined SARIMA, ETS, STL and Neural Network model

H : RMSE of Models

Method	Train	Test	All
Neural Network	845.24	682.80	817.89
ETS	20369.45	2571.36	18624.28
SARIMA	19596.50	750.74	17891.70
Hybrid*	16051.37	837.24	14511.11
Bayesian Structural	23230.53	7742.52	21440.75
Prophet	19461.24	7003.92	17994.23

*Hybrid: Combined SARIMA, ETS, STL and Neural Network model

I : MASE of Models

Method	Train	Test	All
Neural Network	0.04	2.03	0.06
ETS	0.64	1.23	2.94
SARIMA	2.39	1.38	2.31
Hybrid*	0.46	0.92	1.30
Bayesian Structural	1.82	0.81	1.37
Prophet	1.26	1.13	1.25

*Hybrid: Combined SARIMA, ETS, STL and Neural Network model

J : R_Squared of Models

Method	Train	Test	All
Neural Network	1.00	0.70	1.00
ETS	0.06	0.48	0.04
SARIMA	0.37	0.91	0.34
Hybrid*	0.95	0.67	0.94
Bayesian Structural	0.00	0.02	0.00
Prophet	0.11	0.01	0.10

*Hybrid: Combined SARIMA, ETS, STL and Neural Network model

Supplementary Fig. 28. Training and comparing variant time series models for acute hemorrhagic conjunctivitis (AHC).

(A) Neural Network model; **(B)** Prophet model; **(C)** Exponential smoothing (ETS) model; **(D)** Seasonal autoregressive integrated moving average (SARIMA) model; **(E)** Hybrid models combining SARIMA, ETS, STL (seasonal and trend decomposition using loess), and neural network model; **(F)** Bayesian structural model; **(G)** Root mean square error (RMSE) of variant models; **(H)** Symmetric mean absolute percentage error (SMAPE) of variant models; **(I)** Mean absolute scaled error (MASE) of variant models; **(J)** R-squared of variant models.

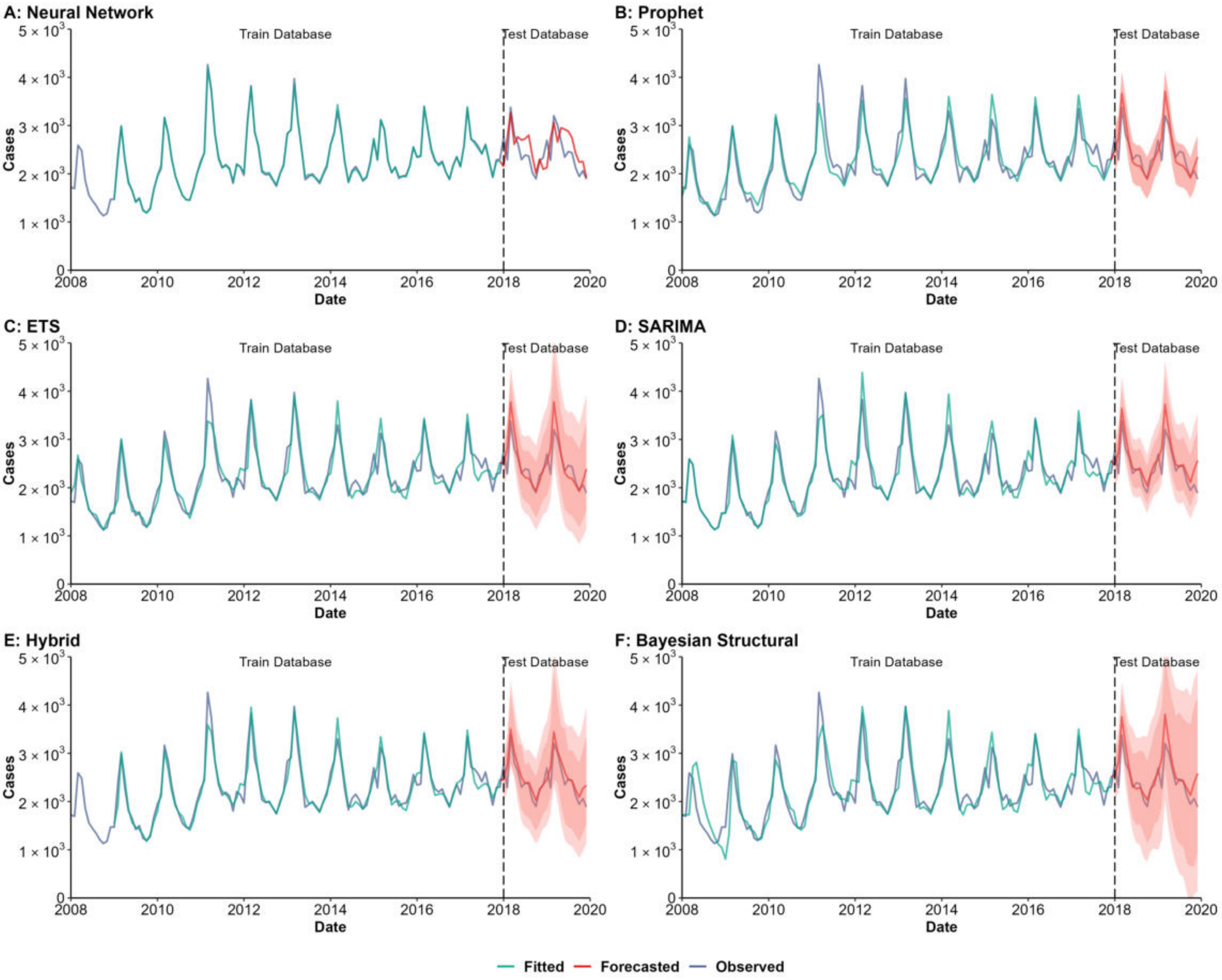

G : SMAPE of Models

Method	Train	Test	All
Neural Network	0.99	12.28	3.04
ETS	6.96	7.91	7.12
SARIMA	6.43	8.26	6.73
Hybrid*	5.13	7.15	5.49
Bayesian Structural	10.03	8.68	9.81
Prophet	7.37	7.85	7.45

*Hybrid: Combined SARIMA, ETS, STL and Neural Network model

H : RMSE of Models

Method	Train	Test	All
Neural Network	31.67	346.22	150.38
ETS	222.24	275.29	231.92
SARIMA	230.76	273.79	238.47
Hybrid*	168.61	222.28	179.57
Bayesian Structural	308.89	301.30	307.64
Prophet	222.36	254.26	227.99

*Hybrid: Combined SARIMA, ETS, STL and Neural Network model

I : MASE of Models

Method	Train	Test	All
Neural Network	0.07	1.15	0.23
ETS	0.48	0.65	0.55
SARIMA	0.47	0.71	0.51
Hybrid*	0.36	0.72	0.44
Bayesian Structural	0.66	0.75	0.70
Prophet	0.51	0.66	0.58

*Hybrid: Combined SARIMA, ETS, STL and Neural Network model

J : R_Squared of Models

Method	Train	Test	All
Neural Network	1.00	0.38	0.93
ETS	0.86	0.75	0.84
SARIMA	0.86	0.74	0.85
Hybrid*	0.91	0.78	0.89
Bayesian Structural	0.76	0.72	0.75
Prophet	0.86	0.75	0.84

*Hybrid: Combined SARIMA, ETS, STL and Neural Network model

Supplementary Fig. 29. Training and comparing variant time series models for hepatitis E. **(A)** Neural Network model; **(B)** Prophet model; **(C)** Exponential smoothing (ETS) model; **(D)** Seasonal autoregressive integrated moving average (SARIMA) model; **(E)** Hybrid models combining SARIMA, ETS, STL (seasonal and trend decomposition using loess), and neural network model; **(F)** Bayesian structural model; **(G)** Root mean square error (RMSE) of variant models; **(H)** Symmetric mean absolute percentage error (SMAPE) of variant models; **(I)** Mean absolute scaled error (MASE) of variant models; **(J)** R-squared of variant models.

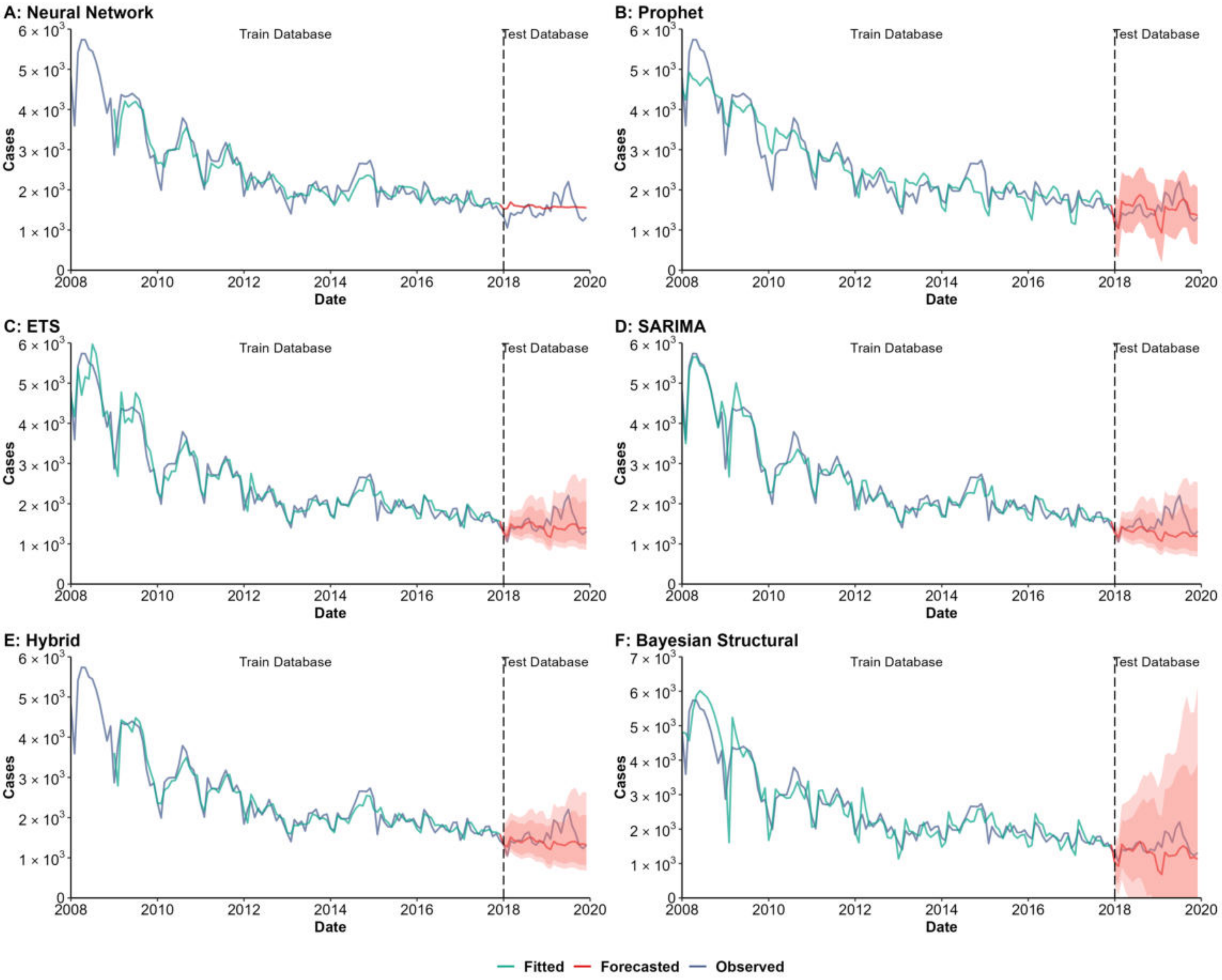

G : SMAPE of Models

Method	Train	Test	All
Neural Network	8.38	14.48	9.49
ETS	6.94	13.53	8.04
SARIMA	6.80	18.57	8.76
Hybrid*	6.46	14.06	7.85
Bayesian Structural	10.83	20.47	12.44
Prophet	10.68	15.57	11.50

*Hybrid: Combined SARIMA, ETS, STL and Neural Network model

H : RMSE of Models

Method	Train	Test	All
Neural Network	263.42	271.41	264.89
ETS	264.65	293.40	269.65
SARIMA	252.16	388.66	279.58
Hybrid*	209.55	316.97	232.80
Bayesian Structural	408.83	398.95	407.20
Prophet	360.64	288.37	349.64

*Hybrid: Combined SARIMA, ETS, STL and Neural Network model

I : MASE of Models

Method	Train	Test	All
Neural Network	1.29	8.67	1.56
ETS	0.71	2.74	0.86
SARIMA	0.73	3.81	0.91
Hybrid*	0.65	3.48	1.03
Bayesian Structural	1.05	1.78	0.88
Prophet	1.02	1.50	1.47

*Hybrid: Combined SARIMA, ETS, STL and Neural Network model

J : R_Squared of Models

Method	Train	Test	All
Neural Network	0.87	0.00	0.87
ETS	0.94	0.06	0.94
SARIMA	0.94	0.00	0.94
Hybrid*	0.92	0.00	0.91
Bayesian Structural	0.88	0.06	0.89
Prophet	0.88	0.13	0.89

*Hybrid: Combined SARIMA, ETS, STL and Neural Network model

Supplementary Fig. 30. Training and comparing variant time series models for hepatitis A. **(A)** Neural Network model; **(B)** Prophet model; **(C)** Exponential smoothing (ETS) model; **(D)** Seasonal autoregressive integrated moving average (SARIMA) model; **(E)** Hybrid models combining SARIMA, ETS, STL (seasonal and trend decomposition using loess), and neural network model; **(F)** Bayesian structural model; **(G)** Root mean square error (RMSE) of variant models; **(H)** Symmetric mean absolute percentage error (SMAPE) of variant models; **(I)** Mean absolute scaled error (MASE) of variant models; **(J)** R-squared of variant models.

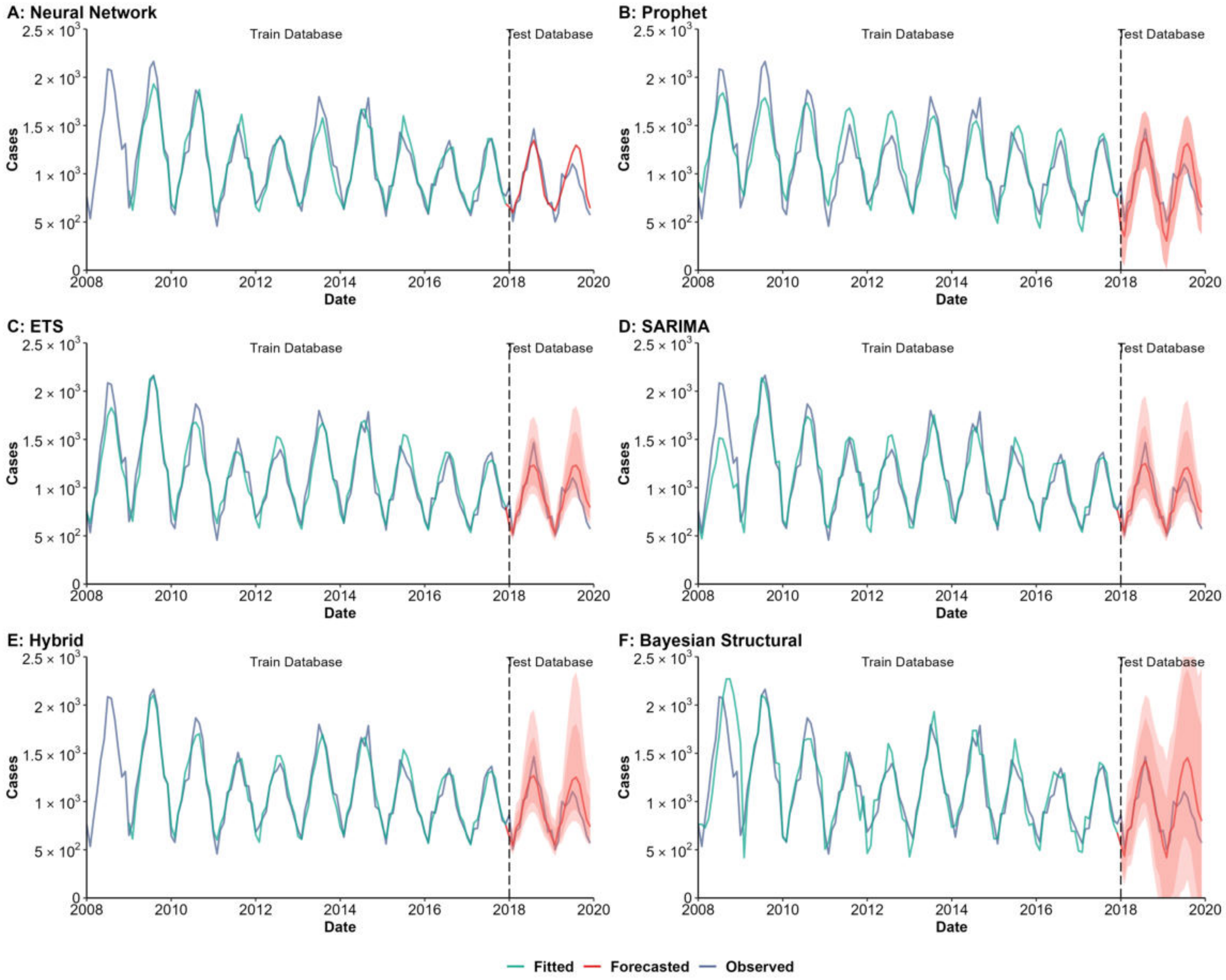

G : SMAPE of Models

Method	Train	Test	All
Neural Network	8.48	12.72	9.25
ETS	8.21	13.65	9.12
SARIMA	9.43	12.75	9.98
Hybrid*	6.86	13.23	8.02
Bayesian Structural	12.74	17.62	13.56
Prophet	12.04	18.88	13.18

*Hybrid: Combined SARIMA, ETS, STL and Neural Network model

H : RMSE of Models

Method	Train	Test	All
Neural Network	118.27	142.29	122.99
ETS	114.56	146.43	120.45
SARIMA	148.46	131.32	145.75
Hybrid*	91.41	138.18	101.53
Bayesian Structural	205.33	203.74	205.07
Prophet	157.24	180.16	161.29

*Hybrid: Combined SARIMA, ETS, STL and Neural Network model

I : MASE of Models

Method	Train	Test	All
Neural Network	0.57	0.90	0.62
ETS	0.48	1.05	0.59
SARIMA	0.61	0.96	0.65
Hybrid*	0.42	0.97	0.52
Bayesian Structural	0.74	0.96	0.72
Prophet	0.71	0.85	0.77

*Hybrid: Combined SARIMA, ETS, STL and Neural Network model

J : R_Squared of Models

Method	Train	Test	All
Neural Network	0.90	0.72	0.88
ETS	0.91	0.67	0.90
SARIMA	0.86	0.73	0.86
Hybrid*	0.94	0.71	0.92
Bayesian Structural	0.78	0.65	0.77
Prophet	0.83	0.71	0.82

*Hybrid: Combined SARIMA, ETS, STL and Neural Network model

Supplementary Fig. 31. Training and comparing variant time series models for enteric fever.

(A) Neural Network model; **(B)** Prophet model; **(C)** Exponential smoothing (ETS) model; **(D)** Seasonal autoregressive integrated moving average (SARIMA) model; **(E)** Hybrid models combining SARIMA, ETS, STL (seasonal and trend decomposition using loess), and neural network model; **(F)** Bayesian structural model; **(G)** Root mean square error (RMSE) of variant models; **(H)** Symmetric mean absolute percentage error (SMAPE) of variant models; **(I)** Mean absolute scaled error (MASE) of variant models; **(J)** R-squared of variant models.

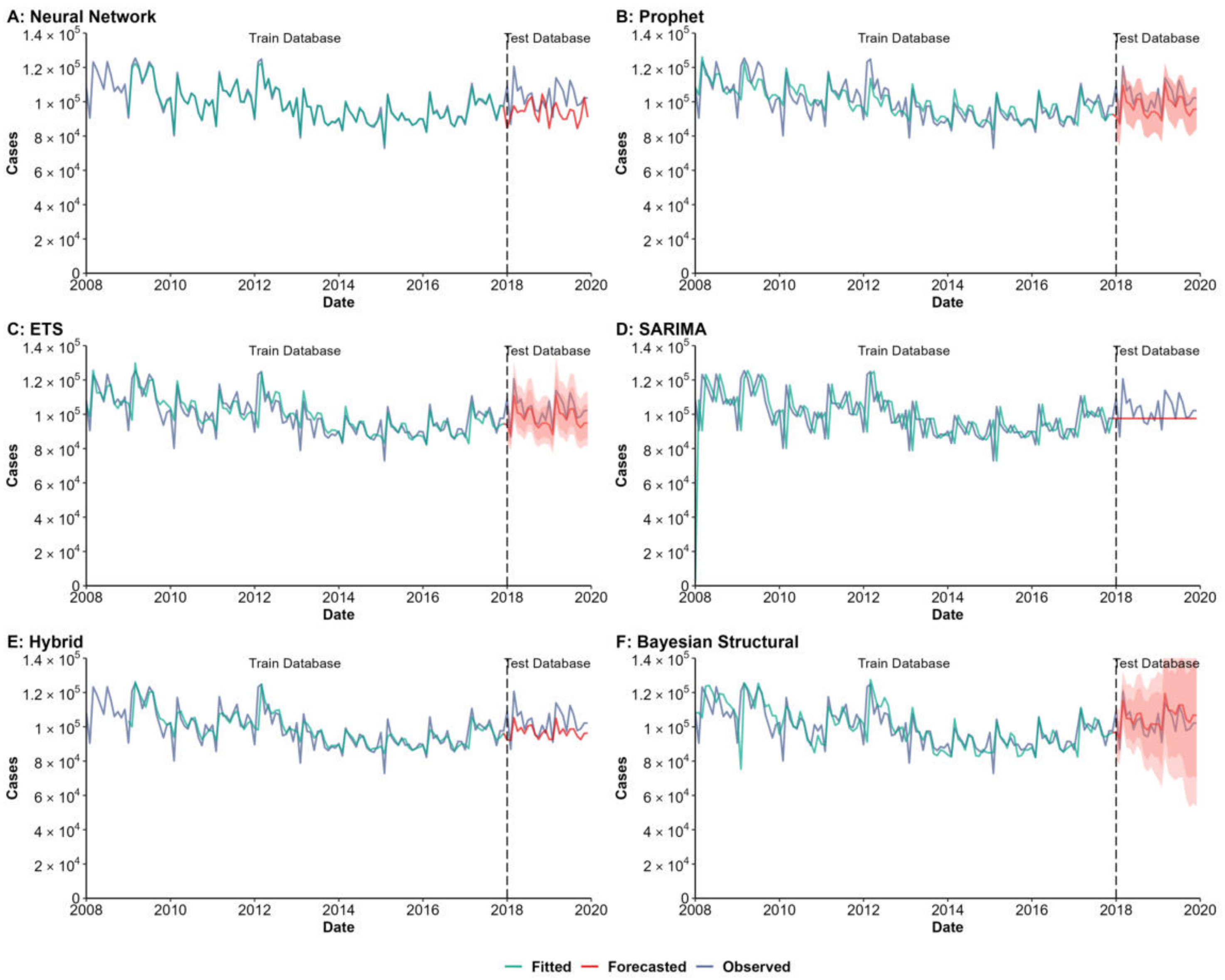

G : SMAPE of Models

Method	Train	Test	All
Neural Network	0.71	10.58	2.50
ETS	4.23	5.29	4.41
SARIMA	9.86	7.33	9.44
Hybrid*	3.76	6.81	4.31
Bayesian Structural	5.35	4.52	5.22
Prophet	4.53	5.88	4.76

*Hybrid: Combined SARIMA, ETS, STL and Neural Network model

H : RMSE of Models

Method	Train	Test	All
Neural Network	1041.08	12656.05	5478.10
ETS	6186.51	6740.76	6282.28
SARIMA	15019.03	9396.49	14236.98
Hybrid*	5523.44	8479.68	6167.26
Bayesian Structural	8381.15	5365.91	7958.34
Prophet	6389.18	7366.71	6562.22

*Hybrid: Combined SARIMA, ETS, STL and Neural Network model

I : MASE of Models

Method	Train	Test	All
Neural Network	0.10	1.62	0.35
ETS	0.51	0.98	0.78
SARIMA	0.99	Inf	1.16
Hybrid*	0.46	1.72	1.02
Bayesian Structural	0.65	0.91	0.83
Prophet	0.55	1.11	0.88

*Hybrid: Combined SARIMA, ETS, STL and Neural Network model

J : R_Squared of Models

Method	Train	Test	All
Neural Network	0.99	0.02	0.75
ETS	0.70	0.71	0.67
SARIMA	0.10		0.09
Hybrid*	0.73	0.48	0.65
Bayesian Structural	0.54	0.58	0.55
Prophet	0.67	0.67	0.64

*Hybrid: Combined SARIMA, ETS, STL and Neural Network model

Supplementary Fig. 32. Training and comparing variant time series models for hepatitis B. (A) Neural Network model; **(B)** Prophet model; **(C)** Exponential smoothing (ETS) model; **(D)** Seasonal autoregressive integrated moving average (SARIMA) model; **(E)** Hybrid models combining SARIMA, ETS, STL (seasonal and trend decomposition using loess), and neural network model; **(F)** Bayesian structural model; **(G)** Root mean square error (RMSE) of variant models; **(H)** Symmetric mean absolute percentage error (SMAPE) of variant models; **(I)** Mean absolute scaled error (MASE) of variant models; **(J)** R-squared of variant models.

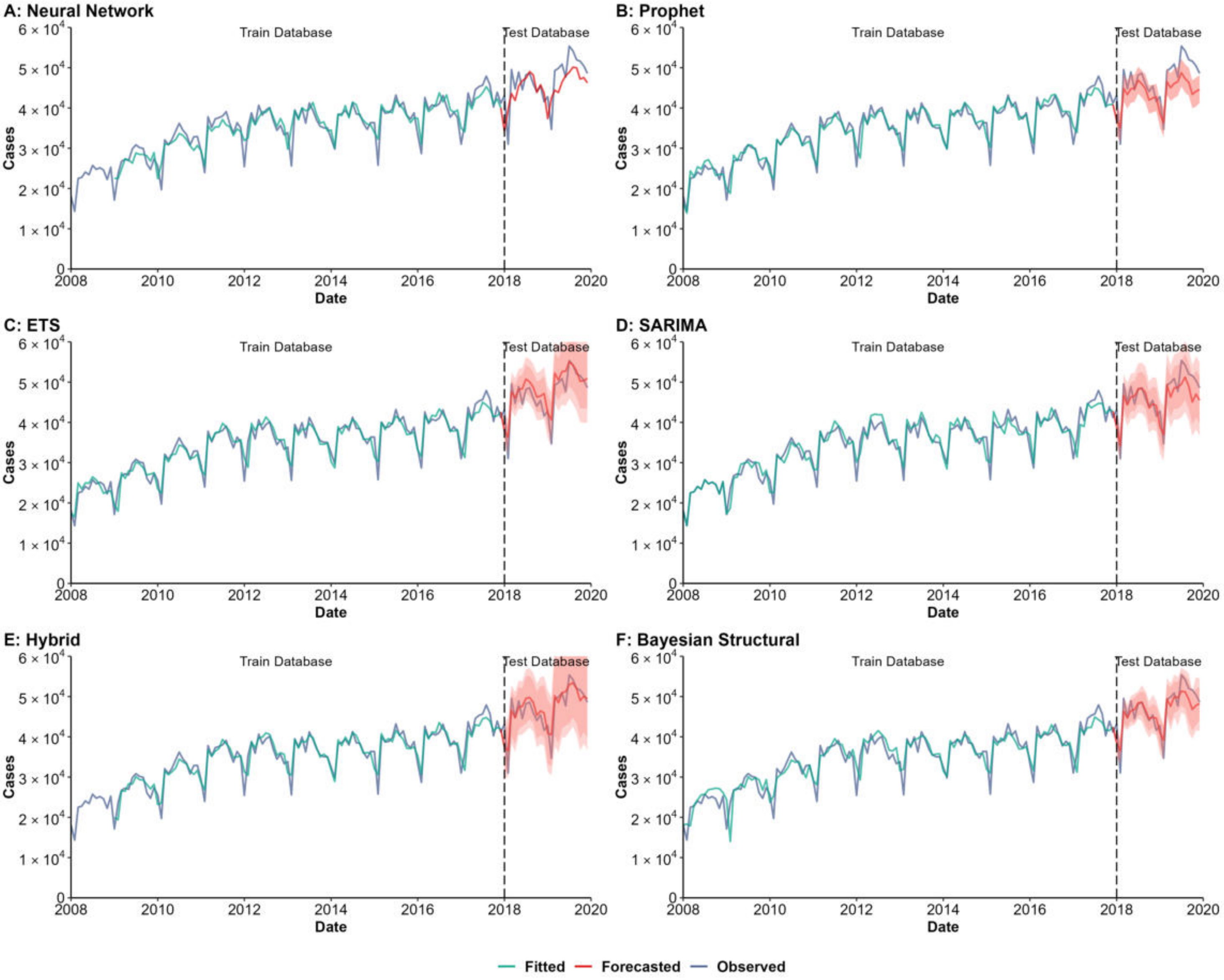

G : SMAPE of Models

Method	Train	Test	All
Neural Network	5.13	8.48	5.74
ETS	4.45	5.26	4.59
SARIMA	4.30	5.40	4.48
Hybrid*	4.18	4.90	4.31
Bayesian Structural	5.85	4.86	5.68
Prophet	4.08	8.16	4.76

*Hybrid: Combined SARIMA, ETS, STL and Neural Network model

H : RMSE of Models

Method	Train	Test	All
Neural Network	2280.48	4523.55	2824.09
ETS	1865.68	2960.59	2088.41
SARIMA	1943.45	2973.30	2149.63
Hybrid*	1821.54	2743.41	2020.68
Bayesian Structural	2469.89	2717.47	2512.84
Prophet	1755.91	4295.93	2375.96

*Hybrid: Combined SARIMA, ETS, STL and Neural Network model

I : MASE of Models

Method	Train	Test	All
Neural Network	0.79	1.65	0.94
ETS	0.45	0.98	0.70
SARIMA	0.57	0.84	0.62
Hybrid*	0.44	0.94	0.69
Bayesian Structural	0.58	0.99	0.87
Prophet	0.42	1.68	0.75

*Hybrid: Combined SARIMA, ETS, STL and Neural Network model

J : R_Squared of Models

Method	Train	Test	All
Neural Network	0.84	0.42	0.85
ETS	0.93	0.78	0.93
SARIMA	0.92	0.78	0.93
Hybrid*	0.90	0.76	0.92
Bayesian Structural	0.87	0.80	0.90
Prophet	0.93	0.76	0.93

*Hybrid: Combined SARIMA, ETS, STL and Neural Network model

Supplementary Fig. 33. Training and comparing variant time series models for syphilis. **(A)** Neural Network model; **(B)** Prophet model; **(C)** Exponential smoothing (ETS) model; **(D)** Seasonal autoregressive integrated moving average (SARIMA) model; **(E)** Hybrid models combining SARIMA, ETS, STL (seasonal and trend decomposition using loess), and neural network model; **(F)** Bayesian structural model; **(G)** Root mean square error (RMSE) of variant models; **(H)** Symmetric mean absolute percentage error (SMAPE) of variant models; **(I)** Mean absolute scaled error (MASE) of variant models; **(J)** R-squared of variant models.

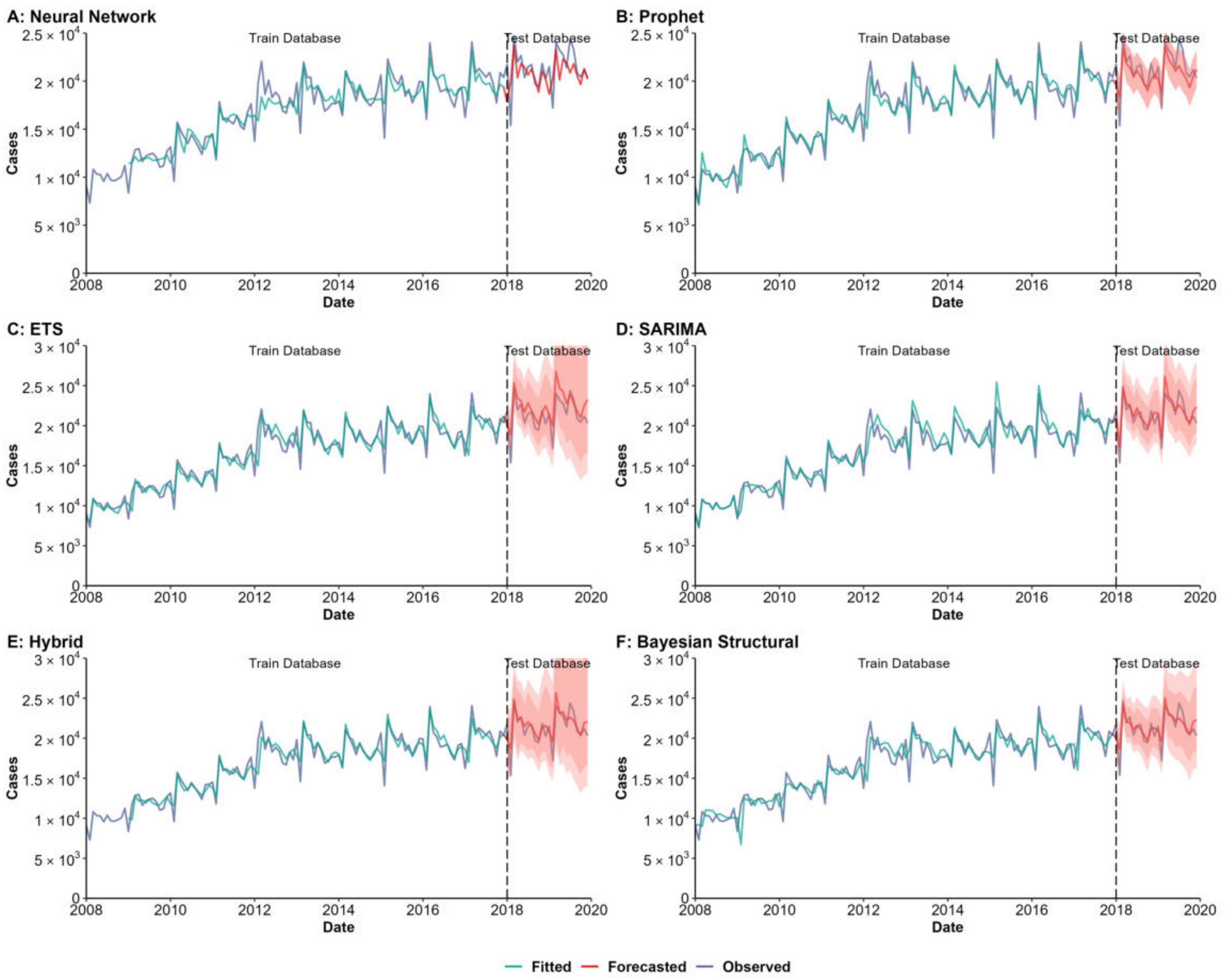

G : SMAPE of Models

Method	Train	Test	All
Neural Network	5.75	6.82	5.95
ETS	4.87	6.22	5.09
SARIMA	4.61	4.11	4.53
Hybrid*	4.41	4.63	4.45
Bayesian Structural	6.26	4.42	5.96
Prophet	4.50	5.35	4.64

*Hybrid: Combined SARIMA, ETS, STL and Neural Network model

H : RMSE of Models

Method	Train	Test	All
Neural Network	1313.61	1921.85	1443.39
ETS	1062.89	1602.86	1170.31
SARIMA	1076.37	1059.44	1073.56
Hybrid*	1026.21	1245.38	1069.41
Bayesian Structural	1336.63	1208.47	1316.14
Prophet	1008.31	1367.16	1076.46

*Hybrid: Combined SARIMA, ETS, STL and Neural Network model

I : MASE of Models

Method	Train	Test	All
Neural Network	0.99	0.87	0.96
ETS	0.48	0.82	0.68
SARIMA	0.61	0.49	0.59
Hybrid*	0.43	0.68	0.66
Bayesian Structural	0.59	0.73	0.94
Prophet	0.44	0.92	0.66

*Hybrid: Combined SARIMA, ETS, STL and Neural Network model

J : R_Squared of Models

Method	Train	Test	All
Neural Network	0.83	0.24	0.82
ETS	0.92	0.67	0.92
SARIMA	0.93	0.77	0.94
Hybrid*	0.90	0.68	0.90
Bayesian Structural	0.88	0.70	0.89
Prophet	0.93	0.69	0.93

*Hybrid: Combined SARIMA, ETS, STL and Neural Network model

Supplementary Fig. 34. Training and comparing variant time series models for hepatitis C. **(A)** Neural Network model; **(B)** Prophet model; **(C)** Exponential smoothing (ETS) model; **(D)** Seasonal autoregressive integrated moving average (SARIMA) model; **(E)** Hybrid models combining SARIMA, ETS, STL (seasonal and trend decomposition using loess), and neural network model; **(F)** Bayesian structural model; **(G)** Root mean square error (RMSE) of variant models; **(H)** Symmetric mean absolute percentage error (SMAPE) of variant models; **(I)** Mean absolute scaled error (MASE) of variant models; **(J)** R-squared of variant models.

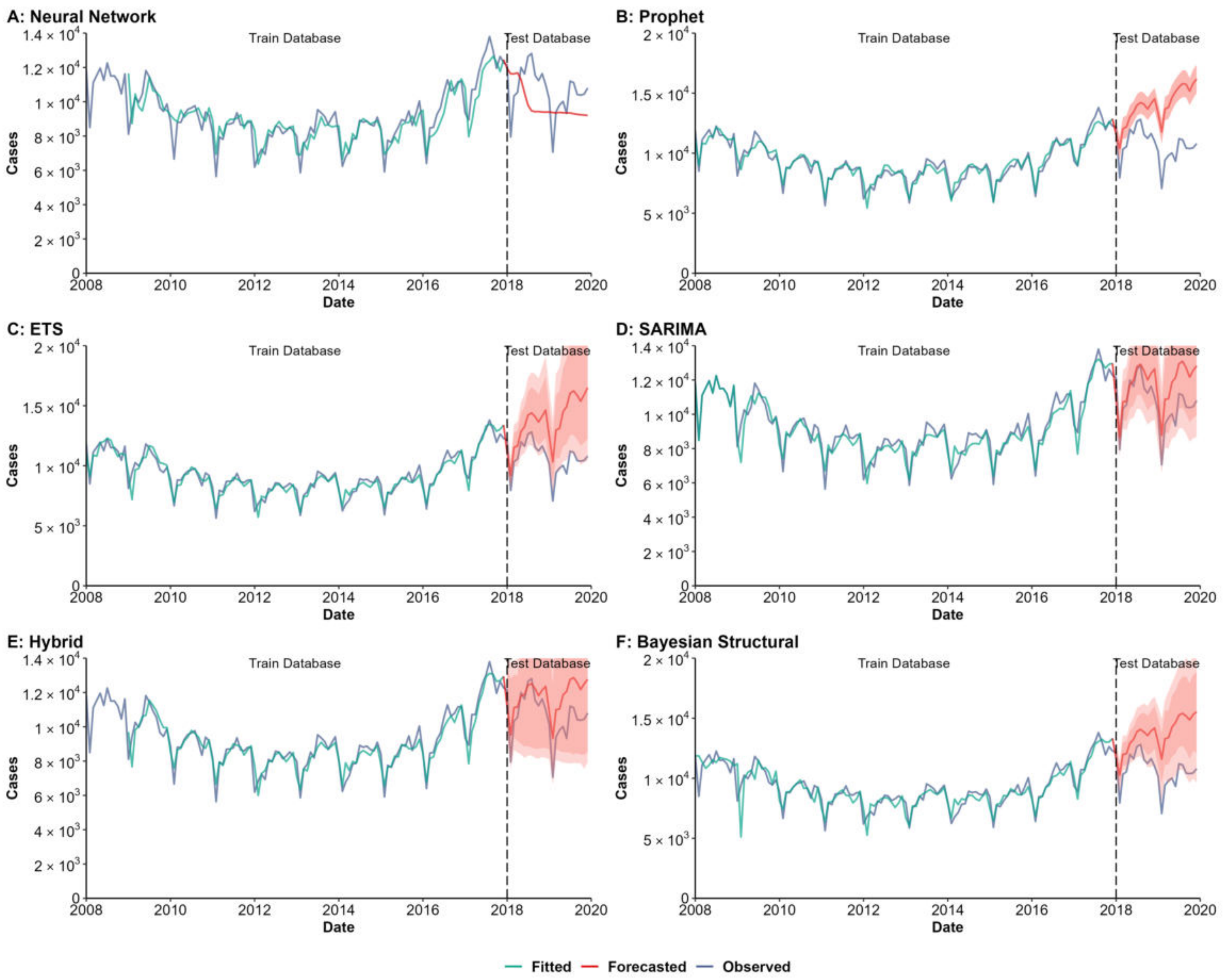

G : SMAPE of Models

Method	Train	Test	All
Neural Network	6.22	14.29	7.68
ETS	4.49	25.52	8.00
SARIMA	4.50	11.09	5.60
Hybrid*	4.29	11.46	5.59
Bayesian Structural	5.78	25.16	9.01
Prophet	4.06	27.09	7.90

*Hybrid: Combined SARIMA, ETS, STL and Neural Network model

H : RMSE of Models

Method	Train	Test	All
Neural Network	768.47	1741.24	1017.07
ETS	527.64	3552.64	1528.25
SARIMA	531.86	1435.89	761.16
Hybrid*	512.80	1436.89	768.47
Bayesian Structural	803.90	3352.88	1553.12
Prophet	472.92	3665.31	1557.39

*Hybrid: Combined SARIMA, ETS, STL and Neural Network model

I : MASE of Models

Method	Train	Test	All
Neural Network	0.92	11.29	1.38
ETS	0.52	3.43	1.25
SARIMA	0.58	1.58	0.76
Hybrid*	0.52	2.07	0.90
Bayesian Structural	0.67	4.99	1.37
Prophet	0.47	5.13	1.37

*Hybrid: Combined SARIMA, ETS, STL and Neural Network model

J : R_Squared of Models

Method	Train	Test	All
Neural Network	0.76	0.00	0.63
ETS	0.90	0.17	0.64
SARIMA	0.91	0.52	0.83
Hybrid*	0.90	0.51	0.82
Bayesian Structural	0.78	0.13	0.59
Prophet	0.92	0.09	0.60

*Hybrid: Combined SARIMA, ETS, STL and Neural Network model

Supplementary Fig. 35. Training and comparing variant time series models for gonorrhea. **(A)** Neural Network model; **(B)** Prophet model; **(C)** Exponential smoothing (ETS) model; **(D)** Seasonal autoregressive integrated moving average (SARIMA) model; **(E)** Hybrid models combining SARIMA, ETS, STL (seasonal and trend decomposition using loess), and neural network model; **(F)** Bayesian structural model; **(G)** Root mean square error (RMSE) of variant models; **(H)** Symmetric mean absolute percentage error (SMAPE) of variant models; **(I)** Mean absolute scaled error (MASE) of variant models; **(J)** R-squared of variant models.

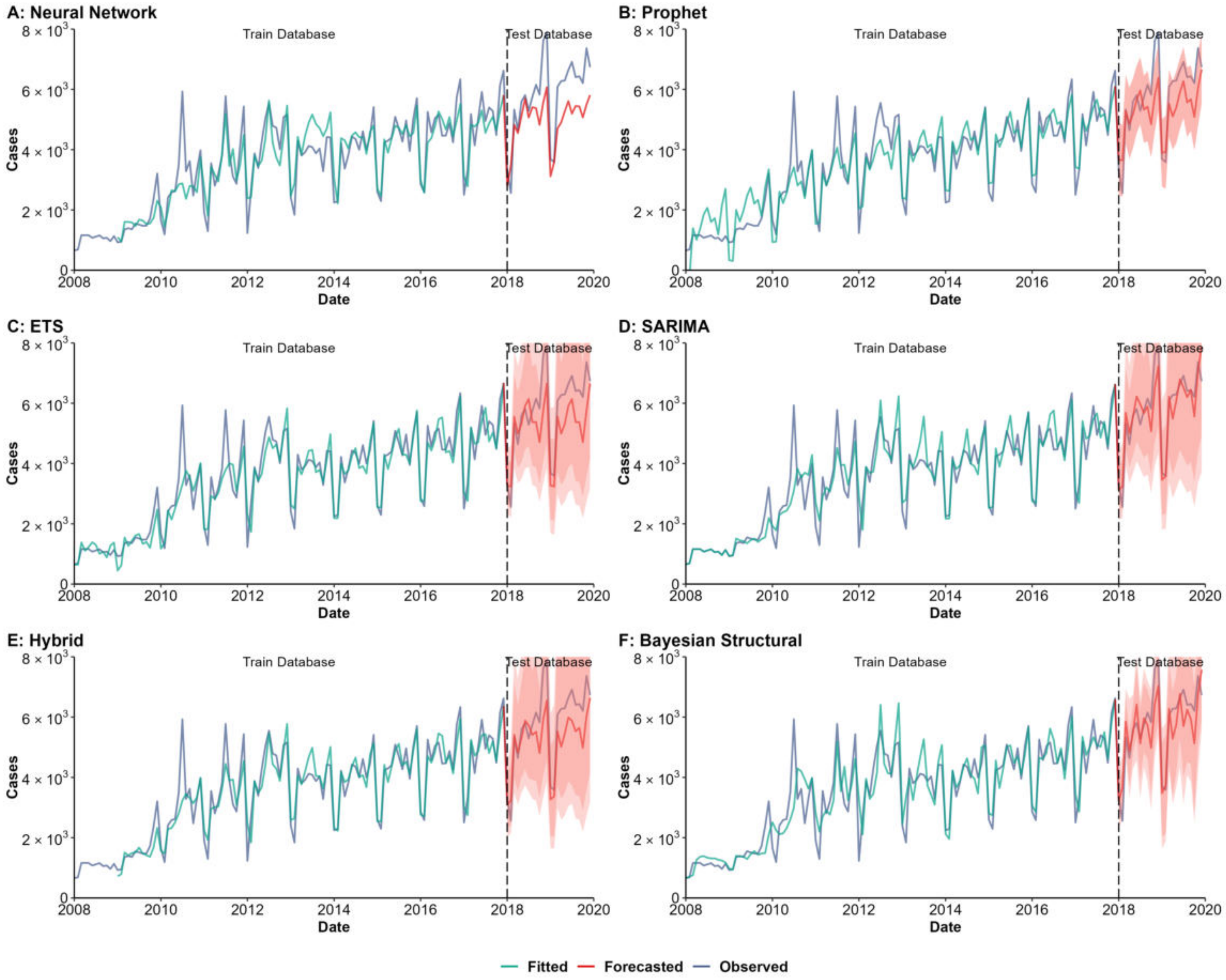

G : SMAPE of Models

Method	Train	Test	All
Neural Network	12.69	16.54	13.39
ETS	11.58	13.62	11.92
SARIMA	10.56	7.26	10.01
Hybrid*	10.25	12.17	10.60
Bayesian Structural	13.89	9.71	13.19
Prophet	19.54	12.87	18.43

*Hybrid: Combined SARIMA, ETS, STL and Neural Network model

H : RMSE of Models

Method	Train	Test	All
Neural Network	596.10	1073.93	707.41
ETS	495.77	906.91	584.73
SARIMA	551.30	518.54	545.98
Hybrid*	495.91	817.70	568.14
Bayesian Structural	612.22	659.41	620.33
Prophet	584.61	845.66	635.61

*Hybrid: Combined SARIMA, ETS, STL and Neural Network model

I : MASE of Models

Method	Train	Test	All
Neural Network	0.69	1.55	0.81
ETS	0.42	0.96	0.59
SARIMA	0.54	0.45	0.51
Hybrid*	0.39	0.92	0.58
Bayesian Structural	0.53	0.51	0.58
Prophet	0.55	1.11	0.70

*Hybrid: Combined SARIMA, ETS, STL and Neural Network model

J : R_Squared of Models

Method	Train	Test	All
Neural Network	0.81	0.81	0.82
ETS	0.90	0.73	0.90
SARIMA	0.87	0.85	0.90
Hybrid*	0.87	0.83	0.88
Bayesian Structural	0.84	0.78	0.87
Prophet	0.86	0.80	0.87

*Hybrid: Combined SARIMA, ETS, STL and Neural Network model

Supplementary Fig. 36. Training and comparing variant time series models for acquired immunodeficiency syndrome (AIDS).

(A) Neural Network model; **(B)** Prophet model; **(C)** Exponential smoothing (ETS) model; **(D)** Seasonal autoregressive integrated moving average (SARIMA) model; **(E)** Hybrid models combining SARIMA, ETS, STL (seasonal and trend decomposition using loess), and neural network model; **(F)** Bayesian structural model; **(G)** Root mean square error (RMSE) of variant models; **(H)** Symmetric mean absolute percentage error (SMAPE) of variant models; **(I)** Mean absolute scaled error (MASE) of variant models; **(J)** R-squared of variant models.

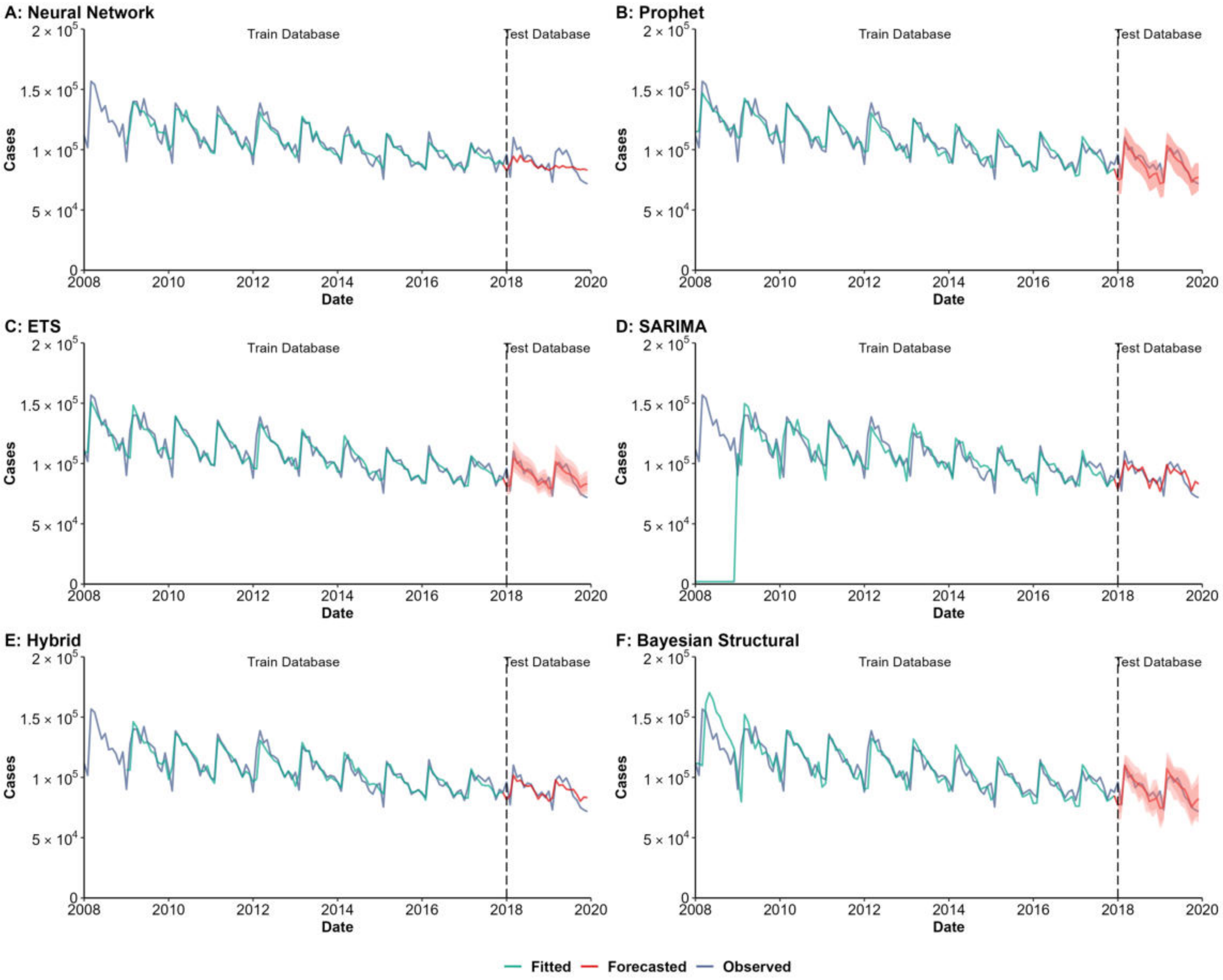

G : SMAPE of Models

Method	Train	Test	All
Neural Network	3.61	8.56	4.51
ETS	3.73	5.55	4.03
SARIMA	24.50	7.31	21.64
Hybrid*	3.85	6.24	4.28
Bayesian Structural	6.34	5.22	6.16
Prophet	4.17	5.62	4.41

*Hybrid: Combined SARIMA, ETS, STL and Neural Network model

H : RMSE of Models

Method	Train	Test	All
Neural Network	5048.30	9072.43	5984.72
ETS	5942.67	5897.21	5935.12
SARIMA	40903.93	7918.45	37479.69
Hybrid*	5951.85	6656.23	6085.99
Bayesian Structural	10818.75	6471.70	10223.42
Prophet	6199.41	6882.93	6318.47

*Hybrid: Combined SARIMA, ETS, STL and Neural Network model

I : MASE of Models

Method	Train	Test	All
Neural Network	0.66	3.25	0.87
ETS	0.43	1.10	0.67
SARIMA	2.03	0.99	1.90
Hybrid*	0.46	1.33	0.73
Bayesian Structural	0.76	0.72	0.79
Prophet	0.48	0.79	0.71

*Hybrid: Combined SARIMA, ETS, STL and Neural Network model

J : R_Squared of Models

Method	Train	Test	All
Neural Network	0.90	0.37	0.87
ETS	0.88	0.68	0.89
SARIMA	0.00	0.41	0.00
Hybrid*	0.85	0.63	0.86
Bayesian Structural	0.71	0.65	0.74
Prophet	0.87	0.68	0.87

*Hybrid: Combined SARIMA, ETS, STL and Neural Network model

Supplementary Fig. 37. Training and comparing variant time series models for tuberculosis.

(A) Neural Network model; **(B)** Prophet model; **(C)** Exponential smoothing (ETS) model; **(D)** Seasonal autoregressive integrated moving average (SARIMA) model; **(E)** Hybrid models combining SARIMA, ETS, STL (seasonal and trend decomposition using loess), and neural network model; **(F)** Bayesian structural model; **(G)** Root mean square error (RMSE) of variant models; **(H)** Symmetric mean absolute percentage error (SMAPE) of variant models; **(I)** Mean absolute scaled error (MASE) of variant models; **(J)** R-squared of variant models.

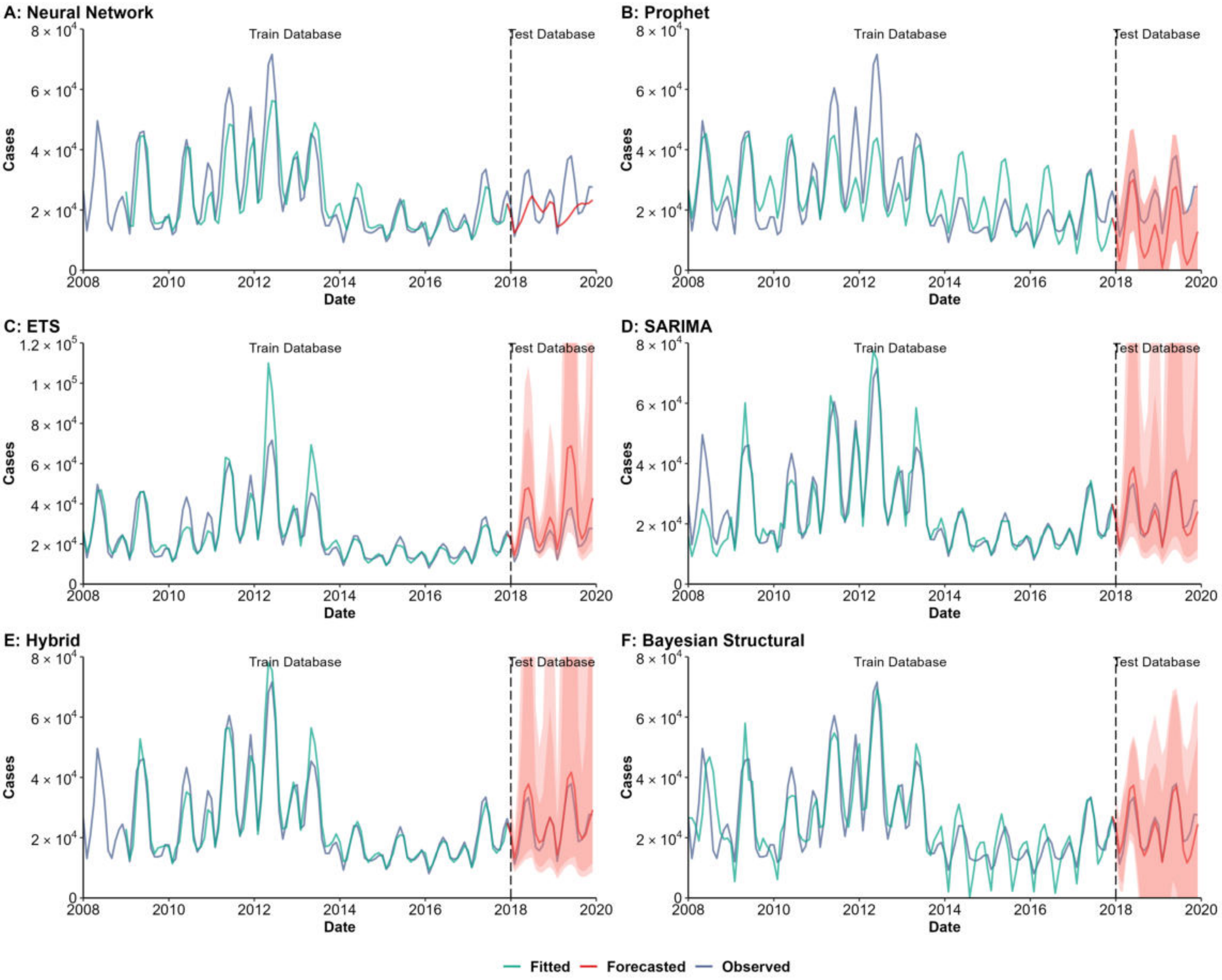

G : SMAPE of Models

Method	Train	Test	All
Neural Network	17.22	24.96	18.62
ETS	12.95	31.97	16.12
SARIMA	14.06	11.14	13.58
Hybrid*	10.19	10.15	10.18
Bayesian Structural	27.89	14.49	25.65
Prophet	27.47	73.75	35.18

*Hybrid: Combined SARIMA, ETS, STL and Neural Network model

H : RMSE of Models

Method	Train	Test	All
Neural Network	6262.36	7883.47	6586.85
ETS	6868.39	13611.02	8377.88
SARIMA	5460.31	3226.85	5155.70
Hybrid*	3679.01	3132.44	3585.83
Bayesian Structural	6631.07	3784.71	6247.39
Prophet	8652.71	11406.85	9169.36

*Hybrid: Combined SARIMA, ETS, STL and Neural Network model

I : MASE of Models

Method	Train	Test	All
Neural Network	0.85	2.96	1.01
ETS	0.58	1.09	0.67
SARIMA	0.51	0.46	0.50
Hybrid*	0.42	0.42	0.43
Bayesian Structural	0.77	0.49	0.65
Prophet	1.01	1.59	1.12

*Hybrid: Combined SARIMA, ETS, STL and Neural Network model

J : R_Squared of Models

Method	Train	Test	All
Neural Network	0.80	0.11	0.75
ETS	0.86	0.87	0.80
SARIMA	0.86	0.82	0.86
Hybrid*	0.93	0.91	0.93
Bayesian Structural	0.78	0.78	0.78
Prophet	0.59	0.76	0.51

*Hybrid: Combined SARIMA, ETS, STL and Neural Network model

Supplementary Fig. 38. Training and comparing variant time series models for mumps. **(A)** Neural Network model; **(B)** Prophet model; **(C)** Exponential smoothing (ETS) model; **(D)** Seasonal autoregressive integrated moving average (SARIMA) model; **(E)** Hybrid models combining SARIMA, ETS, STL (seasonal and trend decomposition using loess), and neural network model; **(F)** Bayesian structural model; **(G)** Root mean square error (RMSE) of variant models; **(H)** Symmetric mean absolute percentage error (SMAPE) of variant models; **(I)** Mean absolute scaled error (MASE) of variant models; **(J)** R-squared of variant models.

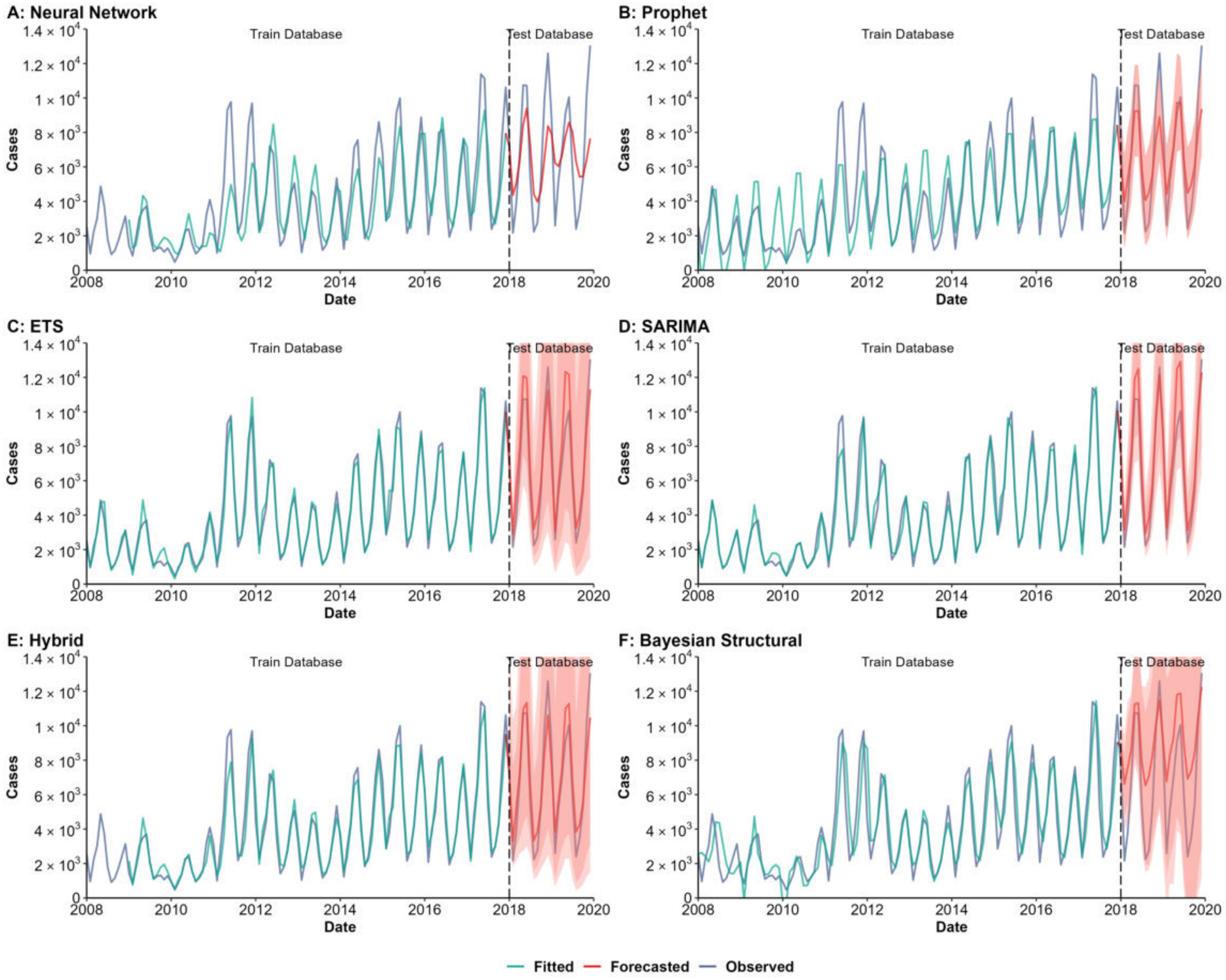

G : SMAPE of Models

Method	Train	Test	All
Neural Network	29.94	32.57	30.42
ETS	10.94	18.43	12.19
SARIMA	10.42	16.31	11.40
Hybrid*	13.07	18.71	14.09
Bayesian Structural	28.96	40.55	30.89
Prophet	37.36	25.23	35.34

*Hybrid: Combined SARIMA, ETS, STL and Neural Network model

H : RMSE of Models

Method	Train	Test	All
Neural Network	1559.38	2412.97	1745.90
ETS	507.57	1307.01	706.68
SARIMA	572.96	1233.43	726.04
Hybrid*	680.15	1231.46	808.84
Bayesian Structural	1171.60	2831.52	1574.84
Prophet	1374.52	1738.25	1441.53

*Hybrid: Combined SARIMA, ETS, STL and Neural Network model

I : MASE of Models

Method	Train	Test	All
Neural Network	0.84	1.62	0.97
ETS	0.21	0.41	0.26
SARIMA	0.24	0.32	0.26
Hybrid*	0.28	0.44	0.34
Bayesian Structural	0.52	1.52	0.78
Prophet	0.63	0.89	0.69

*Hybrid: Combined SARIMA, ETS, STL and Neural Network model

J : R_Squared of Models

Method	Train	Test	All
Neural Network	0.67	0.61	0.67
ETS	0.96	0.87	0.94
SARIMA	0.95	0.92	0.94
Hybrid*	0.94	0.89	0.93
Bayesian Structural	0.80	0.92	0.75
Prophet	0.73	0.88	0.77

*Hybrid: Combined SARIMA, ETS, STL and Neural Network model

Supplementary Fig. 39. Training and comparing variant time series models for scarlet fever.

(A) Neural Network model; **(B)** Prophet model; **(C)** Exponential smoothing (ETS) model; **(D)** Seasonal autoregressive integrated moving average (SARIMA) model; **(E)** Hybrid models combining SARIMA, ETS, STL (seasonal and trend decomposition using loess), and neural network model; **(F)** Bayesian structural model; **(G)** Root mean square error (RMSE) of variant models; **(H)** Symmetric mean absolute percentage error (SMAPE) of variant models; **(I)** Mean absolute scaled error (MASE) of variant models; **(J)** R-squared of variant models.

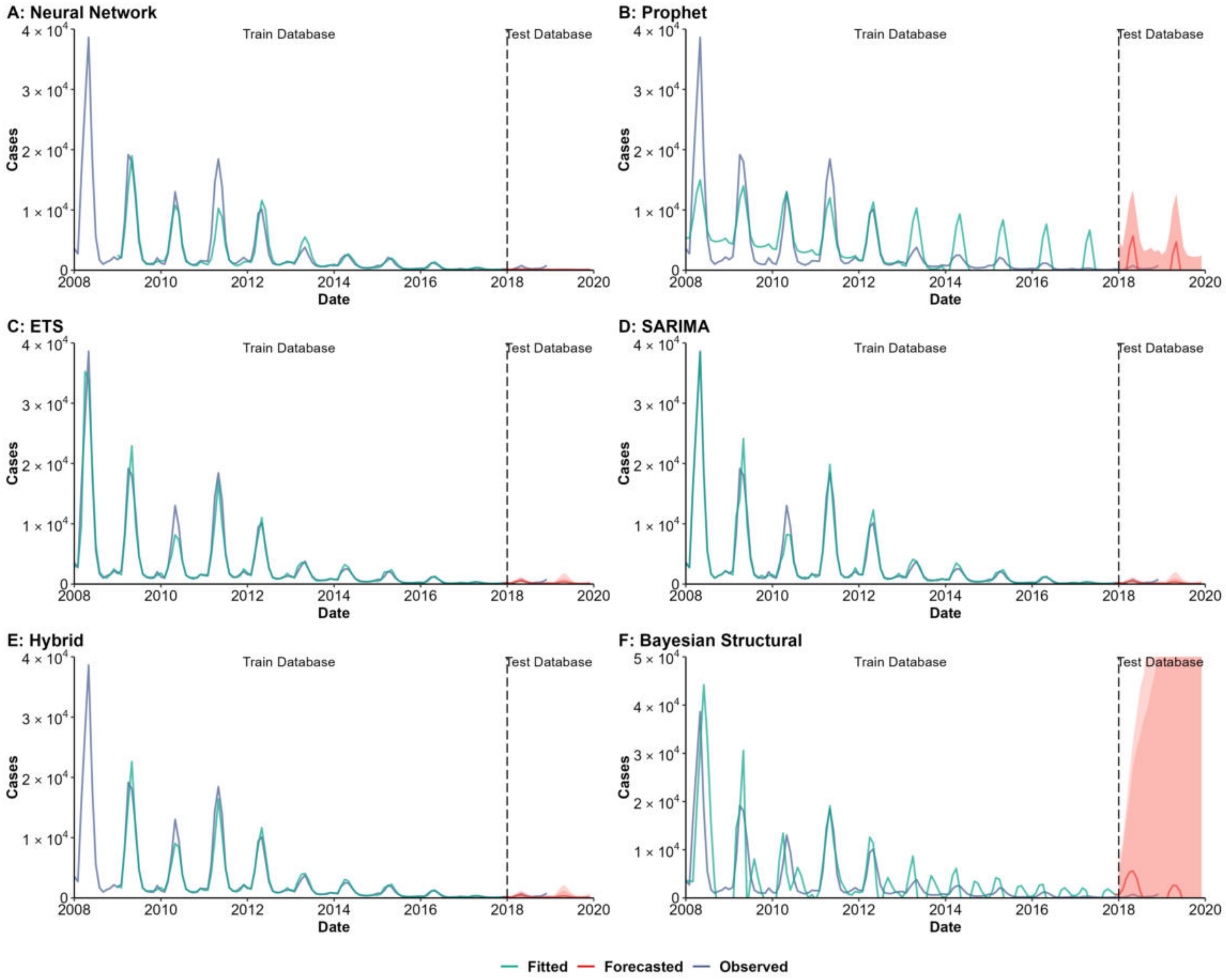

G : SMAPE of Models

Method	Train	Test	All
Neural Network	22.14	87.34	28.66
ETS	15.04	58.21	18.96
SARIMA	14.79	62.87	19.16
Hybrid*	13.87	62.14	18.70
Bayesian Structural	111.03	176.40	116.97
Prophet	98.40	184.97	106.27

*Hybrid: Combined SARIMA, ETS, STL and Neural Network model

H : RMSE of Models

Method	Train	Test	All
Neural Network	1574.38	316.31	1496.94
ETS	1409.08	234.49	1345.36
SARIMA	1058.88	243.11	1012.26
Hybrid*	962.14	246.44	916.09
Bayesian Structural	5302.93	3056.49	5139.45
Prophet	3850.75	3883.99	3853.78

*Hybrid: Combined SARIMA, ETS, STL and Neural Network model

I : MASE of Models

Method	Train	Test	All
Neural Network	0.59	12.59	0.61
ETS	0.33	2.10	0.35
SARIMA	0.23	2.55	0.24
Hybrid*	0.33	2.99	0.34
Bayesian Structural	1.80	2.26	0.96
Prophet	1.43	2.00	1.49

*Hybrid: Combined SARIMA, ETS, STL and Neural Network model

J : R_Squared of Models

Method	Train	Test	All
Neural Network	0.86	0.29	0.86
ETS	0.94	0.20	0.94
SARIMA	0.97	0.20	0.97
Hybrid*	0.95	0.23	0.95
Bayesian Structural	0.51	0.02	0.51
Prophet	0.56	0.26	0.53

*Hybrid: Combined SARIMA, ETS, STL and Neural Network model

Supplementary Fig. 40. Training and comparing variant time series models for rubella.

(A) Neural Network model; **(B)** Prophet model; **(C)** Exponential smoothing (ETS) model; **(D)** Seasonal autoregressive integrated moving average (SARIMA) model; **(E)** Hybrid models combining SARIMA, ETS, STL (seasonal and trend decomposition using loess), and neural network model; **(F)** Bayesian structural model; **(G)** Root mean square error (RMSE) of variant models; **(H)** Symmetric mean absolute percentage error (SMAPE) of variant models; **(I)** Mean absolute scaled error (MASE) of variant models; **(J)** R-squared of variant models.

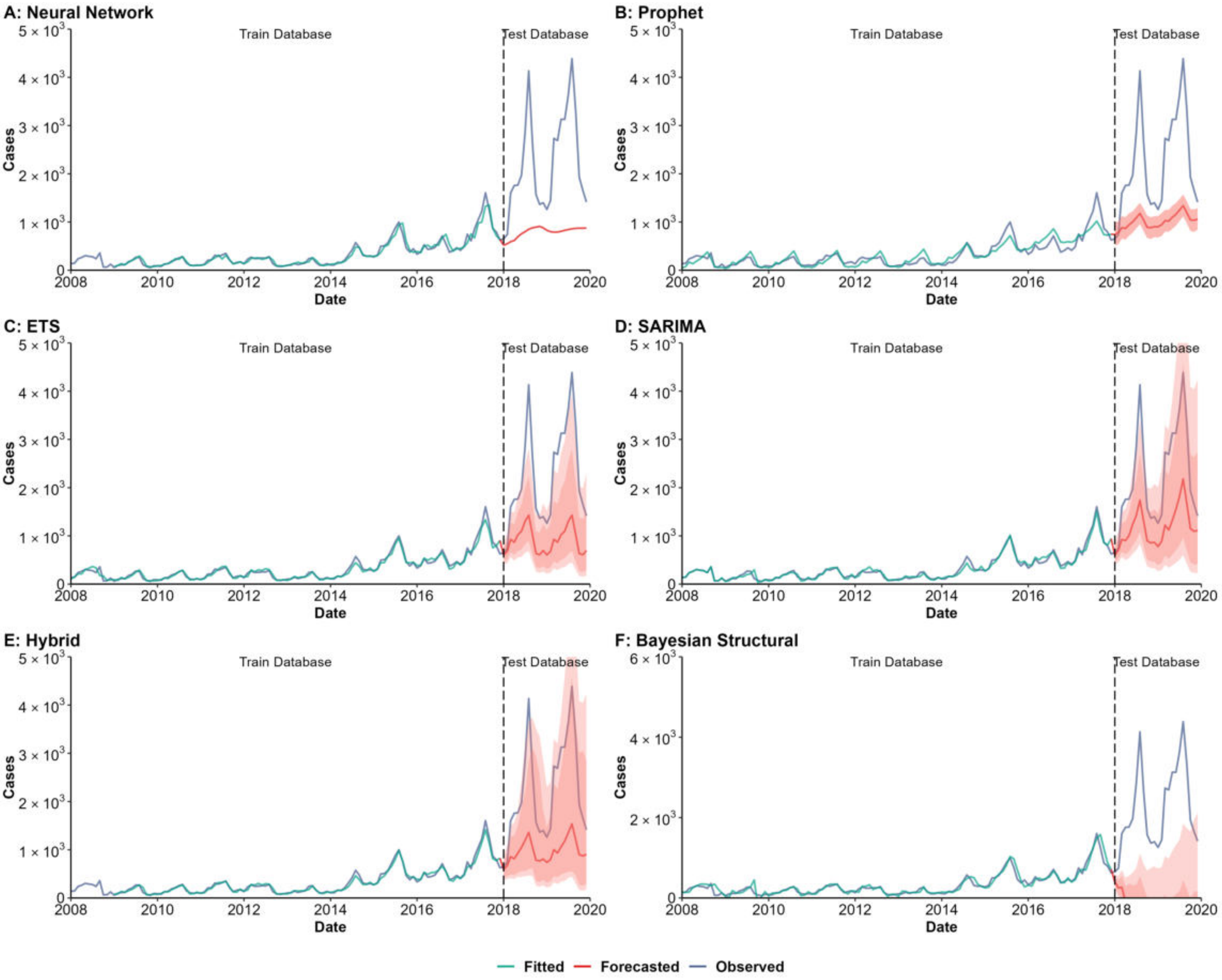

G : SMAPE of Models

Method	Train	Test	All
Neural Network	12.75	83.99	25.70
ETS	14.60	76.57	24.93
SARIMA	13.86	54.60	20.65
Hybrid*	11.44	69.55	22.00
Bayesian Structural	23.53	187.05	50.78
Prophet	29.54	65.67	35.56

*Hybrid: Combined SARIMA, ETS, STL and Neural Network model

H : RMSE of Models

Method	Train	Test	All
Neural Network	70.07	1720.43	736.33
ETS	62.62	1523.42	624.56
SARIMA	59.63	1221.93	501.81
Hybrid*	50.95	1470.03	628.52
Bayesian Structural	90.84	4006.62	1637.80
Prophet	118.75	1489.20	617.55

*Hybrid: Combined SARIMA, ETS, STL and Neural Network model

I : MASE of Models

Method	Train	Test	All
Neural Network	0.67	55.29	5.05
ETS	0.55	7.91	3.05
SARIMA	0.54	4.87	2.14
Hybrid*	0.44	8.94	3.26
Bayesian Structural	0.78	19.78	6.38
Prophet	1.14	17.72	4.23

*Hybrid: Combined SARIMA, ETS, STL and Neural Network model

J : R_Squared of Models

Method	Train	Test	All
Neural Network	0.94	0.18	0.55
ETS	0.95	0.77	0.76
SARIMA	0.95	0.91	0.89
Hybrid*	0.97	0.92	0.79
Bayesian Structural	0.90	0.09	0.39
Prophet	0.81	0.84	0.75

*Hybrid: Combined SARIMA, ETS, STL and Neural Network model

Supplementary Fig. 41. Training and comparing variant time series models for pertussis. **(A)** Neural Network model; **(B)** Prophet model; **(C)** Exponential smoothing (ETS) model; **(D)** Seasonal autoregressive integrated moving average (SARIMA) model; **(E)** Hybrid models combining SARIMA, ETS, STL (seasonal and trend decomposition using loess), and neural network model; **(F)** Bayesian structural model; **(G)** Root mean square error (RMSE) of variant models; **(H)** Symmetric mean absolute percentage error (SMAPE) of variant models; **(I)** Mean absolute scaled error (MASE) of variant models; **(J)** R-squared of variant models.

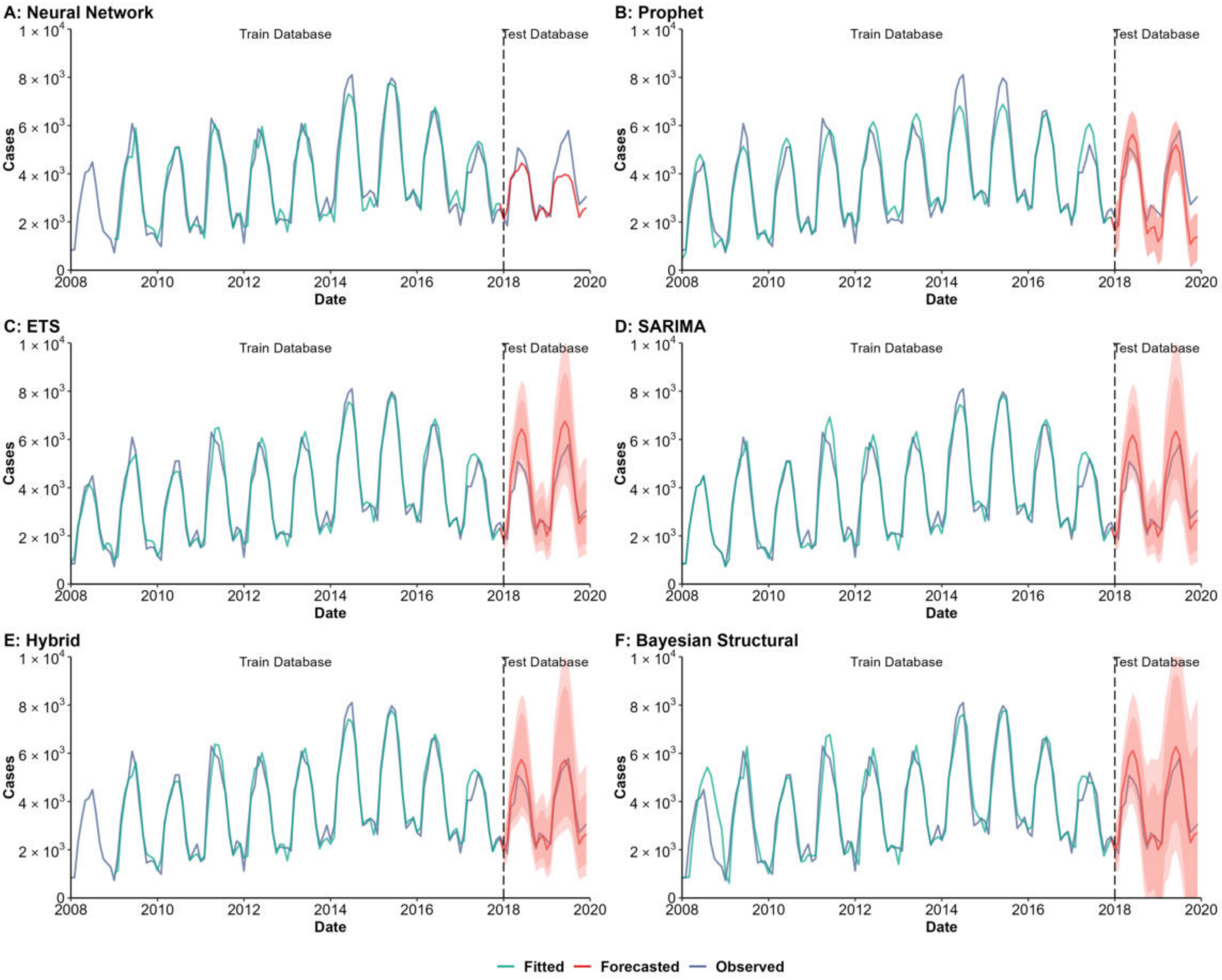

G : SMAPE of Models

Method	Train	Test	All
Neural Network	12.14	14.48	12.56
ETS	9.62	15.38	10.58
SARIMA	9.00	13.31	9.72
Hybrid*	8.87	10.70	9.20
Bayesian Structural	15.36	12.61	14.90
Prophet	11.07	28.19	13.92

*Hybrid: Combined SARIMA, ETS, STL and Neural Network model

H : RMSE of Models

Method	Train	Test	All
Neural Network	476.32	721.75	529.47
ETS	402.21	796.37	490.42
SARIMA	427.34	619.30	464.87
Hybrid*	383.89	431.13	392.90
Bayesian Structural	683.38	583.40	667.76
Prophet	509.86	879.70	587.89

*Hybrid: Combined SARIMA, ETS, STL and Neural Network model

I : MASE of Models

Method	Train	Test	All
Neural Network	0.47	1.29	0.56
ETS	0.37	0.78	0.46
SARIMA	0.37	0.66	0.42
Hybrid*	0.35	0.54	0.40
Bayesian Structural	0.58	0.63	0.60
Prophet	0.47	0.96	0.58

*Hybrid: Combined SARIMA, ETS, STL and Neural Network model

J : R_Squared of Models

Method	Train	Test	All
Neural Network	0.93	0.81	0.91
ETS	0.95	0.94	0.93
SARIMA	0.95	0.93	0.94
Hybrid*	0.95	0.92	0.95
Bayesian Structural	0.86	0.93	0.86
Prophet	0.92	0.82	0.89

*Hybrid: Combined SARIMA, ETS, STL and Neural Network model

Supplementary Fig. 42. Training and comparing variant time series models for brucellosis. **(A)** Neural Network model; **(B)** Prophet model; **(C)** Exponential smoothing (ETS) model; **(D)** Seasonal autoregressive integrated moving average (SARIMA) model; **(E)** Hybrid models combining SARIMA, ETS, STL (seasonal and trend decomposition using loess), and neural network model; **(F)** Bayesian structural model; **(G)** Root mean square error (RMSE) of variant models; **(H)** Symmetric mean absolute percentage error (SMAPE) of variant models; **(I)** Mean absolute scaled error (MASE) of variant models; **(J)** R-squared of variant models.

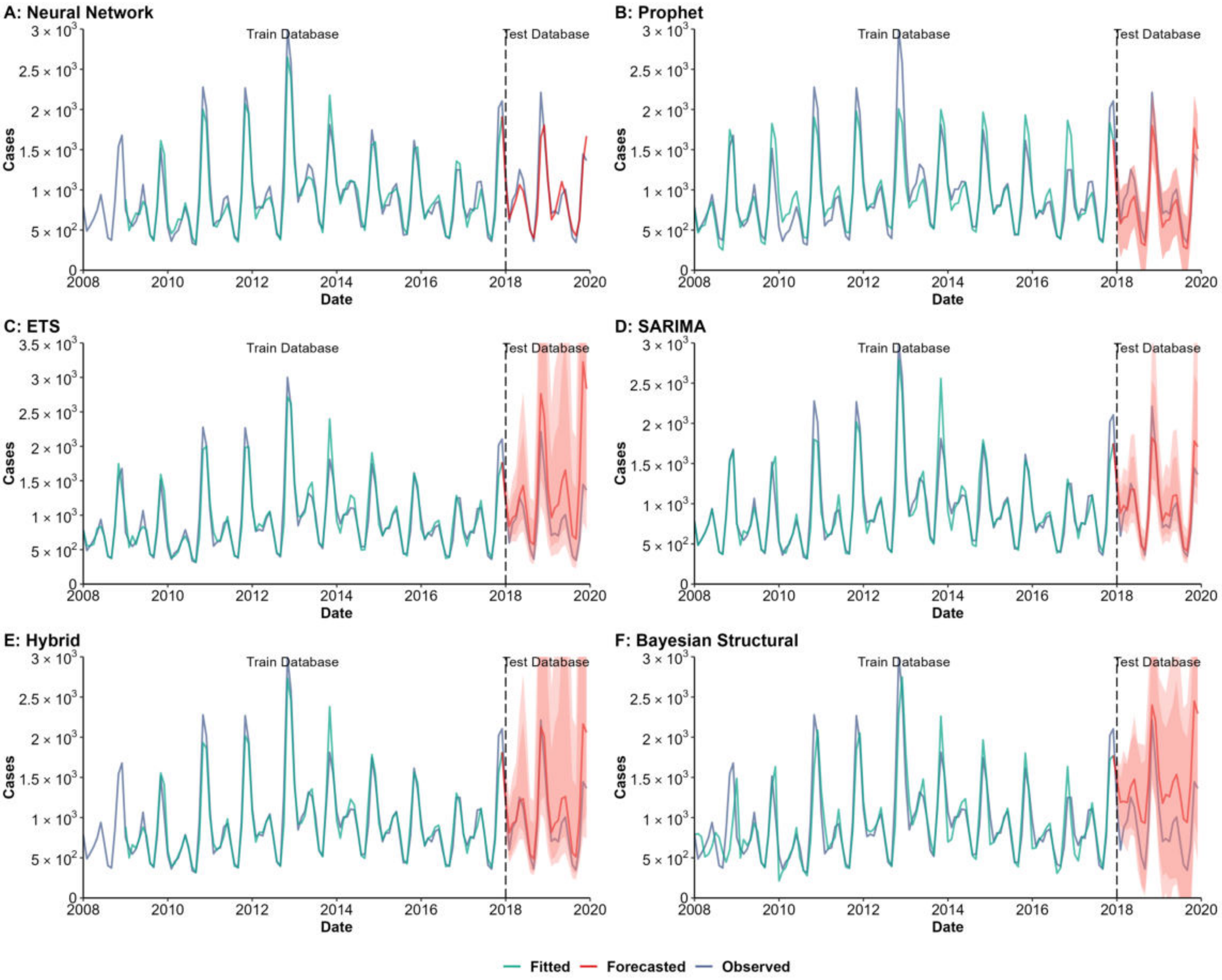

G : SMAPE of Models

Method	Train	Test	All
Neural Network	9.74	12.02	10.15
ETS	9.05	37.05	13.72
SARIMA	8.90	12.89	9.57
Hybrid*	7.92	19.72	10.07
Bayesian Structural	16.77	47.16	21.84
Prophet	14.22	21.09	15.37

*Hybrid: Combined SARIMA, ETS, STL and Neural Network model

H : RMSE of Models

Method	Train	Test	All
Neural Network	129.17	165.65	136.53
ETS	138.60	611.14	279.75
SARIMA	151.85	162.02	153.59
Hybrid*	127.49	266.81	162.00
Bayesian Structural	235.53	541.45	308.37
Prophet	207.85	204.60	207.31

*Hybrid: Combined SARIMA, ETS, STL and Neural Network model

I : MASE of Models

Method	Train	Test	All
Neural Network	0.32	0.41	0.33
ETS	0.29	0.98	0.45
SARIMA	0.29	0.44	0.31
Hybrid*	0.26	0.60	0.32
Bayesian Structural	0.50	1.77	0.65
Prophet	0.45	0.61	0.48

*Hybrid: Combined SARIMA, ETS, STL and Neural Network model

J : R_Squared of Models

Method	Train	Test	All
Neural Network	0.94	0.86	0.93
ETS	0.92	0.70	0.76
SARIMA	0.91	0.88	0.90
Hybrid*	0.94	0.84	0.89
Bayesian Structural	0.78	0.79	0.67
Prophet	0.82	0.87	0.82

*Hybrid: Combined SARIMA, ETS, STL and Neural Network model

Supplementary Fig. 43. Training and comparing variant time series models for hemorrhagic fever with renal syndrome (HFRS).

(A) Neural Network model; **(B)** Prophet model; **(C)** Exponential smoothing (ETS) model; **(D)** Seasonal autoregressive integrated moving average (SARIMA) model; **(E)** Hybrid models combining SARIMA, ETS, STL (seasonal and trend decomposition using loess), and neural network model; **(F)** Bayesian structural model; **(G)** Root mean square error (RMSE) of variant models; **(H)** Symmetric mean absolute percentage error (SMAPE) of variant models; **(I)** Mean absolute scaled error (MASE) of variant models; **(J)** R-squared of variant models.

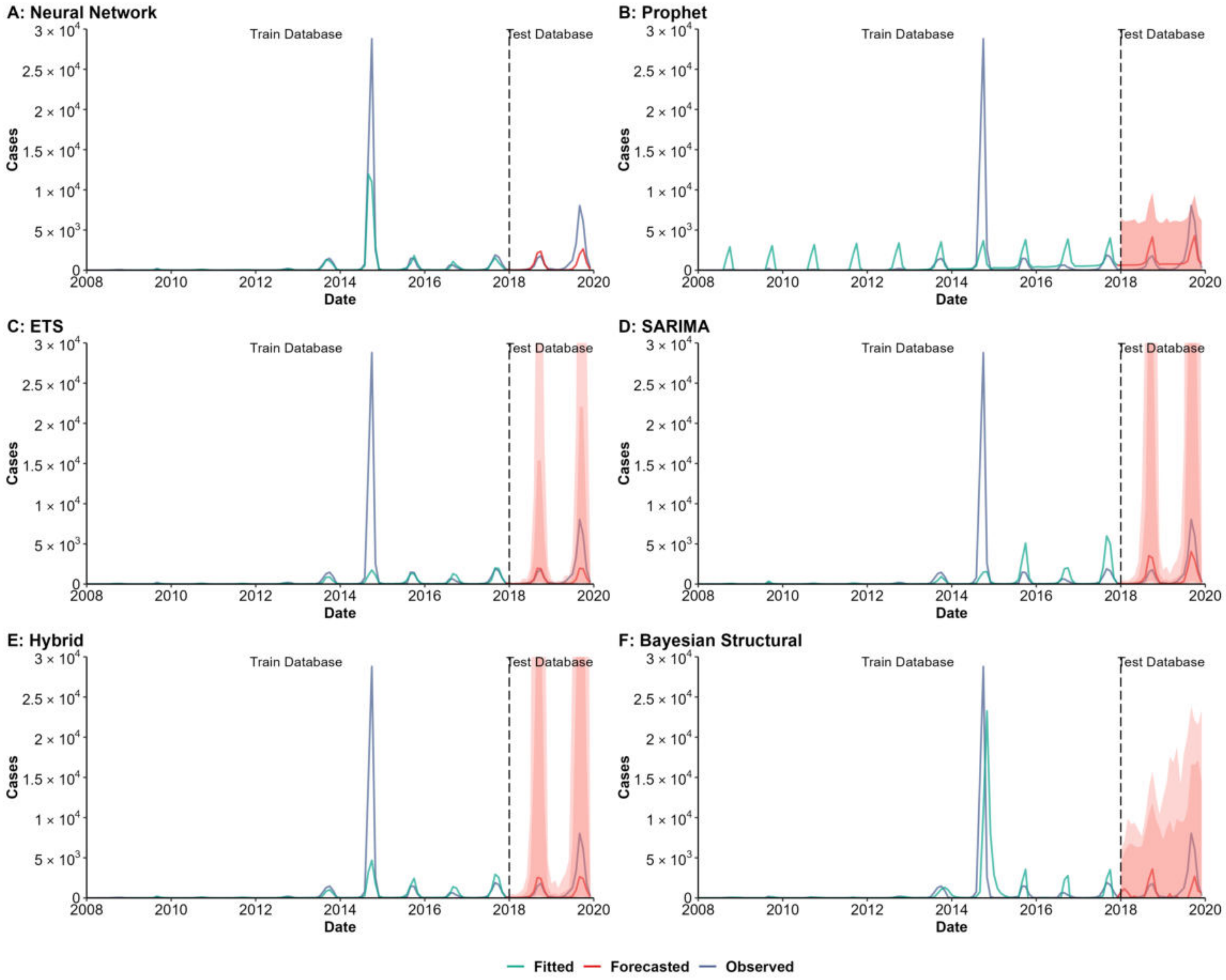

G : SMAPE of Models

Method	Train	Test	All
Neural Network	32.55	80.31	41.23
ETS	60.01	73.88	62.32
SARIMA	64.04	57.21	62.90
Hybrid*	48.62	69.78	52.47
Bayesian Structural	116.22	137.74	119.80
Prophet	150.99	109.97	144.15

*Hybrid: Combined SARIMA, ETS, STL and Neural Network model

H : RMSE of Models

Method	Train	Test	All
Neural Network	1740.62	1564.42	1709.94
ETS	2773.70	1648.69	2619.97
SARIMA	2873.25	1225.84	2670.22
Hybrid*	2582.23	1474.07	2418.81
Bayesian Structural	2967.59	1848.96	2812.22
Prophet	2733.37	1408.61	2560.63

*Hybrid: Combined SARIMA, ETS, STL and Neural Network model

I : MASE of Models

Method	Train	Test	All
Neural Network	0.74	1.70	0.96
ETS	0.71	2.17	2.92
SARIMA	1.98	1.02	1.69
Hybrid*	0.65	1.60	1.73
Bayesian Structural	1.29	1.34	1.17
Prophet	1.50	1.58	1.52

*Hybrid: Combined SARIMA, ETS, STL and Neural Network model

J : R_Squared of Models

Method	Train	Test	All
Neural Network	0.86	0.58	0.84
ETS	0.28	0.59	0.29
SARIMA	0.06	0.66	0.11
Hybrid*	0.60	0.62	0.57
Bayesian Structural	0.17	0.21	0.17
Prophet	0.14	0.53	0.17

*Hybrid: Combined SARIMA, ETS, STL and Neural Network model

Supplementary Fig. 44. Training and comparing variant time series models for dengue fever.

(A) Neural Network model; **(B)** Prophet model; **(C)** Exponential smoothing (ETS) model; **(D)** Seasonal autoregressive integrated moving average (SARIMA) model; **(E)** Hybrid models combining SARIMA, ETS, STL (seasonal and trend decomposition using loess), and neural network model; **(F)** Bayesian structural model; **(G)** Root mean square error (RMSE) of variant models; **(H)** Symmetric mean absolute percentage error (SMAPE) of variant models; **(I)** Mean absolute scaled error (MASE) of variant models; **(J)** R-squared of variant models.

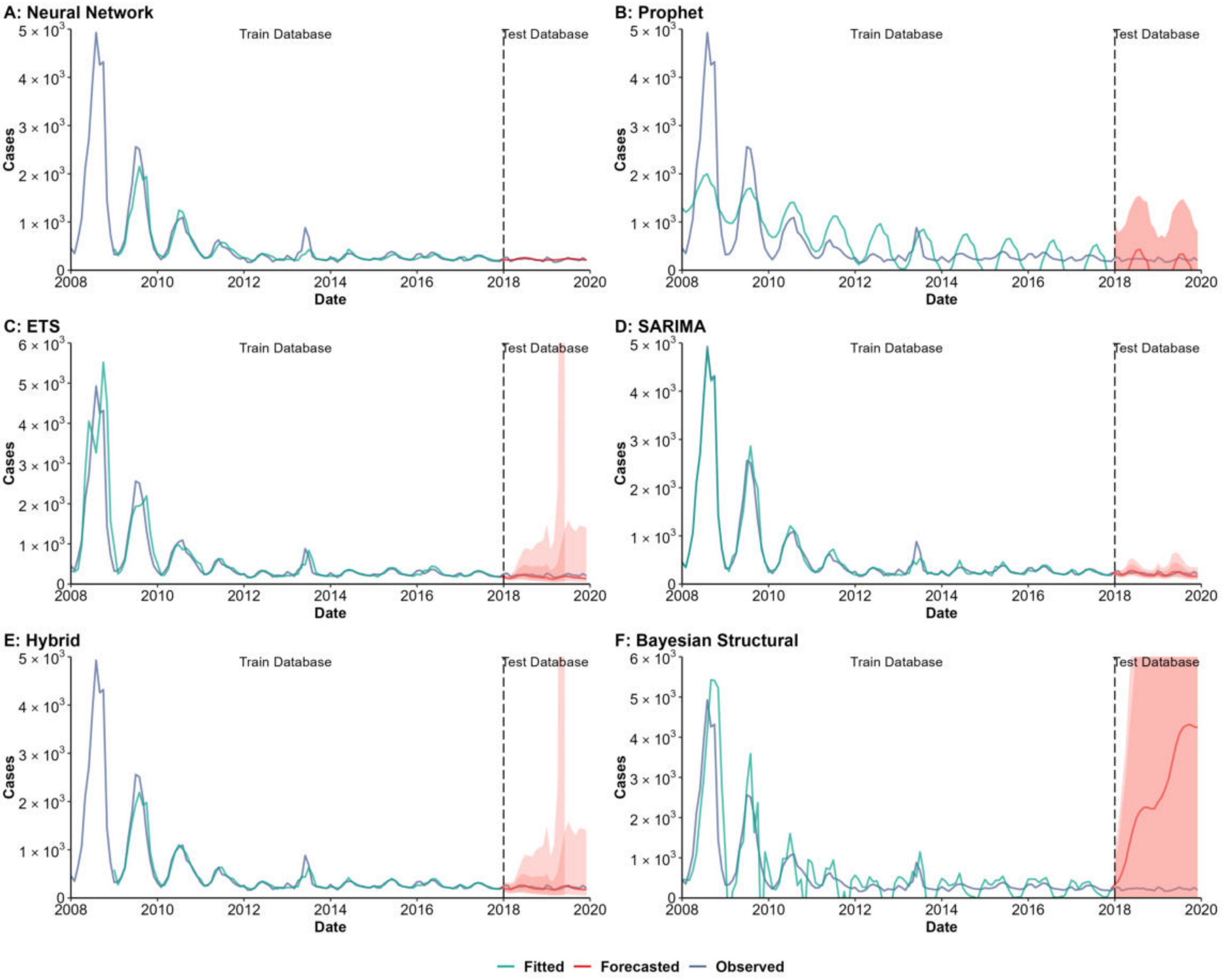

G : SMAPE of Models

Method	Train	Test	All
Neural Network	15.18	10.57	14.34
ETS	17.98	27.30	19.53
SARIMA	12.43	15.32	12.91
Hybrid*	11.52	13.11	11.81
Bayesian Structural	68.73	153.70	82.89
Prophet	81.68	126.76	89.20

*Hybrid: Combined SARIMA, ETS, STL and Neural Network model

H : RMSE of Models

Method	Train	Test	All
Neural Network	143.37	28.45	130.25
ETS	404.63	61.48	370.23
SARIMA	108.42	39.92	100.31
Hybrid*	118.88	36.39	108.65
Bayesian Structural	595.78	2665.33	1216.47
Prophet	594.28	401.53	566.73

*Hybrid: Combined SARIMA, ETS, STL and Neural Network model

I : MASE of Models

Method	Train	Test	All
Neural Network	0.91	3.19	0.96
ETS	0.91	3.48	0.88
SARIMA	0.33	1.53	0.36
Hybrid*	0.56	1.62	0.67
Bayesian Structural	2.00	13.03	1.88
Prophet	2.32	2.27	2.63

*Hybrid: Combined SARIMA, ETS, STL and Neural Network model

J : R_Squared of Models

Method	Train	Test	All
Neural Network	0.89	0.09	0.90
ETS	0.82	0.14	0.82
SARIMA	0.98	0.11	0.98
Hybrid*	0.92	0.13	0.92
Bayesian Structural	0.69	0.01	0.26
Prophet	0.52	0.05	0.49

*Hybrid: Combined SARIMA, ETS, STL and Neural Network model

Supplementary Fig. 45. Training and comparing variant time series models for malaria. **(A)** Neural Network model; **(B)** Prophet model; **(C)** Exponential smoothing (ETS) model; **(D)** Seasonal autoregressive integrated moving average (SARIMA) model; **(E)** Hybrid models combining SARIMA, ETS, STL (seasonal and trend decomposition using loess), and neural network model; **(F)** Bayesian structural model; **(G)** Root mean square error (RMSE) of variant models; **(H)** Symmetric mean absolute percentage error (SMAPE) of variant models; **(I)** Mean absolute scaled error (MASE) of variant models; **(J)** R-squared of variant models.

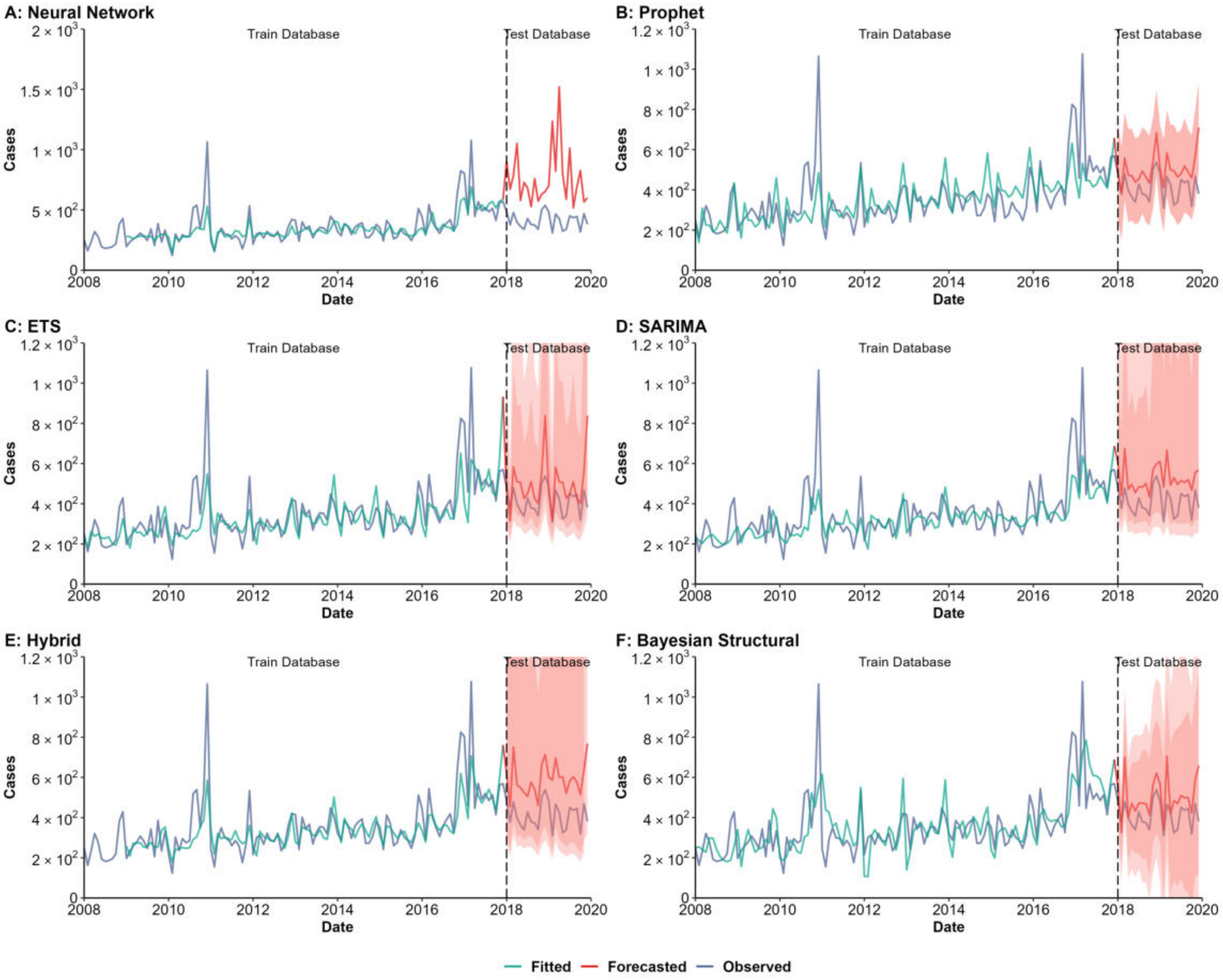

G : SMAPE of Models

Method	Train	Test	All
Neural Network	14.39	56.86	22.11
ETS	18.03	20.39	18.42
SARIMA	19.26	25.02	20.22
Hybrid*	15.36	36.33	19.18
Bayesian Structural	23.15	20.69	22.74
Prophet	19.67	20.28	19.77

*Hybrid: Combined SARIMA, ETS, STL and Neural Network model

H : RMSE of Models

Method	Train	Test	All
Neural Network	95.96	433.61	204.25
ETS	109.02	139.10	114.58
SARIMA	110.36	125.18	112.96
Hybrid*	96.63	193.75	120.27
Bayesian Structural	119.46	117.68	119.17
Prophet	112.35	113.36	112.52

*Hybrid: Combined SARIMA, ETS, STL and Neural Network model

I : MASE of Models

Method	Train	Test	All
Neural Network	1.31	1.35	1.32
ETS	0.68	0.82	0.95
SARIMA	1.51	1.54	1.52
Hybrid*	0.58	2.09	1.34
Bayesian Structural	0.83	0.82	1.06
Prophet	0.73	1.25	1.00

*Hybrid: Combined SARIMA, ETS, STL and Neural Network model

J : R_Squared of Models

Method	Train	Test	All
Neural Network	0.66	0.00	0.17
ETS	0.50	0.25	0.42
SARIMA	0.52	0.51	0.40
Hybrid*	0.64	0.34	0.40
Bayesian Structural	0.42	0.45	0.41
Prophet	0.44	0.30	0.40

*Hybrid: Combined SARIMA, ETS, STL and Neural Network model

Supplementary Fig. 46. Training and comparing variant time series models for echinococcosis.

(A) Neural Network model; **(B)** Prophet model; **(C)** Exponential smoothing (ETS) model; **(D)** Seasonal autoregressive integrated moving average (SARIMA) model; **(E)** Hybrid models combining SARIMA, ETS, STL (seasonal and trend decomposition using loess), and neural network model; **(F)** Bayesian structural model; **(G)** Root mean square error (RMSE) of variant models; **(H)** Symmetric mean absolute percentage error (SMAPE) of variant models; **(I)** Mean absolute scaled error (MASE) of variant models; **(J)** R-squared of variant models.

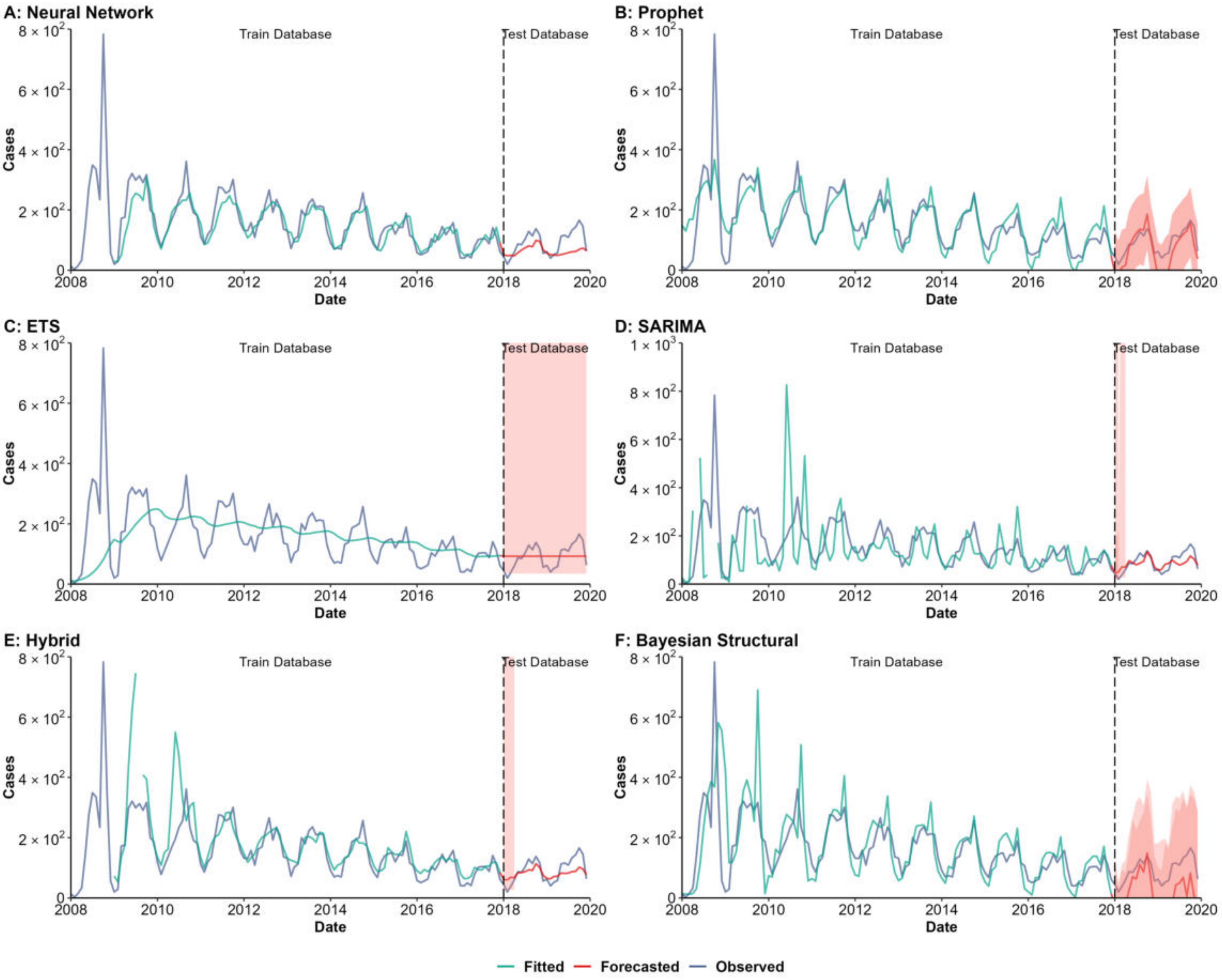

G : SMAPE of Models

Method	Train	Test	All
Neural Network	19.06	40.05	22.88
ETS	43.26	42.22	43.09
SARIMA	42.25	28.28	39.85
Hybrid*	23.23	30.53	24.57
Bayesian Structural	39.33	131.59	54.71
Prophet	32.34	74.13	39.31

*Hybrid: Combined SARIMA, ETS, STL and Neural Network model

H : RMSE of Models

Method	Train	Test	All
Neural Network	38.50	43.52	39.46
ETS	103.91	40.12	96.26
SARIMA	116.52	27.73	106.68
Hybrid*	78.30	30.21	71.94
Bayesian Structural	99.99	85.32	97.70
Prophet	61.06	40.85	58.18

*Hybrid: Combined SARIMA, ETS, STL and Neural Network model

I : MASE of Models

Method	Train	Test	All
Neural Network	1.09	5.40	1.30
ETS	1.42	37811.26	16.10
SARIMA	0.92	1.59	0.94
Hybrid*	1.19	3.31	1.19
Bayesian Structural	1.26	1.83	1.03
Prophet	0.82	0.99	0.95

*Hybrid: Combined SARIMA, ETS, STL and Neural Network model

J : R_Squared of Models

Method	Train	Test	All
Neural Network	0.75	0.38	0.74
ETS	0.05	0.29	0.09
SARIMA	0.11	0.64	0.14
Hybrid*	0.57	0.80	0.59
Bayesian Structural	0.40	0.54	0.44
Prophet	0.63	0.80	0.65

*Hybrid: Combined SARIMA, ETS, STL and Neural Network model

Supplementary Fig. 47. Training and comparing variant time series models for typhus.

(A) Neural Network model; **(B)** Prophet model; **(C)** Exponential smoothing (ETS) model; **(D)** Seasonal autoregressive integrated moving average (SARIMA) model; **(E)** Hybrid models combining SARIMA, ETS, STL (seasonal and trend decomposition using loess), and neural network model; **(F)** Bayesian structural model; **(G)** Root mean square error (RMSE) of variant models; **(H)** Symmetric mean absolute percentage error (SMAPE) of variant models; **(I)** Mean absolute scaled error (MASE) of variant models; **(J)** R-squared of variant models.

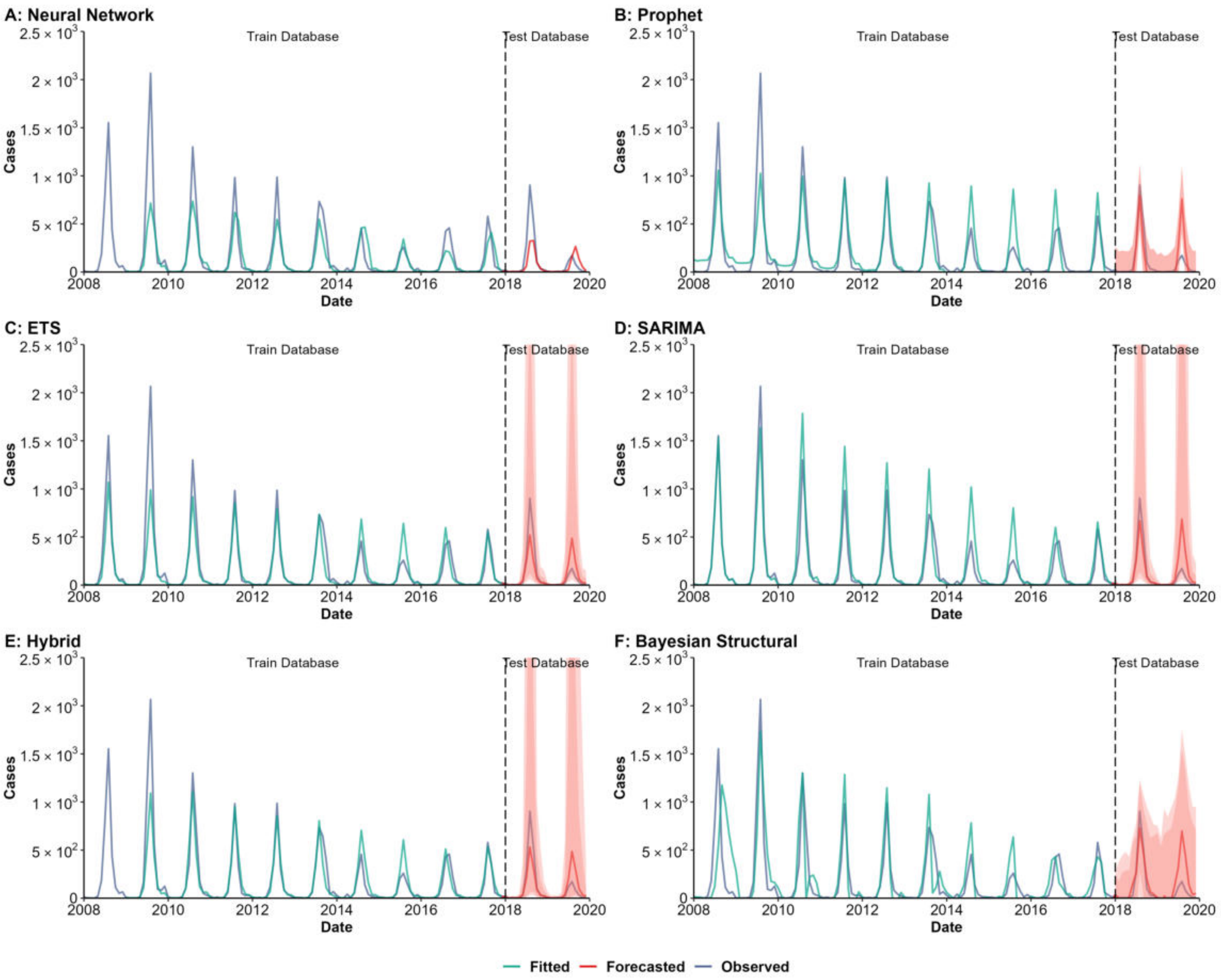

G : SMAPE of Models

Method	Train	Test	All
Neural Network	56.47	71.44	59.20
ETS	45.10	66.28	48.63
SARIMA	50.29	66.36	52.97
Hybrid*	46.67	61.37	49.34
Bayesian Structural	120.67	116.21	119.93
Prophet	114.71	165.91	123.24

*Hybrid: Combined SARIMA, ETS, STL and Neural Network model

H : RMSE of Models

Method	Train	Test	All
Neural Network	186.43	142.46	179.24
ETS	155.98	123.15	151.00
SARIMA	147.68	137.83	146.09
Hybrid*	138.15	120.42	135.10
Bayesian Structural	239.91	150.99	227.51
Prophet	173.93	180.99	175.13

*Hybrid: Combined SARIMA, ETS, STL and Neural Network model

I : MASE of Models

Method	Train	Test	All
Neural Network	0.89	1.25	0.94
ETS	0.40	0.70	0.51
SARIMA	0.36	0.57	0.38
Hybrid*	0.39	0.69	0.46
Bayesian Structural	0.88	0.64	0.70
Prophet	0.66	0.93	0.71

*Hybrid: Combined SARIMA, ETS, STL and Neural Network model

J : R_Squared of Models

Method	Train	Test	All
Neural Network	0.74	0.64	0.73
ETS	0.82	0.64	0.81
SARIMA	0.85	0.60	0.83
Hybrid*	0.82	0.66	0.81
Bayesian Structural	0.56	0.61	0.57
Prophet	0.72	0.63	0.69

*Hybrid: Combined SARIMA, ETS, STL and Neural Network model

Supplementary Fig. 48. Training and comparing variant time series models for Japanese encephalitis (JE).

(A) Neural Network model; **(B)** Prophet model; **(C)** Exponential smoothing (ETS) model; **(D)** Seasonal autoregressive integrated moving average (SARIMA) model; **(E)** Hybrid models combining SARIMA, ETS, STL (seasonal and trend decomposition using loess), and neural network model; **(F)** Bayesian structural model; **(G)** Root mean square error (RMSE) of variant models; **(H)** Symmetric mean absolute percentage error (SMAPE) of variant models; **(I)** Mean absolute scaled error (MASE) of variant models; **(J)** R-squared of variant models.

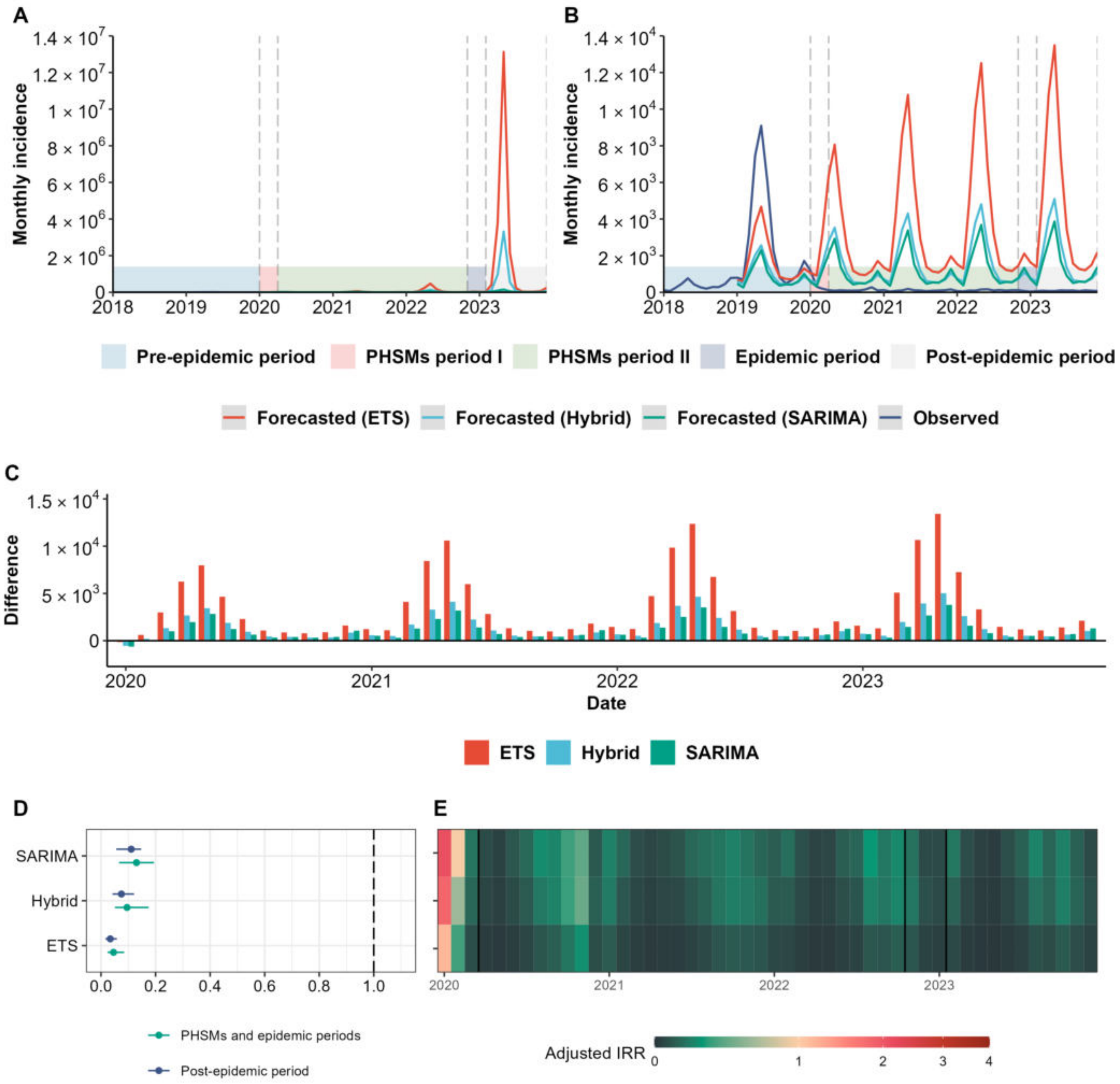

Supplementary Fig. 49. Training and comparing variant time series models for rubella.

(A) The forecasted number of rubella cases in the China from 2020 to 2023 trained on 2008-2019 data. **(B)** The forecasted number of rubella cases in the China from 2019 to 2023 trained on 2008-2018 data. **(C)** The difference between the forecasted incidence and the observed incidence of rubella in the China from 2020 to 2023, based on the model trained on 2008-2018 data. **(D)** The adjusted incidence relative ratio (IRR) distribution of rubella during different period which split by October 2022. **(E)** The changes of adjusted IRR of rubella during different period.

Supplementary Table 1. Reported infections of 24 Notifiable Infectious Diseases by Province in China

year	disease_en	Anhui	Beijing	Chongqing	Fujian	Gansu	Guangdong	Guangxi	Guizhou	Hainan	Hebei	Heilongjiang	Henan	Hubei	Hunan	Inner Mongolia	Jiangsu	Jiangxi	Jilin	Liaoning	Nation (Onset)	Ningxia	Qinghai	Shaanxi	Shandong	Shanghai	Shanxi	Sichuan	Tianjin	Tibet	Xinjiang	Yunnan	Zhejiang	Nation (Report)
2008	AHC	458	487	1123	160	178	2171	1763	2799	109	1204	12	290	1011	857	181	121	318	34	87	22743	62	40	162	150	187	213	1301	125	29	124	139	6848	
2008	AIDS	191	54	182	117	22	958	2382	172	22	88	65	1784	266	407	11	134	114	46	52	10059	6	9	50	41	132	99	543	29	5	218	1701	159	12409
2008	Brucellosis	2	12	0	4	6	18	0	0	0	3173	3670	593	0	1	11105	5	0	2139	484	27767	94	0	1034	135	2	4834	1	14	2	395	10	34	30002
2008	Dengue fever	1	2	1	24	1	87	1	0	2	0	0	1	3	7	0	4	1	0	2	202	0	0	2	0	5	0	0	0	0	0	51	7	206
2008	Dysentery	9324	24823	9766	2027	13566	8386	9200	12824	1154	21174	7532	18405	14400	10728	3239	10272	10388	5214	9490	312522	5186	1237	13183	12579	2264	6213	20091	12261	1153	16429	7113	12901	317367
2008	Echinococcosis	0	0	1	1	174	2	0	0	0	1	4	3	0	1	100	5	0	1	0	3033	358	236	14	0	1	2	1276	0	1	847	3	2	
2008	Enteric fever	215	16	180	434	54	1563	1164	1784	29	262	31	159	477	1058	26	377	435	20	257	15641	16	3	29	136	52	273	326	20	24	471	4389	1361	16409
2008	Gonorrhea	3827	2162	3549	5437	934	20001	9365	1598	655	951	1964	2755	3480	2906	1538	13487	3307	2036	2423	130818	1051	205	1780	2973	7696	1335	6202	609	34	2720	2230	21608	134783
2008	HFMD	26454	18437	4686	11715	5867	48876	27598	6505	7992	29560	13905	11826	24634	27113	7730	29066	5899	9889	14484	488955	2215	634	11175	32974	20095	19612	10021	7839	20	4014	11400	36720	
2008	HFRS	105	17	20	128	25	208	6	75	0	212	1892	128	163	487	81	244	347	880	737	9039	1	0	1412	1154	3	26	99	19	0	1	19	550	9696
2008	Hepatitis A	1561	267	1902	1140	4402	1734	1739	5941	394	1224	465	4243	2084	1472	649	1953	1853	443	1863	56052	1136	838	1290	622	309	913	5089	48	511	3360	5150	1457	58820
2008	Hepatitis B	33224	4564	28093	49100	65700	120679	37337	32931	5946	55485	20672	164408	73695	38098	32645	15924	34249	16761	29903	1169569	8522	17986	30131	35027	6264	42132	63345	2829	466	49504	19648	34301	1330654
2008	Hepatitis C	1587	1533	1598	1262	5474	10507	6427	1825	560	2748	4288	18932	3866	3334	3750	1785	1076	5781	5031	108446	284	1312	3426	1353	334	4152	3185	408	7	7571	3256	1794	118201
2008	Hepatitis E	1199	422	155	597	86	2016	517	116	37	808	370	552	1381	346	107	2874	386	259	1339	18525	29	25	141	843	459	127	422	225	0	214	579	1894	19679
2008	Infectious diarrhea	30478	45471	16301	14049	6331	92202	20833	5095	3642	42853	3644	22709	16268	16814	1607	9023	21173	1014	14677	652306	8120	1038	16128	26351	4820	8929	36353	41627	51	18907	5328	100470	
2008	JE	223	0	291	24	68	86	134	320	23	21	0	315	51	150	1	46	48	0	0	2975	0	0	41	114	13	26	573	2	2	1	338	64	3237
2008	Malaria	13484	36	43	23	13	121	57	1285	1844	29	6	3041	1083	73	2	558	24	13	17	26358	3	0	47	165	111	5	161	4	32	3	3640	435	26909
2008	Mumps	15789	3244	9224	4921	8944	17465	16689	13359	1980	11288	5257	16815	12426	8019	4297	14004	7411	7204	10957	310826	3139	1594	10205	10258	5404	4561	23879	4567	2175	16904	10599	28248	
2008	Pertussis	117	7	36	34	108	37	24	83	0	190	9	63	56	62	4	60	38	1	2	2387	8	11	87	106	2	74	578	37	1	388	54	110	2514
2008	Rubella	3619	1473	5338	1630	5360	1172	3192	1962	1320	6388	3294	2146	6727	2876	1923	2115	1889	10008	5630	120354	1072	326	1607	1275	715	2442	4244	5573	486	6019	9369	19164	
2008	Scarlet fever	374	1720	310	164	649	353	164	362	1	1153	3223	694	262	148	1292	1104	20	2090	3708	27782	373	146	601	1991	685	1002	953	861	25	1652	886	816	28507
2008	Syphilis	5823	3495	6955	14353	1996	28687	22947	2644	1566	1604	5479	6184	5693	6715	3526	19324	4610	4188	6957	235052	943	750	2358	3620	12931	4497	8854	2974	35	7532	2804	35008	272938
2008	Tuberculosis	54147	9576	35936	31146	33573	99596	62654	68841	11759	45523	39110	86903	61294	63468	21190	46489	39448	21962	25966	1169540	3631	4878	32702	42358	7163	25505	77834	3789	1890	42514	28085	40610	1533475
2008	Typhus	11	2	6	55	3	194	41	0	37	555	5	30	0	13	12	0	1	0	107	2398	0	0	16	125	0	94	103	9	1	0	978	0	
2009	AHC	205	422	680	193	88	1762	2011	452	142	1296	17	334	665	744	158	97	167	156	37	13707	133	57	181	113	145	183	1143	99	3	102	211	1711	12580
2009	AIDS	224	101	301	182	39	1237	3520	238	33	78	64	1851	292	540	12	206	157	71	75	13281	10	11	81	102	156	102	867	39	3	406	2038	245	19313
2009	Brucellosis	7	17	2	3	84	27	1	3	0	3218	4724	703	0	0	16551	6	0	3452	509	35816	157	0	911	156	0	4768	1	24	1	441	12	38	37734
2009	Dengue fever	1	4	7	12	0	19	25	0	1	0	0	0	3	2	0	2	0	0	1	305	0	1	1	0	1	0	0	0	0	0	21	204	312
2009	Dysentery	9527	23519	9379	1611	13629	6699	7922	11193	1132	18843	5489	14843	12302	10889	2542	7380	8295	3659	7326	262111	2859	1111	9889	9563	1280	5611	17510	9917	1865	12276	7320	6731	275123
2009	Echinococcosis	3	2	1	0	334	1	0	0	0	1	1	7	3	1	69	2	0	0	0	2676	485	306	17	4	2	2	455	0	6	965	8	1	2876
2009	Enteric fever	196	18	270	426	81	1745	1327	1643	21	274	34	138	552	1272	24	438	383	9	302	16938	7	7	32	55	51	241	340	11	0	823	5347	871	17409

2009	Gonorrhea	3519	1932	3013	5580	859	19598	8769	1523	723	926	1726	2252	3000	2883	1605	10921	3425	1931	2313	119824	828	219	1677	2669	6617	1228	5955	468	60	2051	1818	19736	121948
2009	HFMD	56697	24483	9164	19314	12908	93078	45644	12544	11913	110139	36237	101665	33211	34675	32253	83955	17955	25258	36584	1155525	5418	2436	29276	139261	24284	33336	22317	10125	403	6420	15071	69501	1149045
2009	HFRS	111	12	16	203	14	205	6	84	0	222	1701	161	250	483	141	277	341	910	720	8745	0	0	1404	922	3	29	63	19	0	2	15	431	9203
2009	Hepatitis A	1065	156	1520	1086	3192	1767	1406	3646	255	788	394	4036	1880	1254	482	1406	1333	449	1449	43841	1040	649	984	514	243	729	4777	37	403	1893	3929	1079	45372
2009	Hepatitis B	32256	3821	21038	52644	62296	130852	41035	35947	6264	57309	20240	158728	79745	40684	31976	14282	35784	17820	27839	1179607	6794	22909	32232	33569	5704	42747	63091	2421	449	45153	20785	33193	1330352
2009	Hepatitis C	2126	1496	1646	1785	6616	12590	8133	2436	642	3824	5295	22832	4798	4520	4596	1801	1383	7312	5573	131849	328	1855	3808	1478	339	4715	4689	397	14	8528	4357	1937	141609
2009	Hepatitis E	1393	393	168	663	76	2277	610	132	37	814	531	395	1608	455	76	3238	472	287	1241	20275	30	39	153	1195	508	131	528	167	7	217	522	1912	20854
2009	Infectious diarrhea	34027	46701	20053	15557	6290	89195	21414	5608	3111	49269	3366	27326	17468	20941	1750	9095	24862	1227	12067	655658	7100	1688	16694	25014	4916	9869	36141	39877	76	17810	7042	80104	597623
2009	JE	213	0	213	45	54	81	119	633	11	13	3	764	127	170	0	64	54	0	1	3913	0	0	162	131	13	53	350	0	1	2	527	109	4236
2009	Malaria	5918	28	47	65	43	83	51	864	685	39	9	1617	712	102	4	341	16	11	35	14098	0	1	63	111	58	7	185	3	43	6	2731	220	14453
2009	Mumps	12893	3002	14814	5252	5299	17755	19420	10270	4022	12199	4408	16593	17413	13514	2884	10134	9719	6357	9788	299329	5445	1078	10534	9444	4388	5707	23707	2409	267	8138	9847	22629	270307
2009	Pertussis	83	6	13	14	66	23	7	65	0	414	27	19	27	56	3	43	13	17	7	1612	5	6	26	119	0	31	277	19	0	123	48	55	1641
2009	Rubella	4349	551	1127	3122	5702	972	1012	2078	39	2174	1350	2622	3083	6036	1157	1597	1925	1482	1775	69860	3225	142	1600	1300	461	1265	2617	882	1669	2289	4054	8203	68683
2009	Scarlet fever	228	1193	144	163	706	262	116	334	0	1087	2643	531	196	143	1140	845	13	1186	3095	22068	507	123	682	1185	436	1341	860	497	63	1156	544	649	22441
2009	Syphilis	8302	4002	7206	16489	3003	35400	30351	4640	2060	2251	7036	9669	7908	10684	4442	24641	5408	4813	9273	306381	1094	1310	3619	5463	15103	5950	15053	3186	41	10563	4279	43142	320748
2009	Tuberculosis	44265	8029	27509	24706	29229	104702	49350	60464	9405	44069	38684	77771	51801	59293	19804	44623	41943	23891	25436	1076938	3454	5141	28530	43102	6739	24235	70998	3165	3395	39708	26937	36560	1482869
2009	Typhus	7	3	1	40	7	213	78	2	47	698	8	21	2	9	2	1	0	0	75	2764	0	0	5	117	1	46	81	11	0	0	1282	7	2574
2010	AHC	7030	288	11770	4978	244	72218	83023	1174	11503	1398	31	1863	23916	11456	207	2409	5953	154	55	290767	106	37	3048	1478	953	190	18767	93	63	66	1516	24780	292369
2010	AIDS	334	176	425	236	55	1157	4085	304	31	119	110	1498	355	745	30	325	233	98	110	15982	19	22	129	107	275	162	1586	58	4	539	2200	455	36594
2010	Brucellosis	4	29	0	5	46	35	3	1	1	2503	4861	791	5	9	16224	5	0	2905	606	33772	207	3	525	222	0	3888	2	56	0	797	8	31	35693
2010	Dengue fever	4	10	0	20	0	139	0	1	0	0	1	0	2	12	0	3	2	0	1	223	0	0	0	2	1	0	1	0	0	0	18	6	237
2010	Dysentery	12159	23231	8925	1627	11374	6453	7145	9824	1091	18194	5727	14726	12302	10722	2322	6827	8692	3726	7030	252248	2106	1060	9769	9727	1197	5146	16653	8879	970	9417	8368	6859	255331
2010	Echinococcosis	0	2	3	0	333	2	0	0	1	2	1	7	1	1	101	5	1	1	1	2515	309	335	11	4	0	3	306	0	25	1050	8	2	4738
2010	Enteric fever	159	21	95	420	52	1572	1194	997	22	281	19	127	291	975	31	230	360	14	184	14041	7	19	32	37	55	235	305	5	3	720	4860	719	14712
2010	Gonorrhea	3083	1550	2745	5186	942	17406	7341	1478	742	969	1459	1866	2700	2613	1472	8052	3149	1748	2205	105544	648	208	1809	2619	5421	1171	4910	421	80	2020	1819	17712	107373
2010	HFMD	85155	45409	19970	57612	12440	226620	164197	31250	25128	95901	7697	96741	66475	112197	18944	86634	30646	28838	41010	1774669	8805	4560	58564	141468	40906	24690	43769	28178	1949	6883	48589	113444	1795336
2010	HFRS	135	15	9	171	5	247	11	94	0	220	1486	186	226	440	91	294	396	765	753	9526	1	0	2408	980	1	33	65	16	0	0	15	463	9782
2010	Hepatitis A	975	127	1104	775	2661	1247	1326	3173	237	762	283	2124	1297	929	234	1096	850	312	868	35277	381	616	605	316	174	605	4092	33	89	4472	2817	697	36250
2010	Hepatitis B	31548	3172	17317	49173	53411	126699	41418	28983	6688	50062	16390	137445	69198	38317	26474	12141	31817	18192	26323	1060582	5435	14909	27405	28274	4654	40903	56327	2164	490	40284	21131	33838	1193266
2010	Hepatitis C	2690	1514	1995	2091	7590	15035	9564	2796	905	4476	5391	26931	5009	5838	5579	2090	1630	8316	6356	153039	454	1463	4222	1580	363	5269	6942	396	20	8892	5381	2261	163174
2010	Hepatitis E	1576	437	403	843	104	2921	832	207	54	846	539	454	1866	701	111	3634	531	272	1281	23682	28	45	255	1288	585	178	732	200	0	198	448	2113	24260
2010	Infectious diarrhea	47629	47661	19941	21632	7246	117098	22902	6650	3281	47007	3949	31130	19462	23249	1865	10046	24997	1997	16815	746551	7400	2375	20035	31354	4651	12947	37080	33971	35	15880	7880	98386	751230
2010	JE	187	0	165	29	34	49	55	232	14	15	2	433	112	125	1	55	32	0	0	2541	2	1	107	201	6	61	305	0	0	1	252	65	2738

2010	Malaria	1860	36	35	67	10	95	65	397	73	41	7	887	429	163	2	362	29	13	28	7389	1	1	40	112	36	5	312	9	32	6	2115	121	7759
2010	Mumps	12329	2906	14575	8586	5399	29497	20025	8537	6291	11376	3648	15337	22678	19417	1878	5557	9123	4463	11076	298932	5429	1328	8210	6368	3567	5038	13714	2142	443	5064	13850	21081	299397
2010	Pertussis	38	14	15	2	79	17	4	23	0	389	58	79	16	26	5	40	7	17	5	1764	13	3	56	235	0	60	200	122	0	167	33	41	1838
2010	Rubella	1788	1089	610	1704	1388	1159	1009	538	28	3425	445	1439	1538	2143	931	952	747	709	8483	43117	657	713	1283	521	279	259	2101	824	1740	852	944	2819	44490
2010	Scarlet fever	117	1553	205	120	359	243	161	378	0	1419	3135	539	216	170	1247	705	26	1093	2567	20876	347	199	634	718	413	816	886	623	41	1046	445	455	20774
2010	Syphilis	10188	4382	8457	17676	4039	40410	37216	5991	2382	3214	7910	13560	9218	15403	5856	24684	5728	5679	12509	358534	1478	1553	4617	6536	14680	6889	20073	2995	221	10831	5003	49156	375309
2010	Tuberculosis	39539	8021	25702	21375	23888	95911	47110	49049	9475	41559	36667	69364	48479	56753	17941	41663	37711	22954	25958	991350	3588	4868	25615	40234	6595	23849	66902	3137	3432	35501	25417	33093	1392210
2010	Typhus	16	0	0	37	1	133	72	0	54	346	9	9	0	18	1	0	1	0	27	2235	0	0	10	67	0	35	83	8	0	0	1303	5	2264
2011	AHC	2371	186	1229	950	322	3312	4138	635	1112	1539	132	1006	2378	2323	288	1246	1476	162	84	34262	197	46	1407	506	95	337	1665	96	6	57	1244	3717	34570
2011	AIDS	452	231	734	263	64	1469	5006	472	59	154	158	1752	448	925	33	462	318	184	184	20450	12	30	169	122	356	196	2156	114	5	693	2719	510	41812
2011	Brucellosis	19	40	1	16	50	52	0	6	0	3149	5178	1225	11	3	17320	17	1	2063	853	38151	471	39	556	449	4	5135	8	97	0	1314	16	58	43827
2011	Dengue fever	1	4	0	12	2	49	3	0	3	1	1	0	3	7	0	4	2	0	0	120	0	0	1	0	1	0	3	1	0	0	13	9	124
2011	Dysentery	14973	18525	8713	1047	10983	5016	5409	7286	596	16568	5462	17252	11404	9136	2795	7404	7135	4040	7127	237930	2182	952	9559	12168	1354	6218	13116	8475	1252	9005	6995	5783	240557
2011	Echinococcosis	1	2	1	1	503	2	0	0	0	3	3	6	1	1	118	8	1	1	2	2909	306	361	17	2	1	5	223	1	11	1318	9	1	3421
2011	Enteric fever	192	20	76	319	53	1461	955	915	19	200	27	165	233	860	20	270	342	12	107	11798	15	19	33	55	37	293	226	4	0	253	3948	669	12217
2011	Gonorrhea	2898	1238	1990	5120	1567	17230	6060	1523	774	918	1433	2014	2416	2461	1991	6512	2957	1849	2262	97954	399	119	1451	2584	5049	1141	3990	469	53	2126	1720	15640	100004
2011	HFMD	84829	30843	20085	60348	6972	273902	141961	23620	50716	71935	15760	59655	63585	102446	23234	114825	38382	19911	19958	1619706	5899	602	31652	95843	37131	30390	40411	22280	272	6047	39059	87153	1638743
2011	HFRS	134	21	13	259	5	336	9	94	1	513	1576	148	282	634	80	229	547	679	981	10779	0	0	2605	958	4	39	59	13	0	1	19	540	11323
2011	Hepatitis A	1071	115	1164	736	3550	1406	1056	1038	213	576	334	2462	1298	969	302	926	639	254	1013	31456	289	739	591	423	189	829	3553	12	81	3077	1792	759	32659
2011	Hepatitis B	33379	3116	17723	46524	53098	137084	44099	21611	7346	52550	16549	149852	67259	43193	26892	13068	33828	18012	26752	1093335	5925	13118	26558	31026	4662	43812	55314	2075	872	45142	22442	30454	1252236
2011	Hepatitis C	3305	1278	2544	2233	8445	17288	10050	3084	1117	5190	5604	32427	6092	7428	5332	2062	1915	9078	6408	173872	589	1558	4823	2104	389	6099	7642	418	27	11013	5910	2420	188807
2011	Hepatitis E	2135	463	611	1206	128	3122	1124	485	79	1054	558	689	2094	1009	118	4091	677	319	1505	29202	39	63	336	1424	621	411	1136	174	3	247	663	2618	30459
2011	Infectious diarrhea	58483	49619	24212	21244	9928	132880	28068	8025	3020	49710	5682	35451	24021	25752	2724	14395	27734	2340	18377	836591	8328	2837	20897	39458	3901	13060	37540	31614	66	15521	10447	111257	841115
2011	JE	49	0	99	28	21	65	51	217	11	3	0	135	12	167	0	15	22	1	0	1625	0	0	29	33	11	15	318	0	0	0	262	61	1887
2011	Malaria	644	37	32	68	21	96	116	153	9	50	16	314	167	152	7	372	41	12	46	4088	3	4	35	118	38	9	197	14	3	5	1194	115	4552
2011	Mumps	17606	3467	17061	15927	13083	54472	28654	12486	4582	25379	5616	20572	26363	19175	4908	8893	14339	5870	20523	454385	4494	1301	20786	12880	3269	14311	22889	2448	1862	11109	20637	19423	458232
2011	Pertussis	94	8	21	1	45	119	6	67	2	170	27	122	32	64	2	70	10	6	3	2517	0	2	104	282	2	49	382	74	3	581	51	118	2591
2011	Rubella	3044	839	3218	2484	773	3928	4452	2610	128	2649	345	1176	1403	2181	480	1333	1139	912	10830	65549	398	974	4614	383	398	753	4489	2025	113	1257	1937	4284	67887
2011	Scarlet fever	461	5523	403	339	667	1188	444	542	4	4062	6890	1421	597	598	3188	1726	48	4343	6300	58638	859	231	1026	3556	3109	2833	1789	1246	65	2458	952	1770	64358
2011	Syphilis	12234	4671	9708	18433	4954	46742	40067	6225	2415	4612	8787	18431	9319	18595	7123	23635	6377	8082	15920	395182	2264	1618	5571	7643	14137	8112	21479	2951	207	15333	6023	43514	419306
2011	Tuberculosis	37315	8099	25622	19399	22529	86913	45524	46378	9285	38744	34173	65741	50430	56986	16443	38301	35510	21404	30138	953275	3039	5232	25313	36556	6994	23149	63404	3453	3690	34432	26665	32414	1357922
2011	Typhus	48	1	0	29	2	161	73	0	47	258	5	24	0	15	0	1	0	0	34	2360	1	0	12	105	0	32	126	11	0	1	1372	2	2393
2012	AHC	2141	158	1514	603	572	3728	4842	695	689	1488	87	1527	2528	1701	189	935	846	382	199	32333	189	58	2125	517	47	487	1489	58	1	35	1023	1480	32530

2012	AIDS	815	556	1618	456	141	3084	8432	1396	130	278	259	3193	853	1925	69	978	568	254	449	41929	48	77	340	289	652	252	4902	156	21	2450	6312	976	49852
2012	Brucellosis	27	62	1	17	118	59	1	11	2	4066	7459	1926	11	21	12017	12	9	1956	1475	39515	449	16	596	665	3	6130	3	111	0	2164	38	90	42267
2012	Dengue fever	0	12	4	18	1	474	2	0	2	2	1	3	3	6	0	6	4	0	0	575	0	0	1	0	0	0	3	0	0	0	24	9	577
2012	Dysentery	14363	13186	8226	913	9229	5012	4933	6025	699	14336	4555	18291	8352	8281	2355	5785	6602	3211	6140	207429	1855	805	9973	8870	713	5405	10542	8637	1276	7293	7820	3746	210006
2012	Echinococcosis	0	2	1	2	414	0	0	1	1	4	2	6	0	0	90	1	0	0	0	3234	361	369	11	2	1	2	401	0	34	1525	1	3	3591
2012	Enteric fever	202	6	74	357	64	1444	1005	853	22	182	9	182	209	896	21	202	249	10	113	11998	10	18	26	49	33	321	222	8	0	261	4376	574	12452
2012	Gonorrhea	2387	1115	1759	5166	891	17615	5222	1224	760	951	1539	2186	2099	2293	1916	5357	2719	1696	2464	91853	322	145	1578	2671	4711	1185	3313	459	43	2236	1893	13938	93608
2012	HFMD	107738	38528	28299	91793	14312	330664	231984	56511	47715	82582	13676	68855	92422	189507	16496	115680	56385	12011	43808	2168737	7285	792	57808	88844	50907	31321	51794	20705	1304	9302	61868	147841	2198442
2012	HFRS	189	18	12	373	30	339	7	94	0	693	1717	186	224	657	72	253	607	745	1126	13308	2	0	3591	1696	1	52	48	28	0	1	49	498	13918
2012	Hepatitis A	956	82	850	640	1867	1370	723	595	121	485	278	1880	994	674	220	697	474	212	758	24453	169	685	478	378	154	776	3436	17	172	2346	1351	615	25452
2012	Hepatitis B	36659	2612	18612	50058	30722	142241	44952	20551	8215	56785	15451	131568	63260	50447	28170	12089	35387	17115	26275	1087086	6716	13456	28193	37923	12556	53117	51591	2039	981	45726	25490	18129	1257320
2012	Hepatitis C	4236	1057	2799	2719	8957	20850	10130	3134	1413	6389	6417	39887	6350	9513	6804	2423	2202	8597	6807	201622	631	1811	5981	2845	1606	7576	8730	382	30	11346	6927	3073	219110
2012	Hepatitis E	1762	351	543	1236	135	3066	1266	435	91	940	473	677	2040	1256	118	3516	514	273	1370	27271	49	95	265	1332	492	646	939	159	1	289	843	2099	28172
2012	Infectious diarrhea	65324	46144	27094	24678	11193	125387	30215	8973	3154	51424	5279	45104	29763	25039	2562	15168	30478	3557	20105	885678	9007	4165	26696	47577	4222	16528	33011	40305	49	14595	12859	106023	896808
2012	JE	84	0	176	9	6	61	71	156	11	5	0	147	10	152	0	28	28	0	0	1763	0	0	64	50	8	18	328	0	0	0	315	36	1934
2012	Malaria	99	30	23	54	7	87	218	15	13	35	7	157	127	164	4	198	53	13	43	2451	2	2	31	93	17	2	158	9	8	7	636	139	2820
2012	Mumps	27703	3778	13388	13886	15873	49176	25899	14044	4327	21470	6141	22835	25903	18764	10949	19131	14378	6961	12981	479518	6439	1067	26826	24408	3402	11482	26960	2394	2149	12744	14318	19742	485450
2012	Pertussis	76	15	32	6	46	69	3	59	1	120	17	59	24	25	0	52	8	1	1	2183	4	1	80	147	3	53	260	95	0	767	53	106	2286
2012	Rubella	1829	541	2794	802	77	2256	1118	1335	360	653	794	567	996	2795	624	753	1233	1542	2649	40156	825	420	1113	210	835	1928	1738	630	24	1665	4602	2448	41507
2012	Scarlet fever	442	3175	447	264	1212	1230	259	606	1	2578	3568	972	512	445	2528	1089	28	2806	4188	46459	879	163	1439	3959	2093	3151	1744	1120	115	2215	1525	1706	47430
2012	Syphilis	14382	4438	10708	19927	4616	48279	27802	7761	3080	6207	9321	24257	9452	20938	9011	22938	7077	7456	17243	410074	2716	2482	6917	10657	13864	10367	22670	3129	222	19323	8666	34168	434280
2012	Tuberculosis	38669	8219	24977	18831	19410	82010	47031	45546	9359	38303	34083	68195	50483	59772	16723	37405	34221	19246	30908	951508	2941	6369	24046	36908	6966	20989	62731	3307	4100	40014	27592	32154	1371488
2012	Typhus	31	0	0	34	4	162	79	0	27	232	0	7	2	18	5	0	0	0	59	2102	0	0	16	89	0	10	103	11	0	0	1208	5	2119
2013	AHC	2752	167	1435	571	593	4487	6257	868	1072	1651	55	1708	3319	1577	184	730	1151	237	139	36387	150	50	2057	679	31	493	1163	40	2	29	1329	1411	36558
2013	AIDS	841	610	1815	412	174	3066	7040	1545	151	414	376	2852	1021	2096	135	1173	755	345	576	42286	59	93	498	350	549	312	5972	194	9	1791	5812	1250	44491
2013	Brucellosis	18	85	4	45	488	108	21	26	2	5103	7056	3256	45	20	8911	43	10	2024	2053	43486	883	100	795	1278	2	6895	7	166	2	3909	45	86	46089
2013	Dengue fever	2	16	12	33	1	2894	8	1	0	10	1	36	10	6	1	13	7	2	3	4663	0	0	6	4	2	0	18	1	0	1	1538	37	4683
2013	Dysentery	12495	11312	9191	803	8207	4235	4219	5506	607	12388	4130	17596	6700	7955	2168	4545	6320	2091	5744	188669	1722	853	8820	7960	385	5472	9472	9118	878	7839	6870	3068	190601
2013	Echinococcosis	4	4	0	2	373	0	1	0	0	3	0	5	1	2	68	6	0	2	1	3841	331	606	13	5	2	5	844	0	56	1502	4	1	4241
2013	Enteric fever	264	15	116	459	43	1689	1166	921	28	220	21	159	382	1156	38	216	277	11	169	14136	22	14	34	64	31	545	233	7	0	389	4844	603	14629
2013	Gonorrhea	2877	1154	1827	5792	1037	20169	4816	1321	1123	953	1622	2705	2061	2410	1910	5829	3053	1868	2717	99659	355	153	1576	3499	5092	1313	3000	456	48	2098	1643	15182	101108
2013	HFMD	93279	33763	29270	76475	7593	357901	210998	43108	50231	56761	7133	61826	65888	108202	16535	96391	32125	7572	33211	1828377	6094	1682	52610	72211	41921	24167	54597	13578	543	5804	58376	108532	1855747
2013	HFRS	193	27	16	446	14	416	8	112	2	1384	1887	150	254	825	113	349	691	726	1301	12810	2	0	1392	1779	3	20	49	31	0	0	99	521	13568

2013	Hepatitis A	796	88	888	497	829	1414	665	358	111	503	225	943	966	636	299	553	337	164	1054	22244	92	613	483	400	128	936	3380	37	243	2764	1370	472	22901
2013	Hepatitis B	34752	1944	19190	47247	11862	139767	42062	20190	10889	56248	13675	75256	59920	53147	24661	10878	35093	12996	20747	962974	4915	13249	25083	43615	9843	49608	42627	1713	1239	42545	24051	13962	1114319
2013	Hepatitis C	4739	979	3179	2707	7501	20546	9941	3857	2021	7132	6380	34371	7350	11622	7044	2794	2513	6669	6570	203155	558	2133	6219	3569	1562	8526	8901	376	19	12360	8615	2402	222094
2013	Hepatitis E	1895	391	855	1187	154	2647	1422	423	92	883	448	710	2211	1342	189	3899	574	293	1087	27902	27	88	287	1610	450	708	935	121	1	308	832	1833	28991
2013	Infectious diarrhea	74658	46053	35684	31503	11121	166274	36194	9189	3774	56022	5934	53673	35036	30252	2934	15887	37043	2318	20511	1012589	8869	4213	26809	50890	5409	19312	29002	43134	37	15874	16041	118939	1017492
2013	JE	41	13	127	13	34	51	15	97	2	234	0	109	15	80	2	28	11	0	0	2178	0	0	153	409	2	98	368	18	0	1	184	73	2320
2013	Malaria	194	42	36	51	17	106	1255	13	15	54	15	193	131	213	9	344	49	12	45	3896	5	5	41	130	22	19	244	13	4	6	426	187	4242
2013	Mumps	23895	3076	13188	5156	12013	26593	15221	7794	3745	11673	5115	16485	18670	16590	6930	19281	9028	5130	5226	327759	5656	1206	13548	23677	3297	5905	15316	2099	313	9665	8617	13651	332349
2013	Pertussis	37	15	59	3	43	60	2	25	0	175	45	23	19	16	6	31	8	8	4	1712	0	1	77	265	0	30	235	169	0	290	23	43	1753
2013	Rubella	856	187	611	488	109	1230	789	388	93	611	142	352	191	1866	253	212	768	505	506	17580	155	164	593	308	65	960	718	799	17	393	2611	640	18569
2013	Scarlet fever	377	2048	341	240	1162	1434	240	482	5	1770	2214	783	378	367	1851	1423	93	1558	2181	34207	1072	245	1207	2861	1771	1608	1373	746	48	1747	1365	1217	34519
2013	Syphilis	16182	5137	11364	21651	3517	48228	12480	8394	3499	7432	9836	23736	9408	20850	10791	21700	8706	7058	19437	406772	3565	3150	7543	12792	13260	10158	19941	3345	469	21780	9542	31821	435676
2013	Tuberculosis	37664	7428	24536	17697	17851	77509	45405	46783	8446	35917	33831	63074	48045	59090	14408	34951	32490	17062	26181	904434	2886	6055	22869	35971	6965	18999	60855	3148	4254	38567	26470	29027	1298123
2013	Typhus	28	2	1	33	1	141	126	2	32	163	3	8	0	17	2	0	0	0	35	1989	0	0	29	106	0	23	105	5	0	1	1124	2	2021
2014	AHC	2982	92	2482	735	418	4661	7679	945	1271	1772	40	1801	3072	1829	119	590	999	40	99	41514	119	109	1599	667	39	409	959	20	2	44	4732	1189	41741
2014	AIDS	861	704	2491	622	228	3498	6615	1705	102	515	485	2868	1098	2276	202	1312	782	437	654	45145	57	107	531	453	454	430	6503	233	21	1612	5784	1505	47215
2014	Brucellosis	76	144	10	55	1393	188	20	56	11	6519	5625	5175	196	50	10135	90	32	1809	2797	57222	2063	46	1473	2803	2	8540	25	181	0	7478	131	99	59981
2014	Dengue fever	5	9	24	284	3	45189	854	2	15	5	2	27	38	76	1	10	15	1	7	46864	0	0	4	10	8	1	23	0	0	1	213	37	47331
2014	Dysentery	9407	10617	7918	685	7130	3615	3198	3508	413	10865	3257	14388	5561	5254	1746	3984	6160	1471	4771	153585	1410	844	6316	6948	207	4347	7168	7625	843	5893	5642	2394	155247
2014	Echinococcosis	6	3	0	0	350	0	1	1	0	1	4	10	1	0	84	2	0	0	0	3363	276	509	10	1	0	1	543	0	111	1440	8	1	4017
2014	Enteric fever	251	19	109	550	46	1509	1013	708	27	271	47	151	328	1011	27	160	238	10	270	13768	15	12	35	65	27	837	194	16	1	173	5126	522	14159
2014	Gonorrhea	2779	1081	1939	4754	1000	16344	4204	1300	1431	1223	1532	3026	2105	2162	2101	5896	2894	1644	2703	95473	316	150	1268	3795	5661	1492	2713	409	47	2121	2104	15279	96874
2014	HFMD	149785	47425	54347	111446	13179	429483	334989	50882	33561	85223	13832	129087	97270	212792	17579	167143	60460	20515	33684	2778861	5765	2678	70215	118987	64688	34594	95557	20000	1078	7451	82630	212536	2819581
2014	HFRS	159	15	21	447	29	465	13	83	4	966	1846	133	238	584	207	351	517	753	1317	11522	2	2	1039	1576	5	14	67	38	0	1	246	384	12194
2014	Hepatitis A	720	143	1008	544	675	1623	870	437	92	624	242	741	936	648	296	671	302	184	1706	25969	137	426	403	659	197	1229	2622	70	183	5925	1146	510	26740
2014	Hepatitis B	33930	1648	18476	46577	10511	142723	41122	19947	11594	53177	11439	64429	58178	56312	22566	11427	35167	12710	20656	935702	5028	8509	25886	47439	8551	48518	38686	1702	1733	37727	25600	13734	1084543
2014	Hepatitis C	5395	909	3400	2789	6681	21279	9560	4448	2694	8023	5145	27959	7896	12826	6781	2734	3462	6320	8081	202803	650	1995	6832	4000	1760	8758	8886	459	30	11715	8889	2447	222528
2014	Hepatitis E	1764	330	982	874	128	2820	1552	544	142	780	384	675	2205	1278	150	3428	535	261	1063	26988	29	85	232	1358	473	693	1033	109	4	386	858	1833	27943
2014	Infectious diarrhea	70725	42823	30123	25224	10243	112033	31576	8297	4121	51939	5419	50543	30733	26261	3242	14809	30835	1225	16037	867545	8222	3462	22667	47947	5326	16191	24747	39017	47	15106	15982	102623	871085
2014	JE	29	1	49	20	25	33	21	64	2	19	0	47	16	57	0	6	12	0	0	858	0	1	54	24	1	8	157	0	0	0	189	23	1044
2014	Malaria	153	37	24	63	68	114	183	29	12	55	5	222	136	157	2	358	44	25	39	2921	4	2	58	153	22	13	273	12	7	4	444	203	3123
2014	Mumps	10920	2507	8710	3558	4804	16006	8519	5185	1811	7736	2815	12726	8792	11333	2613	8149	5560	3551	3602	187500	1970	1792	9049	8092	2704	4293	10186	1640	152	4697	5106	8922	189469
2014	Pertussis	64	84	166	9	48	248	3	32	0	359	55	115	39	48	7	47	16	4	3	3408	2	2	188	1058	0	65	157	269	1	208	21	90	3426

2014	Rubella	271	246	462	138	146	1495	270	117	82	727	62	235	56	799	186	116	431	238	782	11793	111	116	177	410	241	469	314	314	5	359	2094	324	13305
2014	Scarlet fever	549	3918	401	362	922	2168	344	519	7	3224	3771	1272	589	735	3513	2356	108	3763	4707	54247	908	182	890	5136	3574	2255	1211	1718	54	2112	1126	1853	54541
2014	Syphilis	17944	5646	11992	23447	3650	50843	9843	9632	3445	8508	9954	17964	10368	20839	11314	23917	9882	6266	19594	419091	3531	3342	8548	14243	13370	10231	19491	3186	709	21159	12976	33257	441818
2014	Tuberculosis	37290	7271	22756	17809	16642	81498	47497	45314	8686	35304	34296	62596	46150	57458	12273	33264	32474	16688	25026	889381	3326	5845	21601	34502	6940	16817	56489	3132	4618	39851	26251	29717	1190246
2014	Typhus	24	2	3	21	5	106	111	1	16	181	6	6	4	27	2	0	1	0	37	1670	0	0	36	146	0	14	119	2	0	0	795	5	1703
2015	AHC	3151	65	1155	452	452	3662	6192	998	684	1782	22	2096	3218	1200	87	404	976	27	114	34576	125	95	1547	793	47	356	747	25	63	26	2975	1040	34665
2015	AIDS	1040	777	2621	760	310	3919	6300	2112	190	681	552	3077	1173	2575	207	1576	1179	540	825	50330	78	151	655	608	522	493	7771	272	33	1868	5802	1663	51556
2015	Brucellosis	76	281	48	104	2303	484	39	68	3	5526	5960	5573	257	95	7238	79	45	1625	2922	56989	2888	16	1221	3691	3	6997	44	265	1	8820	219	98	60782
2015	Dengue fever	7	18	19	89	1	1683	6	3	3	9	1	9	11	23	1	6	4	0	6	3858	0	0	7	28	16	0	30	1	0	1	1816	60	3884
2015	Dysentery	9789	9724	7353	580	6056	3213	3851	2947	407	9625	3265	14001	4667	4089	1656	3900	4708	1029	4254	138917	1300	784	4966	6305	194	3328	6004	8007	740	4954	4843	2378	140544
2015	Echinococcosis	1	3	0	0	330	1	1	0	0	1	6	9	2	1	63	5	0	0	0	3418	241	497	15	5	0	4	682	0	170	1370	11	0	3913
2015	Enteric fever	295	31	151	483	40	1590	1144	610	34	298	38	296	272	1033	26	156	231	19	167	11637	18	8	39	68	28	393	295	20	1	201	3267	385	12220
2015	Gonorrhea	2823	1105	1775	4998	761	16989	4070	1669	1522	1270	1679	2755	2251	2398	2087	6090	3304	1457	2586	100245	337	167	1309	3769	7233	1358	2741	410	55	2049	3028	16200	101630
2015	HFMD	95683	28677	45829	86291	9167	373198	203486	34628	68726	52425	9271	86359	77245	135628	12217	95063	40248	6871	25521	1997371	9386	1727	68738	88859	39490	29854	66675	13327	2015	7890	82659	100218	2014999
2015	HFRS	159	12	6	416	33	420	15	46	3	616	1428	192	255	743	165	221	701	628	957	10314	1	1	1399	1121	2	14	101	32	0	0	264	363	10812
2015	Hepatitis A	604	108	877	539	749	1665	728	391	82	550	247	390	857	619	198	666	277	222	1951	22667	148	338	377	517	197	1111	2387	65	253	3798	1291	465	23418
2015	Hepatitis B	37275	1683	19753	49254	10047	144304	41421	20595	11531	54593	10230	60032	61902	54372	22028	14286	38715	11245	19703	934215	2912	8536	23170	47722	8426	45433	38573	1898	2500	37765	21331	12980	1085113
2015	Hepatitis C	6328	876	3914	2856	6791	21066	9118	4707	3351	9409	4894	24744	8736	13101	6894	3159	3779	6378	8803	207897	804	1992	7191	4133	1789	8505	9956	610	31	10955	10371	2656	232400
2015	Hepatitis E	1589	275	900	953	124	2739	1540	606	240	768	420	607	2325	1154	187	3034	825	288	969	27169	16	72	275	1200	688	601	1184	100	4	522	1226	1738	27986
2015	Infectious diarrhea	81947	40308	31670	24206	12050	119909	53965	8334	4506	57715	5987	57138	35331	26299	3837	15248	29378	1147	17913	937616	8582	3894	23288	60530	5120	17146	25977	36712	74	14791	18457	96157	943701
2015	JE	19	1	42	3	17	29	16	51	3	8	0	46	6	79	0	4	6	0	1	624	0	0	40	17	2	28	99	0	0	0	98	9	819
2015	Malaria	131	46	34	77	28	108	240	14	15	60	15	187	124	142	6	402	57	22	65	3116	7	1	75	215	25	18	292	17	8	3	516	166	3289
2015	Mumps	10772	2157	11113	3039	2750	16554	7825	6507	1457	7573	1610	18490	9023	13476	1839	5990	6465	1696	3009	182833	1393	1165	5856	7206	2639	4059	8629	1539	200	5786	5531	7485	184295
2015	Pertussis	24	93	370	13	89	657	5	42	0	356	40	225	129	92	14	33	18	1	4	6658	3	3	739	1398	4	108	290	376	1	1293	28	210	6819
2015	Rubella	835	245	326	66	201	389	151	123	26	199	105	141	90	325	96	515	93	33	273	8133	250	77	921	165	325	50	304	182	12	191	1146	278	9487
2015	Scarlet fever	1076	3861	816	658	1195	2788	446	800	13	4192	3707	2085	1143	977	2855	3267	66	2645	6388	68249	828	444	2333	6051	4679	2797	1921	1677	106	3111	1834	3490	68798
2015	Syphilis	20800	5310	14522	23963	4348	50019	8211	11356	4390	9966	9703	16075	12024	20910	10873	23594	11406	5874	17752	433974	3254	2489	9404	14680	13616	9813	22652	2938	1066	24710	15563	32693	458682
2015	Tuberculosis	35550	6879	22435	17057	14228	79485	45835	46817	8847	34046	33242	59199	45448	55919	11950	31456	32534	15537	23589	864015	2794	7191	21388	32361	6685	15439	54645	2961	4452	42413	25651	27982	1154156
2015	Typhus	71	0	6	16	4	121	105	0	13	259	6	4	5	24	3	0	0	0	30	1461	0	0	17	76	0	13	105	2	0	0	577	4	1520
2016	AHC	3083	36	1126	384	462	3981	6372	916	919	2124	26	2542	3676	1245	113	340	1016	19	102	34253	110	83	1310	847	16	268	659	16	85	27	1613	737	34429
2016	AIDS	1334	786	3077	933	402	4118	5986	2619	175	584	566	3007	1277	2809	287	1612	1373	549	1013	54360	84	175	809	730	549	531	9153	277	51	1921	5707	1866	55600
2016	Brucellosis	78	192	43	75	1745	380	286	113	5	3774	5342	3993	265	162	5970	143	65	1480	2338	47139	2160	15	948	3886	12	4587	113	182	10	8402	282	93	50678
2016	Dengue fever	2	9	12	976	0	544	5	5	2	4	0	12	10	17	0	16	3	1	8	2050	0	0	6	12	4	0	16	3	0	0	325	58	2076

2016	Dysentery	7550	8914	7137	487	6328	2786	3192	2824	310	8542	2572	11977	4144	3808	1435	3234	4066	826	4134	123283	1378	909	4427	4335	130	3265	5685	8149	666	3701	4198	2174	124379
2016	Echinococcosis	3	0	2	0	339	0	1	3	0	1	2	27	3	3	59	6	0	0	2	4777	238	780	14	4	0	2	1218	1	145	1915	9	0	5290
2016	Enteric fever	378	12	114	706	35	1522	1123	525	33	324	23	189	287	1018	51	317	306	14	143	10899	21	7	40	110	20	320	351	21	1	133	2281	474	11429
2016	Gonorrhea	3717	1429	2048	5921	841	21485	4905	2264	1854	1416	1530	2970	2772	3266	2237	7203	3651	1367	2564	115024	315	148	1718	4085	6770	1309	2917	318	45	1720	4098	18141	116306
2016	HFMD	142166	30240	64844	87840	9378	369052	284442	40045	33990	60775	8162	128039	120470	185515	13984	157594	47944	15673	23686	2442138	3096	1132	52887	95129	57315	17546	92594	13904	1695	11268	85711	186022	2468174
2016	HFRS	207	9	13	366	19	410	5	59	3	434	1200	176	236	588	106	330	676	515	863	8853	4	0	933	985	4	8	110	31	1	1	216	345	9282
2016	Hepatitis A	501	141	886	451	681	1607	563	275	62	398	290	227	832	569	209	601	273	238	2968	21285	170	328	295	373	234	953	1979	61	77	3576	992	475	21866
2016	Hepatitis B	42729	1696	20373	44008	9897	151255	43676	17594	10495	57835	9336	61107	63549	55445	20672	14140	40795	8435	19371	942268	2589	9557	20975	54732	10054	42355	38688	1854	1927	39491	13769	13869	1100691
2016	Hepatitis C	6610	779	4292	2652	7091	22889	9348	4834	3217	8680	4259	22647	9585	13364	7030	3229	3108	5343	8048	206832	828	2290	7586	4799	2311	8060	9953	553	53	10858	9835	2701	231725
2016	Hepatitis E	1435	244	748	834	166	3115	1589	555	416	747	366	637	2724	1173	142	2748	706	218	1471	27922	18	116	274	1060	713	662	1183	99	3	436	1427	1897	28671
2016	Infectious diarrhea	90686	39154	36475	25259	14757	120986	84398	9331	4259	51672	4704	60647	42472	29673	4263	15414	32271	976	20897	1018605	9128	4571	24160	73834	7180	15260	27454	32599	69	14391	18976	102689	1023172
2016	JE	33	0	46	0	83	35	9	65	2	72	0	243	8	45	1	14	5	0	0	1237	0	0	120	116	1	102	118	0	0	1	103	15	1363
2016	Malaria	146	32	38	114	23	146	304	36	8	89	7	205	148	147	6	306	49	17	54	3147	8	1	82	247	24	15	339	11	0	6	331	208	3341
2016	Mumps	7721	2172	7301	2940	2408	17420	8919	6152	1239	7362	1206	15852	10402	15772	1845	5038	7606	1121	3016	175001	1421	487	6097	6361	2379	3731	8703	1430	546	5653	5667	7034	176083
2016	Pertussis	25	91	613	3	120	584	4	62	0	365	28	115	53	81	9	14	53	2	1	5584	3	3	594	1400	5	109	354	313	1	392	42	145	5683
2016	Rubella	281	115	152	131	111	303	34	52	56	122	47	290	273	130	65	173	56	25	425	4535	9	20	637	113	52	67	178	68	16	279	146	109	5474
2016	Scarlet fever	814	2621	484	609	1429	2599	525	725	6	3869	2832	1481	1011	777	2600	2235	46	2114	4542	59282	970	382	3060	7109	2922	2819	1719	1734	79	2995	1701	2473	59581
2016	Syphilis	22819	4975	16550	22461	4837	52863	7391	10718	4617	9561	9265	14638	12678	22653	9572	23688	13105	5054	16393	438199	3787	2852	9896	15305	13783	9270	23259	2568	1142	21016	17051	34432	464457
2016	Tuberculosis	34878	6731	22123	16409	15110	77920	41375	46117	7667	33663	30557	57006	43712	51157	12129	28655	32866	13700	22524	836236	2674	7573	21355	30311	6589	14160	53865	3271	5001	43816	26304	27018	1121019
2016	Typhus	44	1	6	18	5	164	109	0	32	124	0	4	40	23	4	0	1	0	21	1160	0	0	27	104	0	7	108	0	0	0	317	1	1188
2017	AHC	3115	54	1468	367	716	4417	5749	1119	1524	2094	33	3016	2994	1412	116	324	1020	10	66	34652	99	113	794	856	18	313	696	10	42	36	1424	637	34666
2017	AIDS	1149	746	2966	968	481	4308	6108	3045	169	752	617	2997	1399	3013	311	1317	1435	554	1100	57194	101	195	888	848	515	638	10777	281	28	2003	5673	1812	58964
2017	Brucellosis	146	153	43	92	1642	386	194	104	29	2695	4231	2447	235	179	7347	187	73	1159	1915	38554	1696	24	731	3094	5	3071	73	148	18	6073	221	143	41241
2017	Dengue fever	17	22	15	70	3	1662	10	3	4	11	1	21	11	26	1	28	18	1	6	5893	1	0	8	104	13	0	32	0	0	1	2575	1229	5930
2017	Dysentery	6360	8220	8171	370	4864	2129	2288	1538	204	7919	2447	11180	3498	2560	1164	2699	3339	745	3952	109368	969	774	4333	3845	81	2881	5558	8382	689	3065	3113	2031	110867
2017	Echinococcosis	6	0	5	1	379	0	2	5	0	2	0	12	1	2	56	5	0	0	1	5485	235	1189	17	7	0	9	776	0	273	2488	13	1	6910
2017	Enteric fever	303	21	120	711	38	1663	1115	548	46	291	29	185	320	1060	46	151	230	22	146	10791	25	12	43	65	25	364	377	21	1	137	2292	384	11301
2017	Gonorrhea	4427	1705	2555	7219	977	29946	6070	2768	2459	1600	1380	3424	3805	4138	2356	8749	4628	1209	2526	138855	410	224	2132	4072	5665	1554	3449	422	84	1573	5423	21906	140655
2017	HFMD	82736	19964	40587	88036	8594	408511	262174	32478	43635	45068	3324	71544	75622	147920	15447	91358	49821	5060	18393	1929550	7533	1822	39731	93523	23981	26076	62395	8509	974	3741	68577	82416	1952435
2017	HFRS	273	7	5	388	20	443	14	58	3	447	1119	435	505	632	93	375	571	574	1078	11262	2	0	2064	1252	2	26	260	32	1	3	229	351	11614
2017	Hepatitis A	458	174	835	556	677	1585	714	263	93	412	228	252	908	599	204	621	312	203	1303	18875	164	367	263	403	278	1165	2007	75	101	2098	1015	542	19603
2017	Hepatitis B	51432	1900	21266	41296	11042	164608	49221	20497	14865	58207	10819	66772	66150	60739	21351	12277	42421	7454	19991	1001952	2815	10632	17665	63258	10711	40009	39488	2148	2187	40800	14502	15429	1180545
2017	Hepatitis C	7546	843	4613	2525	7753	23927	9551	5470	3044	9374	4686	21446	10419	14241	7985	3290	2649	4415	9100	214023	803	2201	8049	5110	2328	8422	10230	784	58	10736	9395	3030	242897

2017	Hepatitis E	1623	319	949	725	137	3030	1472	674	471	773	303	601	2979	1294	132	2784	674	207	1536	29014	13	137	293	1151	859	598	1207	94	8	270	1599	2102	29844
2017	Infectious diarrhea	114103	42619	44395	34571	17876	163977	99052	12109	4803	61663	5399	74158	52533	32173	6202	25483	37292	1171	22765	1284644	10073	4404	33175	103434	6626	20546	35600	38009	132	17040	28419	134842	1280751
2017	JE	17	2	28	4	362	15	3	16	0	8	0	52	9	35	0	9	9	0	0	1147	5	0	151	89	2	53	180	2	0	1	82	13	1244
2017	Malaria	99	47	31	144	24	143	387	24	9	84	10	180	92	96	6	238	35	20	53	2679	4	0	61	197	34	20	212	7	1	7	236	178	2856
2017	Mumps	9003	2125	8946	2895	3069	25390	12004	10288	4133	12091	1259	30872	17104	31443	2360	5959	7500	1415	2908	252740	1288	1190	8619	6127	2172	7773	12435	1624	409	6949	6635	6755	254796
2017	Pertussis	67	116	1043	14	111	1110	3	156	1	674	36	123	188	136	17	61	172	7	3	10390	8	7	700	3729	89	196	468	660	1	155	38	301	10542
2017	Rubella	40	51	51	186	36	253	44	5	14	150	16	111	20	68	13	25	80	7	10	1605	6	17	18	25	3	61	60	21	6	79	42	87	2160
2017	Scarlet fever	1234	3643	554	1236	1454	6585	699	829	20	4610	2899	2082	879	1434	3217	2630	79	3042	5550	74369	1206	389	2830	8149	4237	2763	1994	2550	93	3263	1911	2308	74538
2017	Syphilis	25963	5193	19306	23932	5876	55777	6243	13151	5447	10060	9146	15209	13724	27475	10546	25019	16171	4429	16733	475860	3710	3381	10682	17806	13389	11028	27335	3164	1306	22013	16833	35813	505848
2017	Tuberculosis	34561	7114	23518	16576	14758	82117	42507	43426	7914	31329	27630	55317	40215	51893	12397	26812	33075	13747	23676	835193	2902	7958	22498	29987	6424	13702	52147	3266	5123	48581	27448	26575	1124707
2017	Typhus	38	0	5	19	9	123	70	0	22	60	2	7	27	32	5	2	0	0	24	929	0	0	26	99	0	4	100	4	0	0	246	5	963
2018	AHC	3253	38	1547	296	761	3405	8174	1060	2692	2507	16	2584	2761	1526	75	265	969	8	57	38250	159	92	625	1264	13	243	629	7	24	21	2606	573	38432
2018	AIDS	1290	848	3568	1049	618	4499	6020	3696	224	875	696	2898	1477	3099	343	1550	1641	590	1263	64170	115	155	1021	956	514	552	14500	286	52	2469	5531	1775	65779
2018	Brucellosis	150	123	23	92	1516	411	139	45	6	2955	4281	2015	110	176	9708	162	91	1206	2068	37947	1581	76	666	2790	8	2800	64	124	23	4197	234	107	40328
2018	Dengue fever	29	23	28	140	1	3315	40	2	30	12	8	33	29	175	0	65	14	1	11	5136	1	0	8	15	25	2	49	1	0	0	842	237	5141
2018	Dysentery	5783	6071	4279	318	4044	1743	1971	1172	162	6309	2144	9060	3130	2258	948	2470	2673	580	3439	91152	992	627	3571	3316	65	2436	4856	8578	939	2374	2944	1900	92885
2018	Echinococcosis	1	1	3	2	345	0	0	1	0	3	2	22	3	1	104	4	1	1	2	4327	295	1128	7	1	1	2	459	0	181	1747	10	0	5006
2018	Enteric fever	213	23	87	637	45	1915	1845	500	60	323	21	436	292	880	50	148	202	8	117	10843	30	5	49	68	8	351	365	28	0	174	1690	273	11543
2018	Gonorrhea	4476	1698	2622	6804	971	31262	6865	3280	2806	1171	1129	3195	4138	4139	1734	8979	4374	814	2072	133156	444	221	1991	3900	3812	1256	3156	378	90	1202	6297	17880	135429
2018	HFMD	119384	32658	92461	95260	13583	296239	173066	36694	25866	35622	10493	91995	92430	169212	11374	190694	59964	9797	22576	2353310	7165	3959	67099	101635	52573	19374	136975	16623	4267	11405	106208	246659	2375938
2018	HFRS	287	8	12	430	143	457	15	46	5	555	1249	398	923	714	150	270	705	578	1129	11966	5	2	1743	1218	2	21	291	20	1	1	253	335	12495
2018	Hepatitis A	534	145	663	382	677	1545	628	289	64	431	205	191	834	500	196	554	287	137	876	16196	125	318	259	399	256	1135	1812	33	114	1362	825	420	16736
2018	Hepatitis B	55959	2157	13679	42450	11921	137085	55726	20268	15262	58908	11194	70565	66757	70447	20194	12530	42012	7087	19506	999985	3375	9307	15428	74826	11635	41734	38546	2239	2995	36609	15041	14543	1225877
2018	Hepatitis C	8699	868	4158	2349	7738	23788	9599	5489	2384	10180	4534	21889	10926	16302	7851	3422	2670	4143	9233	219375	956	2096	8123	5896	2098	8596	10897	842	70	10066	10780	2733	251246
2018	Hepatitis E	1658	375	939	794	210	2910	1420	627	542	781	330	718	2994	1256	145	2858	629	214	1057	28603	56	185	314	1195	919	406	1213	107	6	324	1567	1854	29435
2018	Infectious diarrhea	101372	36349	48205	31169	18208	186300	122188	14262	5297	59554	4356	68911	47903	30795	5778	22327	35227	1064	20699	1282270	10836	3546	27074	123094	5527	20501	37122	35543	157	13367	37460	108079	1302900
2018	JE	35	33	44	4	503	20	9	50	0	50	0	99	8	45	10	5	3	0	69	1800	162	0	218	111	1	72	146	5	0	0	82	16	1934
2018	Malaria	102	45	29	109	19	146	250	26	9	77	16	186	128	122	7	238	42	12	58	2497	3	4	89	216	21	18	219	7	0	7	167	125	2690
2018	Mumps	15974	1962	6615	3036	5494	23098	15990	9758	8023	13985	1824	20029	15526	30433	2355	7877	6655	1587	3077	259071	1223	2090	8373	6926	2265	6206	15117	1100	1068	4755	10993	5657	261493
2018	Pertussis	212	188	2169	146	291	2480	176	540	27	904	42	304	273	1986	48	136	259	26	4	22057	17	5	2378	5770	113	183	1293	821	1	403	91	771	22466
2018	Rubella	100	58	54	364	23	798	1116	117	138	30	9	20	40	197	15	44	24	9	16	3930	3	13	61	34	21	27	192	19	8	29	235	116	4393
2018	Scarlet fever	1348	3626	734	1297	1624	5500	818	509	29	5316	2707	2418	1776	1778	3051	4026	98	2932	5082	78864	1224	500	3105	8949	4427	2584	1838	3183	82	3608	2388	2307	79845
2018	Syphilis	25577	5557	18243	24358	6504	56180	10053	15095	5772	10810	8811	17361	15204	32043	11153	27256	17385	4697	16007	494867	3632	3895	10799	18923	12002	12360	29185	2857	1846	23602	16651	31049	535655

2018	Tuberculosis	31949	6606	22561	17693	10163	72385	40457	40833	8524	29556	24713	52728	37470	54023	13678	24906	32739	12890	24384	823342	2504	8397	21442	28134	6321	12968	47601	3330	5619	74549	28618	25601	1110659
2018	Typhus	68	0	2	11	15	133	69	2	9	66	1	4	54	31	3	1	1	0	10	971	0	0	20	117	0	6	96	0	0	0	251	1	992
2019	AHC	3893	26	2249	344	656	4307	9347	1003	1638	2649	23	2665	3170	1555	153	291	1064	16	52	41439	163	65	666	1166	15	193	716	3	112	12	2550	677	41701
2019	AIDS	1264	682	3902	1165	605	4554	7032	4753	203	990	738	3298	1546	3179	346	1663	1750	616	1179	71204	146	200	1105	1041	536	615	17869	246	31	2362	5696	1892	72630
2019	Brucellosis	136	86	45	146	1711	436	151	25	14	3236	4229	2212	73	205	13780	139	61	1106	2283	44036	2199	128	1100	2479	2	3279	115	132	50	4060	310	108	46700
2019	Dengue fever	89	48	1411	1617	13	6042	1748	58	367	54	22	285	226	746	1	199	1282	12	36	22188	6	1	29	63	95	12	350	11	0	0	6471	894	22317
2019	Dysentery	4814	5835	3665	270	3491	1579	1758	1197	192	5444	1871	7858	2541	1614	832	2169	2172	508	2699	81075	560	553	2724	2850	67	2118	5195	8382	1240	1976	3141	1760	81781
2019	Echinococcosis	7	3	0	2	303	1	0	2	0	5	6	18	3	4	72	1	0	0	3	4003	249	1126	9	6	1	2	360	0	162	1644	13	1	4850
2019	Enteric fever	211	8	92	712	23	1267	1326	486	94	325	24	108	278	1206	30	131	160	13	82	9274	9	6	49	66	12	289	350	73	0	107	1541	196	9787
2019	Gonorrhea	3828	1457	2723	5835	984	28010	6571	3125	2046	1294	984	3429	2994	3962	1599	6962	3620	638	1816	117938	248	247	1530	4018	3144	1184	2880	416	95	1171	6857	14271	120146
2019	HFMD	73339	17357	63880	51274	18355	418335	196795	31452	44736	38245	7048	67040	93639	97110	15651	105188	33419	8780	15901	1918830	11687	2897	40652	77034	24489	17578	97579	9641	1409	8476	103378	126466	1944036
2019	HFRS	253	10	11	369	320	288	16	39	3	487	1320	229	460	678	168	283	652	473	858	9596	10	3	1164	749	1	17	90	13	2	0	269	361	10117
2019	Hepatitis A	518	110	713	454	745	1620	786	262	66	452	268	211	834	582	232	731	293	251	2483	19271	147	377	250	481	377	1486	1886	59	101	1053	933	510	20005
2019	Hepatitis B	67445	1877	9919	43002	11910	145766	53055	17962	15034	58176	11281	60554	65424	73470	18268	13159	41800	6480	18957	1002292	2830	9690	14535	78500	12978	40892	41194	2250	3855	33268	14536	14225	1247092
2019	Hepatitis C	10574	717	4205	2095	7394	25761	9257	5189	2186	9800	4630	21489	10333	17318	7082	4465	2622	4135	8958	223660	853	1866	8307	6016	2307	8984	12996	923	83	8962	11022	3131	260704
2019	Hepatitis E	1452	278	1042	687	188	2738	1512	577	509	769	291	597	2624	1069	158	2855	679	186	1033	28155	32	121	281	1159	886	659	1602	111	3	407	1815	1835	29126
2019	Infectious diarrhea	117650	37088	41619	25090	18533	169956	120385	15370	5970	61777	4737	74215	49391	28938	6412	26945	34596	1070	17843	1335627	8129	4353	25442	150065	5861	23289	39269	35569	196	14188	50140	121541	1344396
2019	JE	3	0	20	1	50	9	8	22	0	2	2	10	7	23	1	0	2	0	0	416	3	0	33	23	1	12	86	0	0	1	91	6	463
2019	Malaria	102	38	32	113	22	155	183	28	2	91	18	232	151	115	9	238	45	12	52	2509	9	1	93	228	19	11	197	15	0	13	150	135	2635
2019	Mumps	18916	1925	6068	3145	13250	24238	23101	6854	6017	17238	2719	16893	16343	29796	2725	16548	9910	1992	3369	299961	1239	3958	7957	7811	2073	7225	16159	1229	1013	5764	17973	6513	303105
2019	Pertussis	326	216	2100	386	450	4974	550	1004	225	963	76	533	573	4266	146	258	589	61	16	30027	73	13	2379	5063	144	482	2276	582	6	430	132	735	30727
2019	Rubella	710	333	5501	1542	3155	3096	1015	503	430	1247	407	408	591	4096	397	939	794	210	404	32539	66	43	1217	375	261	132	2429	121	12	414	961	730	34151
2019	Scarlet fever	2077	2965	840	1122	1608	3893	898	615	13	5941	2333	2411	1585	2624	2591	5685	142	2067	4710	81737	1493	572	3767	12365	3595	3246	1812	3163	79	2808	2078	2639	83028
2019	Syphilis	30422	5084	20158	27241	7177	62760	17488	17583	6809	10804	8601	18279	14658	33276	10160	28054	18693	4526	15730	535819	3527	4351	10642	20501	12216	13540	37367	2879	1750	21442	19393	30708	587402
2019	Tuberculosis	30767	6941	22398	17286	9542	66261	39962	36904	8429	29652	22474	49753	36617	56620	12635	25159	32905	10810	22994	775764	2536	8115	21467	27800	6476	12598	50337	3897	6274	42043	29842	26270	1034760
2019	Typhus	114	3	4	8	10	112	48	3	14	93	1	10	71	34	3	0	1	0	15	1173	0	0	22	113	0	13	86	10	0	0	373	12	1201
2020	AHC	3218	16	1461	229	573	2265	4598	668	677	3383	18	2627	2865	589	82	212	788	9	68	28471	122	47	503	1075	7	293	420	2	23	5	1160	468	28398
2020	AIDS	1094	406	3583	1098	541	3960	6972	4162	213	1103	551	2885	1361	2832	296	1291	1711	521	976	62167	108	147	951	1031	404	576	13850	270	47	1503	6078	1646	62196
2020	Brucellosis	231	50	52	110	2956	355	120	52	11	2968	2884	3110	74	161	15944	166	49	1136	2965	47245	2934	263	1087	2372	4	3365	129	137	46	3010	381	123	48650
2020	Dengue fever	4	0	6	20	0	58	373	0	3	1	0	5	2	2	0	10	3	0	0	778	0	0	0	5	7	0	7	2	0	0	250	20	834
2020	Dysentery	3424	2751	3041	218	2527	926	1353	653	99	3371	1024	7187	1299	1223	692	1537	1753	367	2010	57820	403	465	2143	1794	28	1536	4586	5750	690	1224	2268	1478	57626
2020	Echinococcosis	4	2	4	1	231	1	2	3	0	2	0	15	0	2	63	2	0	0	1	3327	146	1366	10	9	0	0	387	0	97	965	12	2	3625
2020	Enteric fever	143	1	70	477	31	1034	1003	382	82	224	29	162	238	768	36	76	141	1	65	7011	26	5	30	40	15	226	437	79	3	49	992	146	7288

2020	Gonorrhea	3359	1040	2809	5094	813	25619	6402	3370	1941	842	432	3048	1802	4113	1042	5874	3304	487	1012	105160	229	237	1286	3358	2489	956	2993	269	182	765	6815	13178	106695
2020	HFMD	54158	2172	28519	26864	2753	60342	29827	29601	4419	4999	127	47375	24385	114400	881	44934	35745	162	458	761355	580	762	24817	19265	5574	4551	63929	1131	1503	391	68443	58288	801813
2020	HFRS	180	3	11	347	128	180	7	24	0	262	882	212	610	659	55	183	481	255	461	8121	14	2	2051	534	2	17	64	15	2	0	210	270	8356
2020	Hepatitis A	478	65	526	452	350	1530	683	238	92	302	164	156	571	430	173	519	248	130	2214	14815	43	195	179	549	205	1177	1210	46	46	284	1157	403	15368
2020	Hepatitis B	66709	1325	8610	39014	10748	137109	52985	20054	12445	49099	8173	50565	46726	73408	14575	12714	39423	5105	15662	902476	2066	9178	13697	69954	8605	35895	42944	2696	3762	22670	13929	12631	1135302
2020	Hepatitis C	9787	511	3998	2024	6194	24004	8721	5440	1969	7698	2837	18241	7191	15266	5591	4208	2316	3046	7120	194066	720	1702	6735	5635	1712	7568	13539	1274	81	6194	9485	3259	227324
2020	Hepatitis E	980	147	760	512	148	1995	868	421	361	538	170	295	1290	865	125	1676	406	108	847	19034	15	102	240	811	510	553	1251	44	18	192	1534	1252	19629
2020	Infectious diarrhea	94813	13105	33773	20170	17155	129450	118143	18353	4777	55039	2318	60853	29042	23038	4634	17185	31221	538	10509	1062277	6825	5497	25169	121356	2728	21256	41914	24180	160	9391	37618	82067	1061632
2020	JE	27	1	4	0	12	9	7	10	0	5	0	54	0	7	0	2	2	1	0	288	2	0	18	43	1	7	29	0	0	0	43	4	351
2020	Malaria	34	9	9	42	4	151	69	4	3	28	5	71	35	47	0	89	15	2	16	1023	2	0	30	71	14	6	73	3	0	4	133	54	1143
2020	Mumps	6278	1068	3845	1865	4241	12464	10243	3435	2033	5855	1046	7697	5158	8598	1766	6925	3294	970	2260	129120	1013	1377	4660	4365	1203	2850	8311	770	576	2416	8356	4182	129336
2020	Pertussis	27	13	308	104	93	849	101	167	30	118	7	83	30	728	18	14	119	3	1	4475	2	0	415	516	8	130	344	49	0	30	112	56	5005
2020	Rubella	32	35	412	53	177	136	72	33	48	77	13	17	17	56	19	103	33	1	23	2201	0	259	11	16	15	7	97	12	7	81	267	72	2853
2020	Scarlet fever	557	300	231	383	363	1600	380	275	6	727	226	553	209	728	427	1346	73	223	605	16564	241	223	619	1997	763	524	660	274	27	363	904	757	17180
2020	Syphilis	27248	3648	17571	21635	6535	53483	14653	19501	6619	9040	5874	17503	11026	30569	7824	25309	15579	3373	13456	464435	2750	4551	9917	17691	9264	11569	34515	2650	1632	16937	18312	24201	519372
2020	Tuberculosis	26656	6150	20836	16217	8119	58065	34913	34976	7875	24997	15316	41712	31329	52539	10008	22922	26141	8546	19098	670538	2066	5705	18319	24917	5908	11091	46218	3204	5264	27728	29182	24521	869898
2020	Typhus	104	1	0	14	9	126	76	1	10	80	0	9	77	30	2	0	1	0	16	1069	0	0	14	59	0	4	83	8	0	0	334	11	1210
2021	AHC	3739		100		0	0						2735				0								889	3		418			8		264	28514
2021	AIDS	1120		496		0	4906						2933				1590								902	403		11697			1422		886	61032
2021	Brucellosis	376		5		0	504						4871				294								3276	3		212			4989		99	73645
2021	Dengue fever	0		0		0	17						1				0								1	0		1			0		0	48
2021	Dysentery	2454		133		0	820						5849				1186								1423	19		3926			807		698	50902
2021	Echinococcosis	0		1		0	0						16				0								2	1		725			893		2	3425
2021	Enteric fever	130		2		0	1147						108				107								47	11		480			63		57	7480
2021	Gonorrhea	4404		290		0	29584						3260				7584								4036	3129		3873			1088		8011	130022
2021	HFMD	50943		4357		0	0						21842				0								31400	14895		82034			1556		66177	1368244
2021	HFRS	189		0		0	189						395				212								734	2		208			5		107	9483
2021	Hepatitis A	491		60		0	1382						162				0								350	206		983			272		214	12393
2021	Hepatitis B	82651		2383		0	212818						65981				0								77641	168		66566			29856		9036	1240851
2021	Hepatitis C	11042		573		0	30157						23024				0								5662	41		18275			7541		2319	244528
2021	Hepatitis E	1457		78		0	2228						579				0								1175	908		1405			184		1532	26802
2021	Infectious diarrhea	99627		3573		0	0						63730				0								128182	4470		57207			11467		69333	1342024
2021	JE	10		0		0	5						24				2								12	0		22			0		0	225

2021	Malaria	23	0	0	161	44	37	37	60	59	1	25	850
2021	Mumps	6056	306	0	0	5527	0	3544	1408	7713	1814	1887	120776
2021	Pertussis	265	97	0	1137	136	151	353	113	1612	3	22	9162
2021	Rubella	41	4	0	0	17	0	93	5	75	27	34	1302
2021	Scarlet fever	736	49	0	3939	562	2044	2548	970	1713	501	741	29507
2021	Syphilis	27500	1589	0	62948	18920	31832	18283	11106	37957	22153	15469	537720
2021	Tuberculosis	33834	2008	0	73374	42686	27889	26961	5968	60195	27342	14953	828074
2021	Typhus	148	0	0	0	16	0	45	0	125	0	3	1336
2022	AHC	3475	1125	50	0	2590	0	0	0	403	11	87	26285
2022	AIDS	1020	3445	59	4359	2361	1319	0	282	10261	1276	353	52709
2022	Brucellosis	363	46	142	506	5386	298	0	8	279	6077	46	71437
2022	Dengue fever	0	0	0	10	1	1	0	0	2	0	0	548
2022	Dysentery	1719	1727	32	561	5018	789	0	4	2539	475	254	36306
2022	Echinococcosis	3	3	10	0	8	0	0	0	685	747	3	2745
2022	Enteric fever	141	60	0	1044	71	73	0	1	313	27	18	6029
2022	Gonorrhea	3643	2560	21	22618	2259	5436	0	1407	3333	825	3425	99094
2022	HFMD	28957	31256	28	0	26914	0	0	2622	52524	3459	16461	685249
2022	HFRS	101	5	4	135	207	140	0	1	209	1	33	5558
2022	Hepatitis A	462	615	13	1518	172	0	0	166	848	156	105	10893
2022	Hepatitis B	83791	22367	371	224333	60669	0	0	123	74046	23986	3778	1191002
2022	Hepatitis C	9967	6083	209	28762	19752	0	0	25	20313	5993	1136	219141
2022	Hepatitis E	1680	1040	3	2321	639	0	0	712	1322	127	684	26571
2022	Infectious diarrhea	83753	40219	317	0	52354	0	0	2095	57595	7099	21389	969611
2022	JE	1	3	0	4	19	0	0	0	20	0	0	173
2022	Malaria	19	12	0	178	61	37	0	23	75	0	12	857
2022	Mumps	5382	3606	50	0	5467	0	0	626	7339	1206	881	105108
2022	Pertussis	1162	2891	12	1945	2669	896	0	334	4029	121	795	39781
2022	Rubella	46	67	5	0	30	0	0	1	85	35	15	1284
2022	Scarlet fever	674	664	4	2284	306	1449	0	370	1286	367	441	21216
2022	Syphilis	27408	18034	190	64172	15940	28293	0	7913	39174	16103	7334	497934
2022	Tuberculosis	28284	20570	432	66674	36661	23961	0	4120	54354	20675	6803	712586
2022	Typhus	102	4	0	0	6	0	0	0	109	0	3	1322
2023	AHC	4039	751	852	49352	3147	92	0	9	632	18	672	198183

2023	AIDS	1326	3624	662	4623	2721	1498	0	495	11074	1729	888	59533
2023	Brucellosis	444	89	5630	650	5437	329	0	6	448	9992	96	75858
2023	Dengue fever	26	93	3	4195	25	65	0	39	192	5	354	19627
2023	Dysentery	1561	1291	1402	610	5202	707	0	12	2851	639	480	37425
2023	Echinococcosis	1	2	235	1	13	1	0	0	971	1181	0	3964
2023	Enteric fever	158	36	27	880	80	76	0	4	326	48	42	5749
2023	Gonorrhea	3968	2511	686	24096	2630	6278	0	2209	4138	1139	7921	106233
2023	HFMD	73574	61818	9152	364894	34706	59016	0	28108	99902	5719	104002	1682860
2023	HFRS	101	5	40	129	149	118	0	2	222	0	74	5549
2023	Hepatitis A	500	590	189	1732	227	0	0	201	1052	196	302	12015
2023	Hepatitis B	109167	24589	11297	243972	73769	0	0	172	122002	34159	9262	1430394
2023	Hepatitis C	10729	5966	6087	30171	24792	0	0	26	24005	9387	1905	242607
2023	Hepatitis E	1777	1083	99	2959	802	0	0	913	1975	138	1507	30575
2023	Infectious diarrhea	95842	36910	20224	112446	61882	6532	0	3746	78953	14723	50778	1168581
2023	JE	4	2	20	4	16	4	0	0	23	0	5	235
2023	Malaria	78	33	26	134	254	125	0	9	167	3	55	2406
2023	Mumps	4879	2700	1751	6625	5008	2012	0	622	6338	1900	1876	91972
2023	Pertussis	210	320	443	8131	1033	195	0	236	702	345	1135	38205
2023	Rubella	38	31	26	95	22	39	0	0	46	11	26	1002
2023	Scarlet fever	760	371	552	2334	530	1259	0	338	1190	1357	622	25276
2023	Syphilis	33127	19760	7149	78941	20411	34876	0	10189	53035	28820	14697	616933
2023	Tuberculosis	29922	20581	15953	69242	42846	25325	0	5191	57445	27580	14167	773512
2023	Typhus	184	4	4	174	9	0	0	0	194	2	6	1681

Supplementary Table 2. Adjusted Incidence and Relative Ratio (IRR) Analysis of Notifiable Infectious Diseases by Period and Province in China

disease	class	Periods	forecast	actual	diff	percent	IRR_1	IRR_2	IRR_3
AHC	Intestinal infectious diseases	Epidemic period	11496.01	4463.00	7033.01	0.61	0.38	0.39	0.39
AHC	Intestinal infectious diseases	PHSMs period I	7755.54	5822.00	1933.54	0.25	0.72	0.83	0.86
AIDS	Blood borne and sexually transmitted diseases	Epidemic period	23465.58	11378.00	12087.58	0.52	0.42	0.49	0.52
AIDS	Blood borne and sexually transmitted diseases	PHSMs period I	13919.98	9700.00	4219.98	0.30	0.65	0.72	0.74
Brucellosis	Zoonotic infectious diseases	Epidemic period	10084.45	6707.00	3377.45	0.33	0.61	0.68	0.74
Brucellosis	Zoonotic infectious diseases	PHSMs period I	9809.77	6886.00	2923.77	0.30	0.56	0.76	0.86
Dengue fever	Zoonotic infectious diseases	Epidemic period	8029.85	186.00	7843.85	0.98	0.01	0.01	0.02
Dengue fever	Zoonotic infectious diseases	PHSMs period I	348.92	104.00	244.92	0.70	0.23	0.28	0.32
Dysentery	Intestinal infectious diseases	Epidemic period	9327.29	5114.00	4213.29	0.45	0.47	0.54	0.64
Dysentery	Intestinal infectious diseases	PHSMs period I	10340.08	8113.00	2227.08	0.22	0.67	0.74	0.87
Echinococcosis	Zoonotic infectious diseases	Epidemic period	996.94	481.00	515.94	0.52	0.34	0.39	0.62
Echinococcosis	Zoonotic infectious diseases	PHSMs period I	1084.84	626.00	458.84	0.42	0.46	0.55	0.65
Enteric fever	Intestinal infectious diseases	Epidemic period	1959.50	837.00	1122.50	0.57	0.32	0.40	0.54
Enteric fever	Intestinal infectious diseases	PHSMs period I	1954.37	1237.00	717.37	0.37	0.59	0.60	0.65
Gonorrhea	Blood borne and sexually transmitted diseases	Epidemic period	30255.94	18419.00	11836.94	0.39	0.54	0.57	0.65
Gonorrhea	Blood borne and sexually transmitted diseases	PHSMs period I	26041.91	16439.00	9602.91	0.37	0.49	0.50	0.69
Hepatitis A	Intestinal infectious diseases	Epidemic period	3669.29	1804.00	1865.29	0.51	0.44	0.47	0.53
Hepatitis A	Intestinal infectious diseases	PHSMs period I	3552.76	4075.00	-522.24	-0.15	1.12	1.13	1.17
Hepatitis B	Blood borne and sexually transmitted diseases	Epidemic period	331032.50	220659.00	110373.50	0.33	0.61	0.67	0.73
Hepatitis B	Blood borne and sexually transmitted diseases	PHSMs period I	318548.73	230682.00	87866.73	0.28	0.64	0.74	0.81
Hepatitis C	Blood borne and sexually transmitted diseases	Epidemic period	70293.20	38892.00	31401.20	0.45	0.51	0.54	0.59
Hepatitis C	Blood borne and sexually transmitted diseases	PHSMs period I	66286.77	43073.00	23213.77	0.35	0.56	0.64	0.73
Hepatitis E	Intestinal infectious diseases	Epidemic period	6463.36	4063.00	2400.36	0.37	0.53	0.57	0.72
Hepatitis E	Intestinal infectious diseases	PHSMs period I	7913.85	4312.00	3601.85	0.46	0.48	0.51	0.60
HFMD	Intestinal infectious diseases	Epidemic period	350520.93	80864.00	269656.93	0.77	0.13	0.23	0.29
HFMD	Intestinal infectious diseases	PHSMs period I	115941.54	34487.00	81454.54	0.70	0.09	0.12	0.40
HFRS	Zoonotic infectious diseases	Epidemic period	3359.17	1624.00	1735.17	0.52	0.31	0.43	0.65
HFRS	Zoonotic infectious diseases	PHSMs period I	2691.04	1491.00	1200.04	0.45	0.53	0.58	0.59
Infectious diarrhea	Intestinal infectious diseases	Epidemic period	415577.87	122932.00	292645.87	0.70	0.24	0.27	0.36
Infectious diarrhea	Intestinal infectious diseases	PHSMs period I	374263.96	215474.00	158789.96	0.42	0.40	0.43	0.65
JE	Zoonotic infectious diseases	Epidemic period	28.81	8.00	20.81	0.72	0.18	0.28	0.50

JE	Zoonotic infectious diseases	PHSMs period I	10.62	6.00	4.62	0.44	0.26	0.46	0.86
Malaria	Zoonotic infectious diseases	Epidemic period	647.83	302.00	345.83	0.53	0.38	0.41	0.51
Malaria	Zoonotic infectious diseases	PHSMs period I	716.37	566.00	150.37	0.21	0.50	0.51	0.88
Mumps	Respiratory infectious diseases	Epidemic period	78928.73	14911.00	64017.73	0.81	0.12	0.14	0.24
Mumps	Respiratory infectious diseases	PHSMs period I	56784.01	31205.00	25579.01	0.45	0.45	0.58	0.64
Pertussis	Respiratory infectious diseases	Epidemic period	10644.34	4336.00	6308.34	0.59	0.31	0.36	0.47
Pertussis	Respiratory infectious diseases	PHSMs period I	5497.78	2753.00	2744.78	0.50	0.40	0.47	0.66
Rubella	Respiratory infectious diseases	Epidemic period	5195.53	232.00	4963.53	0.96	0.03	0.03	0.06
Rubella	Respiratory infectious diseases	PHSMs period I	5168.49	1702.00	3466.49	0.67	0.20	0.33	0.73
Scarlet fever	Respiratory infectious diseases	Epidemic period	45301.38	3198.00	42103.38	0.93	0.04	0.06	0.09
Scarlet fever	Respiratory infectious diseases	PHSMs period I	18836.86	7376.00	11460.86	0.61	0.11	0.16	0.45
Syphilis	Blood borne and sexually transmitted diseases	Epidemic period	178980.23	88227.00	90753.23	0.51	0.45	0.50	0.54
Syphilis	Blood borne and sexually transmitted diseases	PHSMs period I	142285.20	102273.00	40012.20	0.28	0.64	0.75	0.80
Tuberculosis	Respiratory infectious diseases	Epidemic period	191823.27	136033.00	55790.27	0.29	0.64	0.74	0.79
Tuberculosis	Respiratory infectious diseases	PHSMs period I	246021.74	186042.00	59979.74	0.24	0.69	0.74	0.81
Typhus	Zoonotic infectious diseases	Epidemic period	249.23	185.00	64.23	0.26	0.46	0.49	0.83
Typhus	Zoonotic infectious diseases	PHSMs period I	174.61	129.00	45.61	0.26	0.67	0.85	0.86
AHC	Intestinal infectious diseases	PHSMs period II	157661.26	74068.00	83593.26	0.53	0.39	0.50	0.64
AIDS	Blood borne and sexually transmitted diseases	PHSMs period II	213583.85	156674.00	56909.85	0.27	0.66	0.74	0.82
Brucellosis	Zoonotic infectious diseases	PHSMs period II	150252.27	182457.00	-32204.73	-0.21	1.06	1.22	1.42
Dengue fever	Zoonotic infectious diseases	PHSMs period II	119076.49	1141.00	117935.49	0.99	0.00	0.01	0.02
Dysentery	Intestinal infectious diseases	PHSMs period II	177671.40	133531.00	44140.40	0.25	0.69	0.78	0.85
Echinococcosis	Zoonotic infectious diseases	PHSMs period II	9305.34	8928.00	377.34	0.04	0.89	1.00	1.04
Enteric fever	Intestinal infectious diseases	PHSMs period II	25465.10	18907.00	6558.10	0.26	0.66	0.77	0.84
Gonorrhea	Blood borne and sexually transmitted diseases	PHSMs period II	310608.27	305715.00	4893.27	0.02	0.88	1.03	1.08
HFMD	Intestinal infectious diseases	PHSMs period II	5313864.93	2742439.00	2571425.93	0.48	0.29	0.55	0.81
HFRS	Zoonotic infectious diseases	PHSMs period II	24766.54	20499.00	4267.54	0.17	0.49	0.71	0.86
Hepatitis A	Intestinal infectious diseases	PHSMs period II	39669.36	33298.00	6371.36	0.16	0.78	0.84	0.92
Hepatitis B	Blood borne and sexually transmitted diseases	PHSMs period II	3387283.17	3190604.00	196679.16	0.06	0.92	0.95	0.97
Hepatitis C	Blood borne and sexually transmitted diseases	PHSMs period II	710513.99	621813.00	88700.99	0.12	0.84	0.88	0.92
Hepatitis E	Intestinal infectious diseases	PHSMs period II	72685.59	65771.00	6914.59	0.10	0.82	0.94	1.00
Infectious diarrhea	Intestinal infectious diseases	PHSMs period II	3753301.84	3077811.00	675490.84	0.18	0.70	0.80	0.92
JE	Zoonotic infectious diseases	PHSMs period II	1782.26	739.00	1043.26	0.59	0.21	0.44	0.67

Malaria	Zoonotic infectious diseases	PHSMs period II	6955.19	2131.00	4824.19	0.69	0.23	0.28	0.36
Mumps	Respiratory infectious diseases	PHSMs period II	811380.44	311474.00	499906.44	0.62	0.30	0.38	0.49
Pertussis	Respiratory infectious diseases	PHSMs period II	126168.65	47742.00	78426.65	0.62	0.10	0.18	0.60
Rubella	Respiratory infectious diseases	PHSMs period II	113754.01	3545.00	110209.01	0.97	0.02	0.04	0.08
Scarlet fever	Respiratory infectious diseases	PHSMs period II	289024.66	57605.00	231419.66	0.80	0.17	0.22	0.27
Syphilis	Blood borne and sexually transmitted diseases	PHSMs period II	1780198.88	1393234.00	386964.88	0.22	0.74	0.79	0.84
Tuberculosis	Respiratory infectious diseases	PHSMs period II	2428759.56	2142213.00	286546.56	0.12	0.86	0.89	0.92
Typhus	Zoonotic infectious diseases	PHSMs period II	2818.14	3587.00	-768.86	-0.27	0.92	1.21	1.50
AHC	Intestinal infectious diseases	Post-epidemic period	79925.29	197027.00	-117101.71	-1.47	0.50	0.83	1.28
AIDS	Blood borne and sexually transmitted diseases	Post-epidemic period	92372.97	57718.00	34654.97	0.38	0.59	0.64	0.66
Brucellosis	Zoonotic infectious diseases	Post-epidemic period	55344.53	73540.00	-18195.47	-0.33	1.19	1.33	1.41
Dengue fever	Zoonotic infectious diseases	Post-epidemic period	108071.03	19626.00	88445.03	0.82	0.03	0.10	0.16
Dysentery	Intestinal infectious diseases	Post-epidemic period	51440.35	35501.00	15939.35	0.31	0.64	0.67	0.75
Echinococcosis	Zoonotic infectious diseases	Post-epidemic period	2613.30	3724.00	-1110.70	-0.43	1.26	1.53	1.61
Enteric fever	Intestinal infectious diseases	Post-epidemic period	8542.12	5565.00	2977.12	0.35	0.62	0.69	0.71
Gonorrhea	Blood borne and sexually transmitted diseases	Post-epidemic period	108252.17	101471.00	6781.17	0.06	0.90	0.92	0.97
HFMD	Intestinal infectious diseases	Post-epidemic period	2181032.61	1680376.00	500656.61	0.23	0.22	0.58	0.90
HFRS	Zoonotic infectious diseases	Post-epidemic period	8467.20	5332.00	3135.20	0.37	0.40	0.45	0.70
Hepatitis A	Intestinal infectious diseases	Post-epidemic period	13967.51	11492.00	2475.51	0.18	0.78	0.80	0.85
Hepatitis B	Blood borne and sexually transmitted diseases	Post-epidemic period	1265296.96	1355604.00	-90307.04	-0.07	0.99	1.08	1.14
Hepatitis C	Blood borne and sexually transmitted diseases	Post-epidemic period	268287.00	229822.00	38465.00	0.14	0.82	0.84	0.88
Hepatitis E	Intestinal infectious diseases	Post-epidemic period	25722.48	29431.00	-3708.52	-0.14	1.04	1.17	1.28
Infectious diarrhea	Intestinal infectious diseases	Post-epidemic period	1437216.50	1125631.00	311585.50	0.22	0.73	0.81	0.89
JE	Zoonotic infectious diseases	Post-epidemic period	483.82	231.00	252.82	0.52	0.39	0.44	0.70
Malaria	Zoonotic infectious diseases	Post-epidemic period	2306.14	2257.00	49.14	0.02	0.92	0.97	1.10
Mumps	Respiratory infectious diseases	Post-epidemic period	289867.51	89602.00	200265.51	0.69	0.26	0.29	0.36
Pertussis	Respiratory infectious diseases	Post-epidemic period	78350.40	37322.00	41028.40	0.52	0.18	0.28	0.65
Rubella	Respiratory infectious diseases	Post-epidemic period	49295.44	962.00	48333.44	0.98	0.02	0.03	0.06
Scarlet fever	Respiratory infectious diseases	Post-epidemic period	128228.66	25000.00	103228.66	0.81	0.10	0.22	0.28
Syphilis	Blood borne and sexually transmitted diseases	Post-epidemic period	707691.49	588225.00	119466.49	0.17	0.80	0.82	0.87
Tuberculosis	Respiratory infectious diseases	Post-epidemic period	767909.62	719782.00	48127.62	0.06	0.89	0.90	0.95
Typhus	Zoonotic infectious diseases	Post-epidemic period	928.38	1648.00	-719.62	-0.78	1.40	1.82	2.09

Supplementary Table 3. Normalized Performance Evaluation of Forecasting Models for Notifiable Infectious Diseases

disease	Train length	Test length	Index Range	Local Best	Index Select	Method Select
AHC	84	24	-2.07, -1.81, 0.23, 0.52, 1.19, 1.94	Hybrid*	1.94	Hybrid*
AHC	90	24	-2.01, -1.82, -0.01, 0.62, 1.59, 1.63	ETS	1.59	Hybrid*
AHC	96	24	-4.79, -1.31, -0.36, 1.63, 2.28, 2.55	Neural Network	2.28	Hybrid*
AHC	102	24	-1.48, -1.05, 0.46, 0.51, 0.69, 0.87	Neural Network	0.69	Hybrid*
AHC	108	24	-3.00, -2.68, -0.06, 0.72, 2.08, 2.94	Hybrid*	2.94	Hybrid*
AHC	114	24	-1.90, -1.10, -0.17, 0.89, 0.99, 1.28	ETS	0.89	Hybrid*
AHC	120	24	-2.36, -1.42, -0.32, 0.74, 1.13, 2.22	Hybrid*	2.22	Hybrid*
AIDS	84	24	-4.35, -1.05, -0.01, 0.11, 0.94, 4.35	ETS	-1.05	SARIMA
AIDS	90	24	-2.89, -2.61, -0.94, 1.97, 2.08, 2.39	Prophet	2.08	SARIMA
AIDS	96	24	-4.52, -1.06, -0.27, 0.75, 1.02, 4.08	SARIMA	4.08	SARIMA
AIDS	102	24	-5.13, -0.78, 0.57, 0.59, 1.75, 3.00	Bayesian Structural	1.75	SARIMA
AIDS	108	24	-5.42, -1.20, 0.37, 1.72, 2.24, 2.30	ETS	2.24	SARIMA
AIDS	114	24	-3.20, -2.48, -0.74, 0.39, 2.73, 3.30	Bayesian Structural	2.73	SARIMA
AIDS	120	24	-4.37, -1.13, -0.97, -0.07, 2.46, 4.09	SARIMA	4.09	SARIMA
Brucellosis	84	24	-3.49, -3.01, 0.22, 0.51, 2.04, 3.73	Bayesian Structural	0.22	Hybrid*
Brucellosis	90	24	-0.98, -0.73, -0.62, 0.00, 1.10, 1.22	Prophet	-0.73	Hybrid*
Brucellosis	96	24	-2.87, -2.10, -1.00, -0.12, 0.84, 5.26	Bayesian Structural	-0.12	Hybrid*
Brucellosis	102	24	-1.40, -0.72, -0.58, 0.16, 0.35, 2.19	Neural Network	0.16	Hybrid*
Brucellosis	108	24	-3.80, -2.81, -0.42, 0.60, 2.74, 3.69	Neural Network	0.6	Hybrid*
Brucellosis	114	24	-1.84, -1.21, 0.17, 0.45, 0.82, 1.61	Neural Network	0.45	Hybrid*
Brucellosis	120	24	-3.80, -1.84, -0.62, 1.27, 1.69, 3.30	Hybrid*	3.3	Hybrid*
Dengue fever	84	24	-3.16, -1.84, 0.00, 1.12, 1.68, 2.19	ETS	1.68	SARIMA
Dengue fever	90	24	-1.59, -1.19, -0.41, 0.53, 0.73, 1.93	Prophet	0.73	SARIMA
Dengue fever	96	24	-2.28, -2.17, -1.60, 0.35, 2.18, 3.52	SARIMA	3.52	SARIMA
Dengue fever	102	24	-2.17, -1.46, -0.47, 1.10, 1.38, 1.62	Prophet	-2.17	SARIMA
Dengue fever	108	24	-3.43, -2.28, 0.65, 0.74, 1.75, 2.58	Hybrid*	0.74	SARIMA
Dengue fever	114	24	-3.40, -0.61, -0.12, 0.63, 0.75, 2.75	Prophet	-0.12	SARIMA
Dengue fever	120	24	-2.62, -1.71, -0.30, -0.24, 1.04, 3.82	SARIMA	3.82	SARIMA
Dysentery	84	24	-5.86, 0.18, 1.21, 1.45, 1.48, 1.54	SARIMA	1.48	ETS
Dysentery	90	24	-5.83, 0.27, 1.33, 1.39, 1.39, 1.45	Neural Network	1.33	ETS
Dysentery	96	24	-5.57, -0.41, 1.31, 1.44, 1.53, 1.70	ETS	1.7	ETS

Dysentery	102	24	-5.81, 0.21, 1.40, 1.40, 1.40, 1.40	SARIMA	1.4	ETS
Dysentery	108	24	-5.55, -0.64, 1.51, 1.54, 1.56, 1.59	ETS	1.59	ETS
Dysentery	114	24	-5.77, 0.19, 1.37, 1.39, 1.40, 1.42	Neural Network	1.4	ETS
Dysentery	120	24	-5.46, -0.90, 1.42, 1.55, 1.66, 1.73	ETS	1.73	ETS
Echinococcosis	84	24	-3.88, -0.96, 1.00, 1.03, 1.10, 1.72	Prophet	-3.88	Bayesian Structural
Echinococcosis	90	24	-2.19, -1.41, 0.37, 0.41, 0.75, 2.07	SARIMA	-2.19	Bayesian Structural
Echinococcosis	96	24	-3.79, -0.76, -0.49, 0.75, 1.08, 3.21	Prophet	-3.79	Bayesian Structural
Echinococcosis	102	24	-2.37, -0.40, 0.05, 0.07, 1.10, 1.55	Bayesian Structural	1.55	Bayesian Structural
Echinococcosis	108	24	-2.64, -1.75, -0.82, 1.58, 1.65, 1.98	SARIMA	-1.75	Bayesian Structural
Echinococcosis	114	24	-2.95, -2.02, 0.15, 1.23, 1.78, 1.80	Prophet	-2.02	Bayesian Structural
Echinococcosis	120	24	-3.93, -2.00, 0.36, 1.37, 2.03, 2.17	Bayesian Structural	2.17	Bayesian Structural
Enteric fever	84	24	-2.70, -2.27, -0.53, -0.14, 0.46, 5.18	Bayesian Structural	0.46	Neural Network
Enteric fever	90	24	-2.41, -1.80, 0.20, 0.94, 1.25, 1.82	SARIMA	-1.8	Neural Network
Enteric fever	96	24	-5.09, -0.73, 0.40, 1.04, 1.88, 2.50	Neural Network	2.5	Neural Network
Enteric fever	102	24	-3.28, 0.07, 0.18, 0.24, 0.63, 2.17	SARIMA	0.18	Neural Network
Enteric fever	108	24	-1.20, -1.10, -0.69, -0.33, 1.25, 2.08	SARIMA	-0.33	Neural Network
Enteric fever	114	24	-1.47, -1.06, -0.69, 0.76, 0.83, 1.63	ETS	-1.06	Neural Network
Enteric fever	120	24	-2.80, -0.89, -0.64, 0.71, 1.53, 2.09	Neural Network	2.09	Neural Network
Gonorrhea	84	24	-4.88, -0.71, -0.38, 0.75, 1.14, 4.08	SARIMA	4.08	SARIMA
Gonorrhea	90	24	-3.47, -1.71, 0.11, 0.12, 0.36, 4.59	SARIMA	4.59	SARIMA
Gonorrhea	96	24	-4.87, -0.59, -0.44, -0.38, 2.75, 3.54	SARIMA	3.54	SARIMA
Gonorrhea	102	24	-5.15, -1.88, 0.61, 1.77, 2.15, 2.50	SARIMA	2.5	SARIMA
Gonorrhea	108	24	-4.68, -1.22, -0.89, 1.79, 2.18, 2.82	Prophet	1.79	SARIMA
Gonorrhea	114	24	-2.06, -1.87, 0.16, 1.05, 1.20, 1.53	Prophet	1.05	SARIMA
Gonorrhea	120	24	-2.20, -1.62, -1.40, -0.50, 2.77, 2.95	SARIMA	2.95	SARIMA
HFMD	84	24	-4.54, -1.58, -1.36, 2.38, 2.40, 2.70	Hybrid*	2.7	Hybrid*
HFMD	90	24	-1.52, -0.70, 0.25, 0.36, 0.71, 0.90	Neural Network	0.36	Hybrid*
HFMD	96	24	-3.60, -1.90, -1.77, 2.01, 2.40, 2.86	Hybrid*	2.86	Hybrid*
HFMD	102	24	-2.03, -0.71, -0.08, 0.25, 1.08, 1.50	Prophet	0.25	Hybrid*
HFMD	108	24	-4.23, -1.09, -0.90, 1.78, 1.80, 2.64	SARIMA	1.8	Hybrid*
HFMD	114	24	-3.43, -0.79, 0.21, 0.94, 1.36, 1.71	Neural Network	0.94	Hybrid*
HFMD	120	24	-5.52, -0.26, 0.11, 0.63, 2.24, 2.81	Hybrid*	2.81	Hybrid*
HFRS	84	24	-5.36, -0.40, -0.10, 1.06, 2.38, 2.42	SARIMA	-0.1	Neural Network

HFRS	90	24	-2.04, -1.14, -0.72, 0.08, 1.67, 2.15	Neural Network	2.15	Neural Network
HFRS	96	24	-5.93, 0.17, 0.71, 1.34, 1.81, 1.90	SARIMA	0.71	Neural Network
HFRS	102	24	-1.96, -1.06, -0.89, 0.80, 1.24, 1.86	SARIMA	-0.89	Neural Network
HFRS	108	24	-5.66, -0.11, 0.92, 1.14, 1.60, 2.11	ETS	-0.11	Neural Network
HFRS	114	24	-2.87, -0.79, -0.12, 0.75, 1.46, 1.58	Bayesian Structural	-0.12	Neural Network
HFRS	120	24	-4.53, -2.64, 1.04, 1.27, 2.39, 2.48	Neural Network	2.48	Neural Network
Hepatitis A	84	24	-2.41, -1.30, -0.68, 0.30, 1.16, 2.94	Prophet	-1.3	ETS
Hepatitis A	90	24	-3.11, -1.09, -0.78, 1.33, 1.62, 2.03	SARIMA	1.62	ETS
Hepatitis A	96	24	-2.27, -1.60, -1.15, -0.13, 1.52, 3.63	SARIMA	-0.13	ETS
Hepatitis A	102	24	-1.98, -1.94, -0.82, 1.08, 1.33, 2.33	SARIMA	1.08	ETS
Hepatitis A	108	24	-3.09, -2.25, 0.31, 0.92, 1.51, 2.59	SARIMA	0.31	ETS
Hepatitis A	114	24	-3.08, -0.46, -0.32, 0.90, 1.23, 1.73	SARIMA	0.9	ETS
Hepatitis A	120	24	-2.18, -2.09, -0.34, 1.04, 1.70, 1.87	ETS	1.87	ETS
Hepatitis B	84	24	-2.93, -1.86, 0.01, 1.25, 1.73, 1.80	ETS	-2.93	Bayesian Structural
Hepatitis B	90	24	-3.59, -1.09, 0.66, 1.19, 1.29, 1.53	SARIMA	-1.09	Bayesian Structural
Hepatitis B	96	24	-3.70, -0.73, 0.64, 0.86, 1.32, 1.61	Neural Network	0.86	Bayesian Structural
Hepatitis B	102	24	-3.92, -0.05, 0.44, 0.85, 1.10, 1.58	Bayesian Structural	1.58	Bayesian Structural
Hepatitis B	108	24	-3.98, 0.26, 0.38, 0.98, 1.16, 1.19	ETS	1.16	Bayesian Structural
Hepatitis B	114	24	-2.73, -0.26, -0.05, 0.38, 0.44, 2.22	SARIMA	0.44	Bayesian Structural
Hepatitis B	120	24	-3.54, -0.74, 0.09, 0.75, 1.27, 2.17	Bayesian Structural	2.17	Bayesian Structural
Hepatitis C	84	24	-5.76, 0.26, 0.69, 0.71, 1.34, 2.76	ETS	1.34	SARIMA
Hepatitis C	90	24	-5.63, -0.28, 0.86, 1.13, 1.56, 2.35	Prophet	1.13	SARIMA
Hepatitis C	96	24	-5.82, -0.12, 0.65, 1.12, 1.55, 2.62	SARIMA	2.62	SARIMA
Hepatitis C	102	24	-3.06, -2.10, -0.56, 1.41, 1.92, 2.39	Neural Network	-0.56	SARIMA
Hepatitis C	108	24	-3.78, -1.71, -1.23, 0.12, 1.92, 4.68	SARIMA	4.68	SARIMA
Hepatitis C	114	24	-3.27, -0.07, 0.41, 0.70, 0.91, 1.32	SARIMA	1.32	SARIMA
Hepatitis C	120	24	-3.85, -1.97, -1.09, 1.47, 1.64, 3.80	SARIMA	3.8	SARIMA
Hepatitis E	84	24	-4.63, -0.83, -0.24, 1.30, 2.15, 2.24	Hybrid*	2.24	Hybrid*
Hepatitis E	90	24	-1.81, -0.98, 0.12, 0.13, 0.67, 1.88	Neural Network	0.13	Hybrid*
Hepatitis E	96	24	-4.96, -1.32, 0.48, 1.45, 1.68, 2.67	Hybrid*	2.67	Hybrid*
Hepatitis E	102	24	-3.47, -1.17, -0.56, 1.42, 1.85, 1.94	Hybrid*	1.94	Hybrid*
Hepatitis E	108	24	-3.29, -0.88, -0.32, 0.56, 1.32, 2.59	SARIMA	1.32	Hybrid*
Hepatitis E	114	24	-2.08, -2.01, 0.15, 0.66, 1.35, 1.94	Hybrid*	1.94	Hybrid*

Hepatitis E	120	24	-5.52, -0.45, 0.61, 1.09, 1.58, 2.70	Hybrid*	2.7	Hybrid*
Infectious diarrhea	84	24	-3.03, -2.40, 0.01, 0.06, 1.16, 4.20	Bayesian Structural	1.16	SARIMA
Infectious diarrhea	90	24	-4.57, -2.34, 1.09, 1.82, 1.93, 2.05	Neural Network	1.09	SARIMA
Infectious diarrhea	96	24	-3.66, -3.33, 0.35, 1.24, 2.24, 3.17	Neural Network	1.24	SARIMA
Infectious diarrhea	102	24	-3.05, -1.45, -0.53, 0.24, 2.07, 2.72	SARIMA	2.72	SARIMA
Infectious diarrhea	108	24	-3.51, -3.35, -0.53, 1.76, 2.35, 3.28	SARIMA	3.28	SARIMA
Infectious diarrhea	114	24	-3.14, -2.49, -1.46, 0.18, 2.73, 4.19	SARIMA	4.19	SARIMA
Infectious diarrhea	120	24	-5.11, -1.83, 0.95, 0.99, 1.80, 3.20	SARIMA	3.2	SARIMA
JE	84	24	-2.70, -0.70, -0.28, -0.16, 1.37, 2.46	Hybrid*	2.46	Hybrid*
JE	90	24	-2.01, -1.63, 0.34, 0.63, 1.18, 1.48	Hybrid*	1.48	Hybrid*
JE	96	24	-3.53, -1.38, 0.43, 0.86, 1.57, 2.05	Hybrid*	2.05	Hybrid*
JE	102	24	-2.45, -1.29, 0.33, 0.83, 1.14, 1.44	Hybrid*	1.44	Hybrid*
JE	108	24	-2.71, -1.83, -0.32, 0.30, 2.18, 2.39	Hybrid*	2.39	Hybrid*
JE	114	24	-3.19, -1.41, 0.33, 1.19, 1.44, 1.64	SARIMA	1.44	Hybrid*
JE	120	24	-4.06, -1.30, -0.36, 1.72, 1.87, 2.13	Hybrid*	2.13	Hybrid*
Malaria	84	24	-5.15, -1.22, 1.29, 1.60, 1.69, 1.79	SARIMA	1.69	Hybrid*
Malaria	90	24	-5.56, -0.50, 1.26, 1.35, 1.68, 1.77	SARIMA	1.68	Hybrid*
Malaria	96	24	-5.25, -0.71, 1.14, 1.33, 1.65, 1.84	SARIMA	1.65	Hybrid*
Malaria	102	24	-5.65, -0.36, 1.19, 1.55, 1.56, 1.71	Hybrid*	1.71	Hybrid*
Malaria	108	24	-5.37, -0.82, 1.38, 1.47, 1.63, 1.71	SARIMA	1.63	Hybrid*
Malaria	114	24	-5.66, -0.49, 1.37, 1.41, 1.63, 1.74	SARIMA	1.63	Hybrid*
Malaria	120	24	-5.51, -0.50, 1.09, 1.44, 1.73, 1.75	Hybrid*	1.75	Hybrid*
Mumps	84	24	-2.16, -1.22, -0.27, 0.32, 1.64, 1.70	Hybrid*	1.7	Hybrid*
Mumps	90	24	-2.86, -1.28, -1.03, 1.56, 1.65, 1.97	ETS	1.65	Hybrid*
Mumps	96	24	-2.72, -1.26, -1.12, 1.26, 1.51, 2.33	ETS	1.51	Hybrid*
Mumps	102	24	-2.54, -0.98, -0.48, 0.87, 1.35, 1.80	ETS	1.35	Hybrid*
Mumps	108	24	-3.25, -1.88, -0.63, 1.61, 1.95, 2.20	SARIMA	1.61	Hybrid*
Mumps	114	24	-3.38, -1.28, -1.15, 1.87, 1.91, 2.03	SARIMA	1.91	Hybrid*
Mumps	120	24	-3.27, -1.85, -1.51, 1.98, 2.27, 2.37	Hybrid*	2.37	Hybrid*
Pertussis	84	24	-4.28, -2.03, -0.20, 1.21, 1.22, 4.08	SARIMA	4.08	SARIMA
Pertussis	90	24	-2.10, -1.35, -1.34, -0.20, 1.39, 3.60	Bayesian Structural	1.39	SARIMA
Pertussis	96	24	-3.80, -0.95, -0.55, 0.47, 0.77, 4.06	SARIMA	4.06	SARIMA
Pertussis	102	24	-1.37, -1.06, -0.79, 0.06, 0.46, 2.69	Prophet	0.06	SARIMA

Pertussis	108	24	-5.01, -0.53, 0.21, 0.30, 1.80, 3.23	SARIMA	3.23	SARIMA
Pertussis	114	24	-4.11, -1.33, -0.45, -0.14, 1.95, 4.08	Bayesian Structural	1.95	SARIMA
Pertussis	120	24	-4.05, -1.62, 1.00, 1.24, 1.26, 2.17	SARIMA	2.17	SARIMA
Rubella	84	24	-2.34, -1.04, -0.54, 1.02, 1.35, 1.55	ETS	1.55	ETS
Rubella	90	24	-4.35, -0.28, 1.04, 1.08, 1.17, 1.33	ETS	1.33	ETS
Rubella	96	24	-1.57, -1.21, -0.17, 0.32, 1.06, 1.57	ETS	1.57	ETS
Rubella	102	24	-3.02, -2.69, 0.39, 0.54, 1.71, 3.07	Hybrid*	0.54	ETS
Rubella	108	24	-4.64, -1.43, 0.77, 1.13, 1.56, 2.61	Neural Network	1.13	ETS
Rubella	114	24	-1.48, -1.22, 0.32, 0.37, 0.79, 1.22	ETS	1.22	ETS
Rubella	120	24	-2.37, -1.79, -1.13, 1.63, 1.73, 1.92	ETS	1.92	ETS
Scarlet fever	84	24	-4.06, -2.69, 0.37, 1.03, 1.28, 4.07	ETS	-2.69	SARIMA
Scarlet fever	90	24	-5.80, 0.09, 0.58, 0.94, 2.00, 2.20	ETS	0.58	SARIMA
Scarlet fever	96	24	-5.45, -0.62, 0.30, 0.92, 1.74, 3.11	ETS	1.74	SARIMA
Scarlet fever	102	24	-4.13, -1.45, -0.11, -0.08, 1.98, 3.80	SARIMA	3.8	SARIMA
Scarlet fever	108	24	-5.16, -0.52, -0.41, 0.60, 2.55, 2.94	SARIMA	2.94	SARIMA
Scarlet fever	114	24	-3.96, -3.22, 0.02, 1.98, 2.56, 2.63	SARIMA	2.63	SARIMA
Scarlet fever	120	24	-4.24, -2.97, 0.05, 2.22, 2.24, 2.70	SARIMA	2.7	SARIMA
Syphilis	84	24	-3.24, -1.51, -0.36, 1.21, 1.84, 2.05	ETS	-0.36	Hybrid*
Syphilis	90	24	-2.37, -1.93, -0.33, -0.08, 0.19, 4.53	SARIMA	-1.93	Hybrid*
Syphilis	96	24	-2.92, -2.31, -2.17, 0.29, 3.12, 3.99	Bayesian Structural	0.29	Hybrid*
Syphilis	102	24	-3.50, -3.06, -0.06, 0.41, 2.78, 3.44	SARIMA	-0.06	Hybrid*
Syphilis	108	24	-3.80, -1.01, -0.90, -0.23, 1.02, 4.92	SARIMA	-0.23	Hybrid*
Syphilis	114	24	-2.39, -2.35, -2.29, 0.08, 3.47, 3.48	SARIMA	0.08	Hybrid*
Syphilis	120	24	-4.02, -3.64, 1.60, 1.87, 2.10, 2.10	Hybrid*	2.1	Hybrid*
Tuberculosis	84	24	-5.14, -1.42, 1.11, 1.44, 1.77, 2.25	ETS	1.11	Bayesian Structural
Tuberculosis	90	24	-1.61, -1.24, -0.60, 0.87, 0.87, 1.71	Neural Network	-1.24	Bayesian Structural
Tuberculosis	96	24	-5.93, 0.50, 0.56, 1.20, 1.80, 1.87	SARIMA	1.2	Bayesian Structural
Tuberculosis	102	24	-2.09, -2.04, -0.72, 0.89, 1.88, 2.08	SARIMA	-2.04	Bayesian Structural
Tuberculosis	108	24	-4.87, -1.10, 0.88, 1.31, 1.64, 2.13	ETS	-1.1	Bayesian Structural
Tuberculosis	114	24	-1.95, -1.31, 0.06, 0.30, 1.37, 1.53	Neural Network	-1.31	Bayesian Structural
Tuberculosis	120	24	-5.32, -0.96, 0.53, 1.48, 2.06, 2.21	Bayesian Structural	2.21	Bayesian Structural
Typhus	84	24	-4.54, -1.74, 0.98, 1.07, 1.17, 3.05	SARIMA	3.05	SARIMA
Typhus	90	24	-2.63, -1.45, -0.26, 1.14, 1.18, 2.03	SARIMA	2.03	SARIMA

Typhus	96	24	-2.50, -2.47, -0.46, 0.86, 2.12, 2.45	Hybrid*	2.12	SARIMA
Typhus	102	24	-2.18, -2.10, -0.21, 1.48, 1.49, 1.51	SARIMA	1.51	SARIMA
Typhus	108	24	-4.24, -1.81, 0.78, 1.61, 1.78, 1.88	Hybrid*	0.78	SARIMA
Typhus	114	24	-1.60, -1.48, -0.04, 0.67, 1.07, 1.37	ETS	1.07	SARIMA
Typhus	120	24	-3.40, -1.43, 0.18, 0.91, 1.76, 1.97	SARIMA	1.97	SARIMA
