# Supplementary material for: Temporal shifts in 24 notifiable infectious diseases in China before and during the COVID-19 pandemic
Source: Nat Commun. 2024 May 8;15:3891. doi: 10.1038/s41467-024-48201-8 (PMC11079007; doi:10.1038/s41467-024-48201-8)
Supplement: Supplementary file 3 — Description of Additional Supplementary Files [file 41467_2024_48201_MOESM3_ESM.pdf]

Description of Additional Supplementary Files

File Name: Supplementary Data 1
Description: Monthly incidence of notifiable infectious diseases across national and provincial levels in China
