# Supplementary material for: Temporal shifts in 24 notifiable infectious diseases in China before and during the COVID-19 pandemic
Source: Nat Commun. 2024 May 8;15:3891. doi: 10.1038/s41467-024-48201-8 (PMC11079007; doi:10.1038/s41467-024-48201-8)
Supplement: Supplementary file 5 — Reporting Summary [file 41467_2024_48201_MOESM5_ESM.pdf]

nature portfolio

Corresponding author(s): Tianmu Chen

Last updated by author(s): Apr 23, 2024

Reporting Summary

Nature Portfolio wishes to improve the reproducibility of the work that we publish. This form provides structure for consistency and transparency in reporting. For further information on Nature Portfolio policies, see our Editorial Policies and the Editorial Policy Checklist.

Statistics

For all statistical analyses, confirm that the following items are present in the figure legend, table legend, main text, or Methods section.

n/a	Confirmed	
☒	☐	The exact sample size (*n*) for each experimental group/condition, given as a discrete number and unit of measurement
☒	☐	A statement on whether measurements were taken from distinct samples or whether the same sample was measured repeatedly
☐	☒	The statistical test(s) used AND whether they are one- or two-sided<br>*Only common tests should be described solely by name; describe more complex techniques in the Methods section.*
☒	☐	A description of all covariates tested
☐	☒	A description of any assumptions or corrections, such as tests of normality and adjustment for multiple comparisons
☐	☒	A full description of the statistical parameters including central tendency (e.g. means) or other basic estimates (e.g. regression coefficient) AND variation (e.g. standard deviation) or associated estimates of uncertainty (e.g. confidence intervals)
☐	☒	For null hypothesis testing, the test statistic (e.g. *F*, *t*, *r*) with confidence intervals, effect sizes, degrees of freedom and *P* value noted<br>*Give P values as exact values whenever suitable.*
☒	☐	For Bayesian analysis, information on the choice of priors and Markov chain Monte Carlo settings
☒	☐	For hierarchical and complex designs, identification of the appropriate level for tests and full reporting of outcomes
☒	☐	Estimates of effect sizes (e.g. Cohen's *d*, Pearson's *r*), indicating how they were calculated

Our web collection on statistics for biologists contains articles on many of the points above.

Software and code

Policy information about availability of computer code

Data collection	No software was used in data collection.
Data analysis	All statistical analyses were conducted using R (version 4.3.2, R Core Team, Vienna, Austria). The R code used for statistical analysis and figure generation is also available at the GitHub repository (https://github.com/xmusphlkg/code_PHSM).

For manuscripts utilizing custom algorithms or software that are central to the research but not yet described in published literature, software must be made available to editors and reviewers. We strongly encourage code deposition in a community repository (e.g. GitHub). See the Nature Portfolio guidelines for submitting code & software for further information.

Data

Policy information about availability of data

All manuscripts must include a data availability statement. This statement should provide the following information, where applicable:

- Accession codes, unique identifiers, or web links for publicly available datasets
- A description of any restrictions on data availability
- For clinical datasets or third party data, please ensure that the statement adheres to our policy

The monthly incidence data for 24 NIDs used in this study are accessible through the GitHub repository (https://gthub.com/xmusphlkg/code_PHSM) and Supplementary Data 1. National-level data for all diseases are collected from http://www.nhc.gov.cn. Provincial-level data up to 2020 are collected from https://www.phsciencedata.cn/Share/ky_sjml.jsp, while data from 2021 onwards are sourced from various provincial health department websites, including https://

wjw.ah.gov.cn, https://wsjkw.cq.gov.cn, http://www.gscdc.net, http://wsjkw.gd.gov.cn, http://wsjkw.henan.gov.cn, http://wjw.jiangsu.gov.cn, http://wsjkw.shandong.gov.cn, https://wsjkw.sh.gov.cn, http://wsjkw.sc.gov.cn, https://wjw.xinjiang.gov.cn, and http://wsjkw.zj.gov.cn.

Research involving human participants, their data, or biological material

Policy information about studies with human participants or human data. See also policy information about sex, gender (identity/presentation), and sexual orientation and race, ethnicity and racism.

Reporting on sex and gender	The data was collected from the monthly Notifiable Infectious Diseases Reports and Chinese Public Health Science Data Center, gender and sex information were not reported.
Reporting on race, ethnicity, or other socially relevant groupings	The data was collected from the monthly Notifiable Infectious Diseases Reports and Chinese Public Health Science Data Center, this information were not reported.
Population characteristics	The data was collected from the monthly Notifiable Infectious Diseases Reports and Chinese Public Health Science Data Center, this information were not reported.
Recruitment	The data was collected from the monthly Notifiable Infectious Diseases Reports and Chinese Public Health Science Data Center, so there may be potential reporting bias.
Ethics oversight	Ethical approval was not required for data used in this study. And all data are publicly available thought the website of National Health Commission of China and Chinese Public Health Science Data Center.

Note that full information on the approval of the study protocol must also be provided in the manuscript.

Field-specific reporting

Please select the one below that is the best fit for your research. If you are not sure, read the appropriate sections before making your selection.

☒ Life sciences ☐ Behavioural & social sciences ☐ Ecological, evolutionary & environmental sciences

For a reference copy of the document with all sections, see nature.com/documents/nr-reporting-summary-flat.pdf

Life sciences study design

All studies must disclose on these points even when the disclosure is negative.

Sample size	The aim of the study was to explore the impact of public health and social measures on the 24 notifiable infectious diseases in China by December 2023. We collected all reported notifiable infectious diseases cases. From January 2008 to December 2023, 105,647,377 cases of 24 NIDs were reported in mainland China. IIDs were the most prevalent (45.24%), followed by BSTDs (31.10%) and RIDs (22.45%). The least reported were ZIDs (1.21%). Sample size is not applicable for our study.
Data exclusions	The disease selection criteria were determined based on data collected by China's NNDSS, which includes information from 31 provinces, excluding the Hong Kong Special Administrative Region (SAR), Macau SAR, and Taiwan province. The NNDSS was created in 2004 and has been significantly improved to monitor multiple NIDs. As of December 2023, data from 41 NIDs were included, but only 30 NIDs had more than 20,000 cases reported during the study period (from January 2008 to December 2023). COVID-19 and 5 other NIDs were excluded due to data deficiencies. Specifically, influenza was excluded from the analysis because its detection depends on specialized sentinel surveillance systems. Influenza A(H1N1) was virtually eliminated, and it was removed from monthly reports after November 2013 (Supplementary Data 1). "Other hepatitis" encompassing cases clinically diagnosed but not confirmed as hepatitis types A, B, C, D, or E, were also excluded from our analysis. Because the incidence of "other hepatitis" has been significantly influenced by the evolution of laboratory and hospital testing capabilities, technological advancements have led to a progressive decline of 'other hepatitis' cases (Supplementary Data 1). Moreover, diseases such as schistosomiasis and measles, which are nearing elimination in China and have been recently characterized by a relatively low prevalence, were also not considered in the analysis.
Replication	A single time series model alone is insufficient for capturing the epidemic patterns of all 24 diseases due to the diverse epidemiological characteristics and temporal distributions of different infectious diseases. Therefore, the ensemble forecasts include the neural network model, Bayesian structural time series model, Prophet model, ETS model, SARIMA model, and hybrid model (combine SARIMA, ETS, STL and neural network components). Each model has advantages depending on the specific epidemic characteristics of different diseases. The neural network model excels in capturing nonlinear trends and complex relationships. The Prophet model automatically handles long-term trends, seasonality, and holiday effects. Bayesian structural time series models address uncertainty and randomness; the ETS model is suitable for smoothing data and short-term forecasting; and the SARIMA model considers trends, seasonality, and autoregressive terms simultaneously. By combining the weighted averages of the neural network, STL, ETS, and SARIMA models, a hybrid model can better capture the epidemic trends of different infectious diseases.<br>All statistical analyses were conducted using R (version 4.3.2; R Core Team, Vienna, Austria).The R code and data is available at the GitHub repository (https://github.com/xmusphlkg/code_PHSM).
Randomization	Randomization was not applicable because this study is a observational study based on publicly available data.
Blinding	Blinding was not applicable because this study is a observational study based on publicly available data.

Reporting for specific materials, systems and methods

We require information from authors about some types of materials, experimental systems and methods used in many studies. Here, indicate whether each material, system or method listed is relevant to your study. If you are not sure if a list item applies to your research, read the appropriate section before selecting a response.

Materials & experimental systems

n/a	Involved in the study
☒	☐ Antibodies
☒	☐ Eukaryotic cell lines
☒	☐ Palaeontology and archaeology
☒	☐ Animals and other organisms
☒	☐ Clinical data
☒	☐ Dual use research of concern
☒	☐ Plants

Methods

n/a	Involved in the study
☒	☐ ChIP-seq
☒	☐ Flow cytometry
☒	☐ MRI-based neuroimaging

Plants

Seed stocks	Seed stocks was not applicable because this study was analysis human infectious diseases.
Novel plant genotypes	Novel plant genotypes was not applicable because this study was analysis human infectious diseases.
Authentication	This was not applicable because this study was analysis human infectious diseases.
